# Supplementary material for: CRISPR GENome and epigenome engineering improves loss-of-function genetic-screening approaches
Source: Cell Rep Methods. 2025 Jun 10;5(6):101078. doi: 10.1016/j.crmeth.2025.101078 (PMC12272254; doi:10.1016/j.crmeth.2025.101078)
Supplement: Document S1. Figures S1–S12 and Tables S1–S3 [file mmc1.pdf]

Cell Reports Methods, Volume *5*

Supplemental information

CRISPR GENome and epigenome engineering improves loss-of-function genetic-screening approaches

Jannis Stadager, Chiara Bernardini, Laura Hartmann, Henrik May, Jessica Wiepcke, Monika Kuban, Zeynab Najafova, Steven A. Johnsen, Stefan Legewie, Franziska R. Traube, Julian Jude, and Philipp Rathert

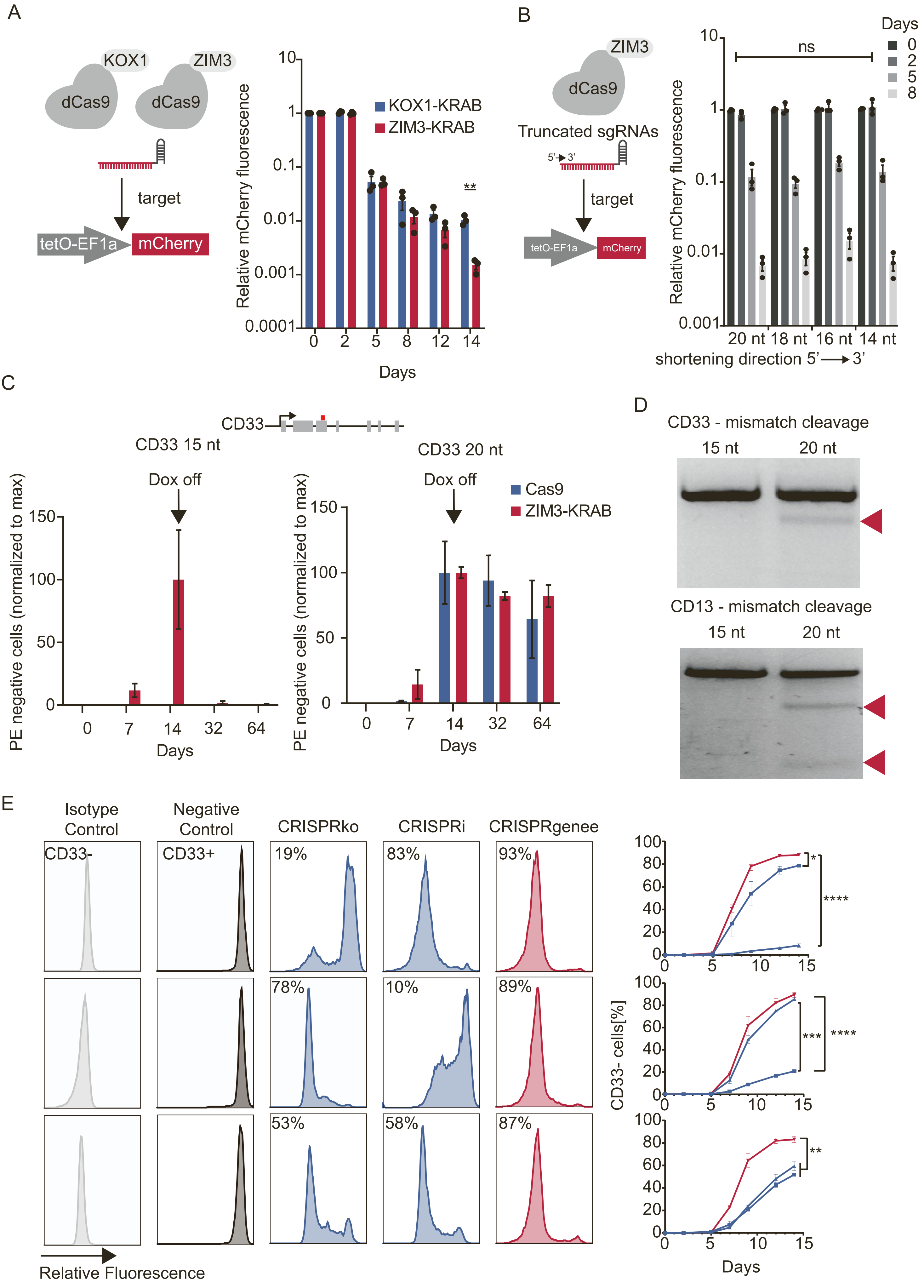

A
KOX1
ZIM3
dCas9
dCas9
target
tetO-EF1a
mCherry
Relative mCherry fluorescence
KOX1-KRAB
ZIM3-KRAB
**
Days
B
ZIM3
dCas9
Truncated sgRNAs
target
tetO-EF1a
mCherry
Relative mCherry fluorescence
ns
Days
0
2
5
8
20 nt 18 nt 16 nt 14 nt
shortening direction 5'→3'
C
CD33
CD33 15 nt
CD33 20 nt
Dox off
Dox off
Cas9
ZIM3-KRAB
PE negative cells (normalized to max)
Days
Days
D
CD33 - mismatch cleavage
15 nt
20 nt
CD13 - mismatch cleavage
15 nt
20 nt
E
Isotype Control
Negative Control
CRISPRko
CRISPRi
CRISPRgenee
CD33-
CD33+
19%
83%
93%
78%
10%
89%
53%
58%
87%
Relative Fluorescence
CD33- cells[%]
Days

Figure S1: Validation of the CRISPRgenee KRAB domain and setup of the proof of concept experiment. Related to Figure 1

(A) Flow-cytometric analysis of the mCherry expression in NIH/3T3 cells co-expressing the mCherry reporter, the dCas9-ZIM3 or dCas9-KOX1 fusion proteins and a sgRNA targeting the promoter region of the reporter. (n = 3, mean ± S.D..; **P ≤ 0.01, n.s. = non-significant; multiple t-tests with a two-stage set-up method of Benjamin to account for FDR).

(B) Flow-cytometric analysis of the mCherry expression in NIH/3T3 cells co-expressing the mCherry reporter, the dCas9-ZIM3 fusion protein, and truncated sgRNAs (5' to 3') with the indicated length targeting the promoter region of the reporter. (n = 3, mean ± S.D..; n.s. = non-significant, ordinary two-way ANOVA with Tukey post-hoc test).

(C) Quantification of CD33 expression by immunostaining of TF-1 erythroleukemia cells expressing Cas9 (blue) or ZIM3-Cas9 (red) and identical sgRNAs either 15 nt or 20 nt long. ZIM3-Cas9/Cas9 expression was induced for 14 days and afterward, on day 14 the percentage of CD33 negative cells was set as a reference, dox was removed and the recovery of the CD33 expression was monitored to determine the consistency of the induced genomic changes. (n = 3, mean ± S.D).

(D) Representative gel image of a DNA mismatch cleavage assay at the end of the experiment determining non-homologous end-joining efficiency for the indicated sgRNAs at the CD13 and CD33 genes. Red arrowheads indicate cleavage products due to mismatches between WT CD13/CD33 DNA and Cas9-targeted CD13/CD33 genes.

(E) Flow cytometry quantification of the isotype and non-targeting control as well as for different *CD33* targeting sgRNAs and the respective CRISPRgenee combination after 14 days as well as the overall time course of CD33 depletion (n = 3, mean ± s.e.m.; *P ≤ 0.05, **P ≤ 0.01, ***P ≤ 0.001, ****P ≤ 0.0001, n.s. = non-significant; two-way ANOVA with a Tukey post-hoc test).

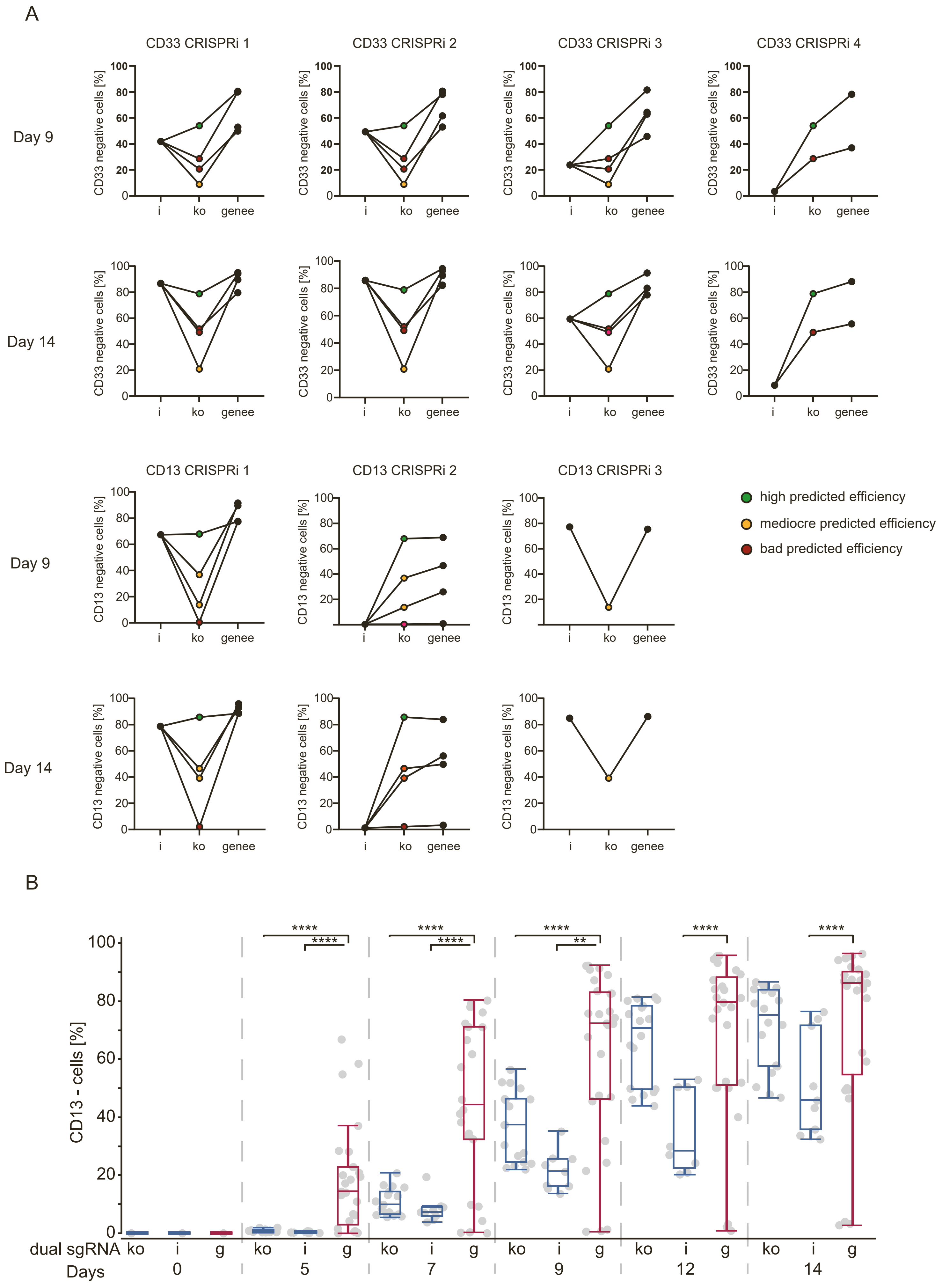
A
CD33 CRISPRi 1
CD33 CRISPRi 2
CD33 CRISPRi 3
CD33 CRISPRi 4
Day 9
Day 14
CD33 negative cells [%]
i
ko
genee
CD13 CRISPRi 1
CD13 CRISPRi 2
CD13 CRISPRi 3
CD13 negative cells [%]
high predicted efficiency
mediocre predicted efficiency
bad predicted efficiency
B
CD13 - cells [%]
****
**
dual sgRNA
ko
i
g
Days
0
5
7
9
12
14

Figure S2: CRISPRgenee combination efficiency and comparison to dual sgRNA CRISPRi/CRISPRko. Related to Figure 1

(A) CRISPR technology combination plot, median of the percentage of CD33/CD13 negative cells for the individual sgRNAs at day 9 and day 14 from figure 1E individually split for CRISPRi, CRISPRko, and the resulting indicated CRISPRgenee combinations (n = 3).

(B) Time-resolved quantification of CD13 negative TF-1 cells expressing dual sgRNAs targeting CD13 after induction of ZIM3-Cas9 (g), Cas9 (ko) or dCas9-ZIM3 (i). Data are displayed as a single datapoint for each sgRNA combination and replicate summarized in a boxplot. (n = 3, mean, box, and whiskers min to max.; $^{**}P \leq 0.01$, $^{***}P \leq 0.001$, n.s. = non-significant; two-way ANOVA with a Tukey post-hoc test).

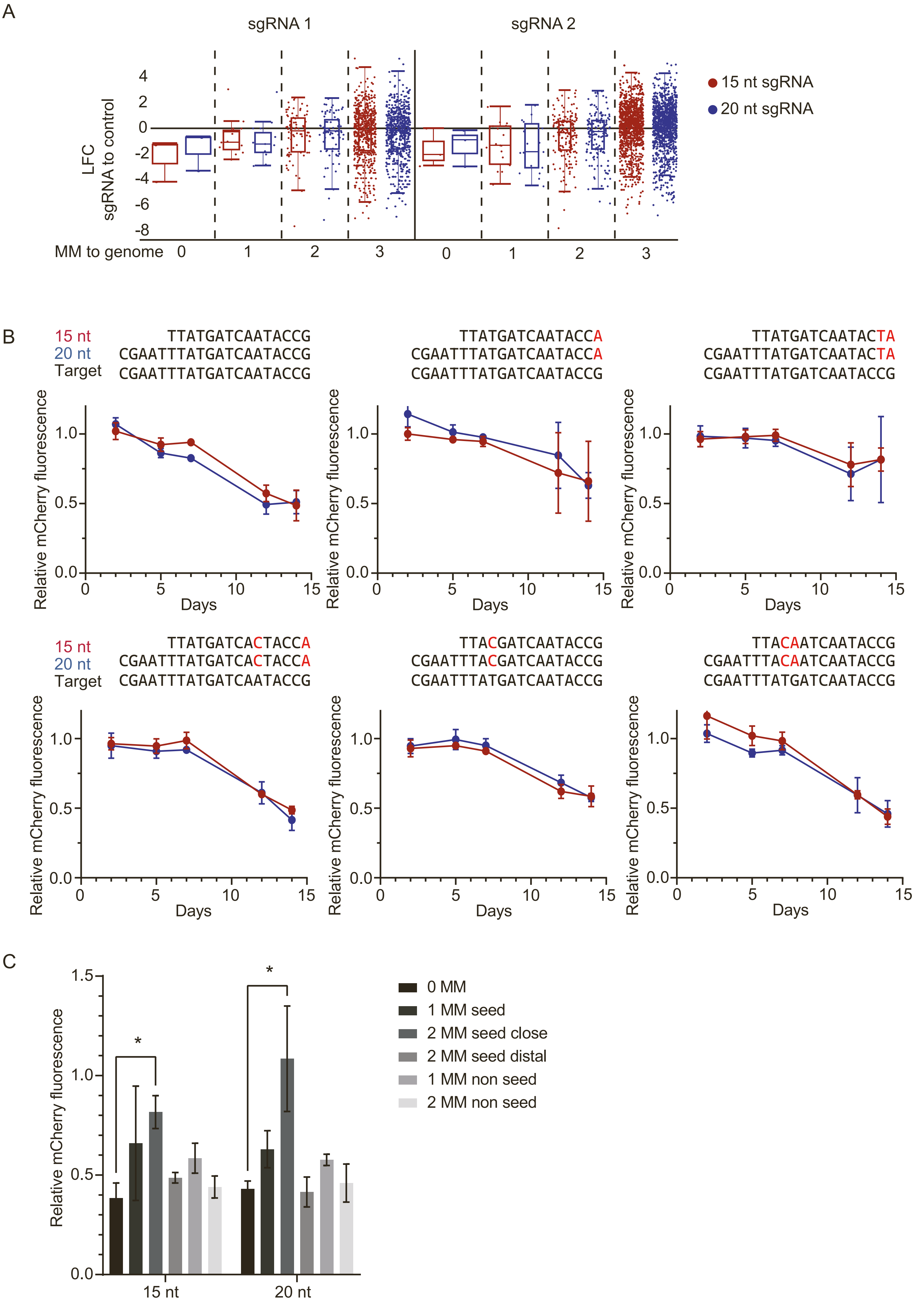
A
sgRNA 1
sgRNA 2
LFC
sgRNA to control
4
2
0
-2
-4
-6
-8
MM to genome
0
1
2
3
0
1
2
3
15 nt sgRNA
20 nt sgRNA
B
15 nt
20 nt
Target
TTATGATCAATACCG
CGAATTTATGATCAATACCG
CGAATTTATGATCAATACCG
TTATGATCAATACCA
CGAATTTATGATCAATACCA
CGAATTTATGATCAATACCG
TTATGATCAATACTA
CGAATTTATGATCAATACTA
CGAATTTATGATCAATACCG
Relative mCherry fluorescence
1.0
0.5
0.0
0
5
10
15
Days
15 nt
20 nt
Target
TTATGATCACTACCA
CGAATTTATGATCACTACCA
CGAATTTATGATCAATACCG
TTACGATCAATACCG
CGAATTTACGATCAATACCG
CGAATTTATGATCAATACCG
TTACAATCAATACCG
CGAATTTACAATCAATACCG
CGAATTTATGATCAATACCG
C
Relative mCherry fluorescence
1.5
1.0
0.5
0.0
*
*
15 nt
20 nt
0 MM
1 MM seed
2 MM seed close
2 MM seed distal
1 MM non seed
2 MM non seed

Figure S3: Off-target analysis of the 15 nt and 20 nt sgRNA targeting a fluorescent reporter. Related to figure 2

(A) Log2 foldchange between the 15 nt or 20 nt CD33 targeting sgRNA from Figure 2 and the control sgRNA for all identified off-targets (up to 3 MM) of the 15 nt sgRNA split into groups by the MM number.

(B) Flow-cytometric analysis of the mCherry expression in TF-1 cells co-expressing the mCherry reporter, the dCas9-ZIM3 as well as the indicated sgRNAs targeting the reporter. In red is indicated the respective off-target base towards the EF1a promoter expressing the mCherry.

(C) Comparison of the relative mCherry fluorescence at day 14 between the sgRNA mismatches for the 15 nt sgRNA and the 20 nt sgRNA ($n = 3$, mean ± SEM.; *$P \leq 0.05$, **$P \leq 0.01$, ****$P \leq 0.0001$; two-way ANOVA with a Sidak post-hoc test).

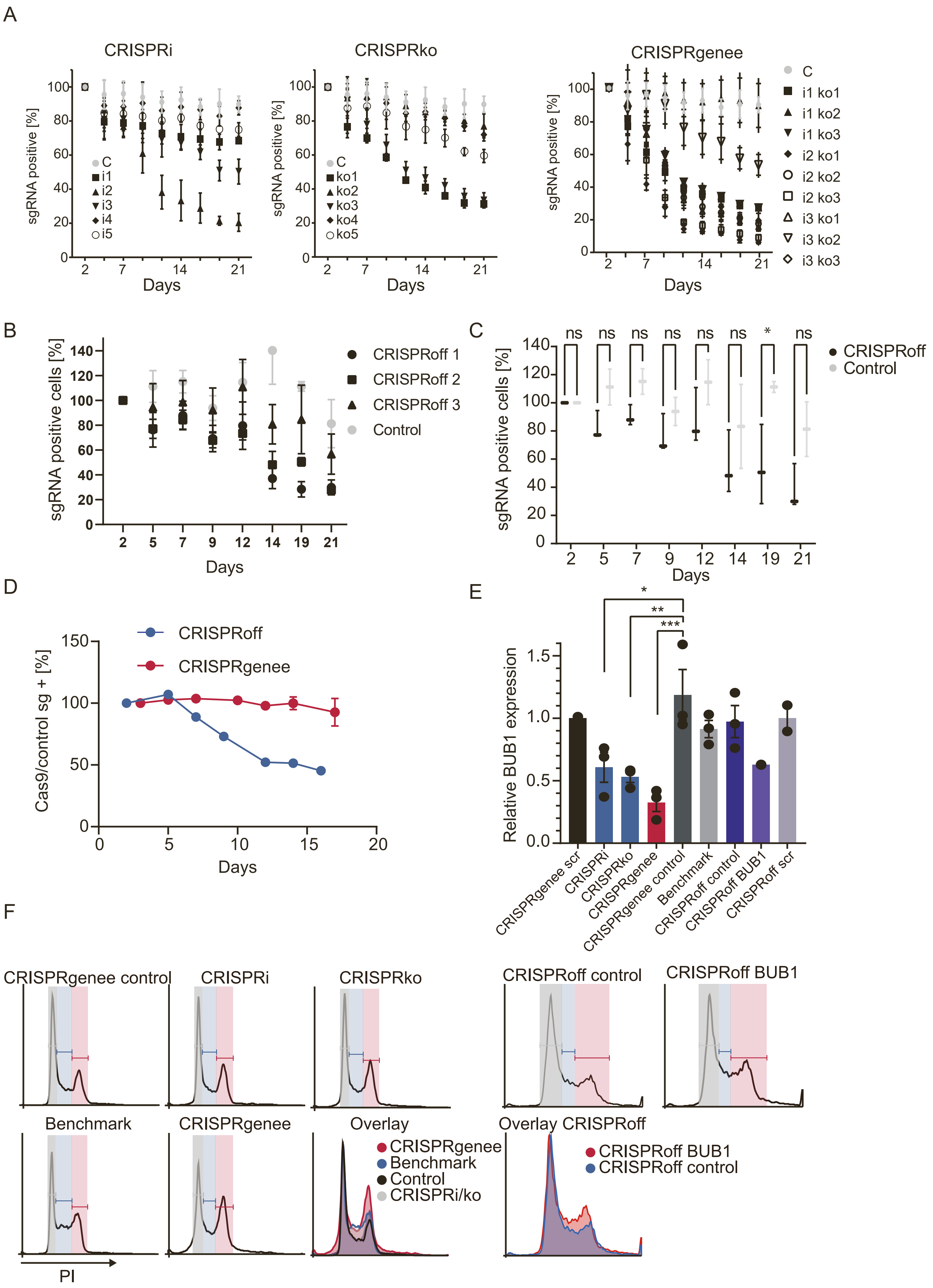

A
CRISPRi
CRISPRko
CRISPRgenee
sgRNA positive [%]
Days
C
i1
i2
i3
i4
i5
ko1
ko2
ko3
ko4
ko5
i1 ko1
i1 ko2
i1 ko3
i2 ko1
i2 ko2
i2 ko3
i3 ko1
i3 ko2
i3 ko3
B
sgRNA positive cells [%]
CRISPRoff 1
CRISPRoff 2
CRISPRoff 3
Control
C
ns
*
CRISPRoff
D
Cas9/control sg + [%]
CRISPRoff
CRISPRgenee
E
Relative BUB1 expression
*
**
***
CRISPRgenee scr
CRISPRi
CRISPRko
CRISPRgenee
CRISPRgenee control
Benchmark
CRISPRoff control
CRISPRoff BUB1
CRISPRoff scr
F
CRISPRgenee control
CRISPRi
CRISPRko
CRISPRoff control
CRISPRoff BUB1
Benchmark
CRISPRgenee
Overlay
Overlay CRISPRoff
Control
CRISPRi/ko
PI

Figure S4: Validation of single *BUB1* targeting sgRNAs and BUB1 depletion efficiency. Related to Figure 3

(A) Raw data from figure 3A showing the individual competitive proliferation assays of TF-1 cells expressing the indicated single sgRNAs as well as the respective CRISPRgenee combinations targeting *BUB1*. Shown is the relative fraction of GFP+/sgRNA+ cells relative to the initial measurement over the course of 21 days. (n = 3, mean ± s.e.m.)

(B) Raw data of the individual dual CRISPRoff sgRNAs targeting *BUB1* and the control targeting a non-essential gene.

(C) Summary of the proliferative effect of *BUB1* targeting sgRNAs for CRISPRoff and the respective control targeting a non-essential gene.

(D) Percentage of cells co-expressing CRISPRoff/CRISPRgenee and a non-targeting sgRNA control.

(E) Relative *BUB1* expression of HEK293 cells expressing sgRNAs targeting *BUB1* normalized to b-actin and a neutral control sgRNA to validate the improved CRISPRgenee effect observed in the proliferation assays. (n = 3, mean ± S.D..; *P ≤ 0.05, **P ≤ 0.01, ***P ≤ 0.001, n.s. = non-significant; two-way ANOVA with a Dunnet post-hoc test).

(F) Exemplary histograms of the P.I. staining of the individual samples and the corresponding distribution in G1- (grey), S- (blue), and G2/M-phase (red). An overlay of all histograms was plotted to further highlight the difference in cell cycle distribution.

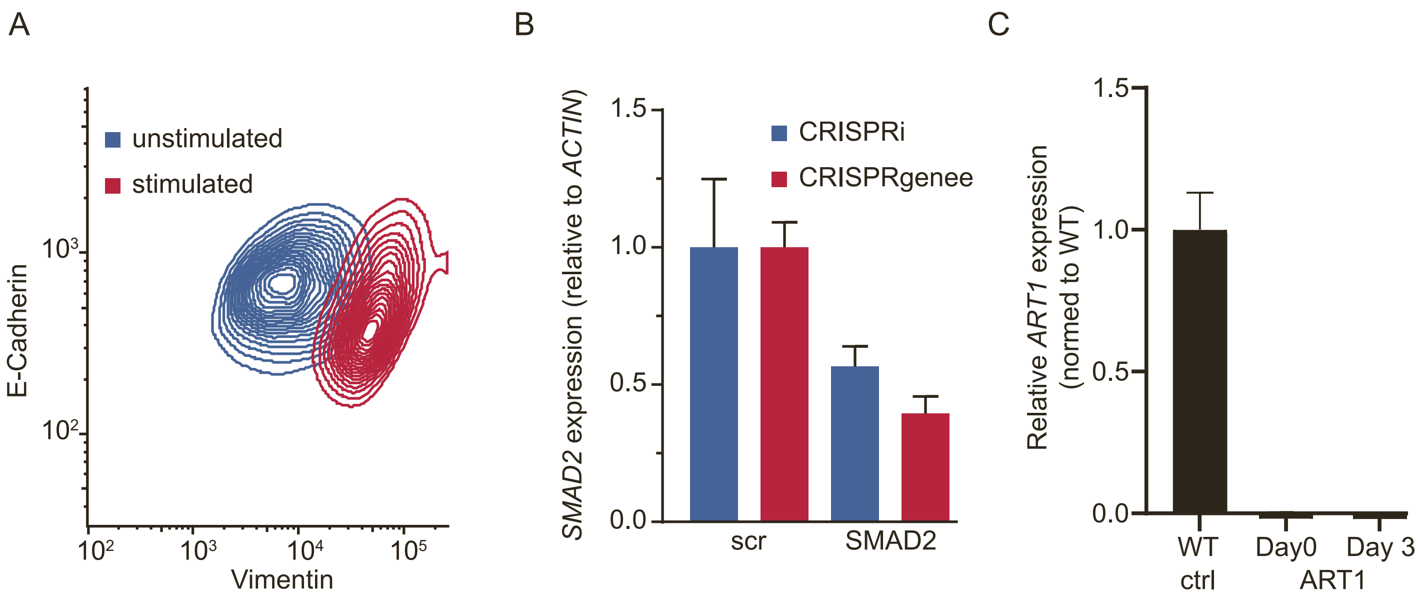
A
unstimulated
stimulated
E-Cadherin
Vimentin
B
SMAD2 expression (relative to ACTIN)
CRISPRi
CRISPRgenee
scr
SMAD2
C
Relative ART1 expression (normed to WT)
WT ctrl
Day0
Day 3
ART1

Figure S5: Staining and depletion validation for EMT and iPSCs. Related to Figure 3
(A) MCF10A cells were stained with antibodies binding to the intracellular proteins Vimentin and E-Cadherin used to identify and gate for the epithelial cell population (blue) and mesenchymal cell population (red).
(B) Relative *SMAD2* expression of MCF10A cells expressing sgRNAs targeting a non-essential control or *SMAD2* normalized to b-actin to validate the loss of SMAD2.
(C) Relative ART1 expression of unstimulated and stimulated iPSC cells expressing CRISPRgenee and a sgRNA combination targeting ART1 normalized to *b-Actin* and WT.

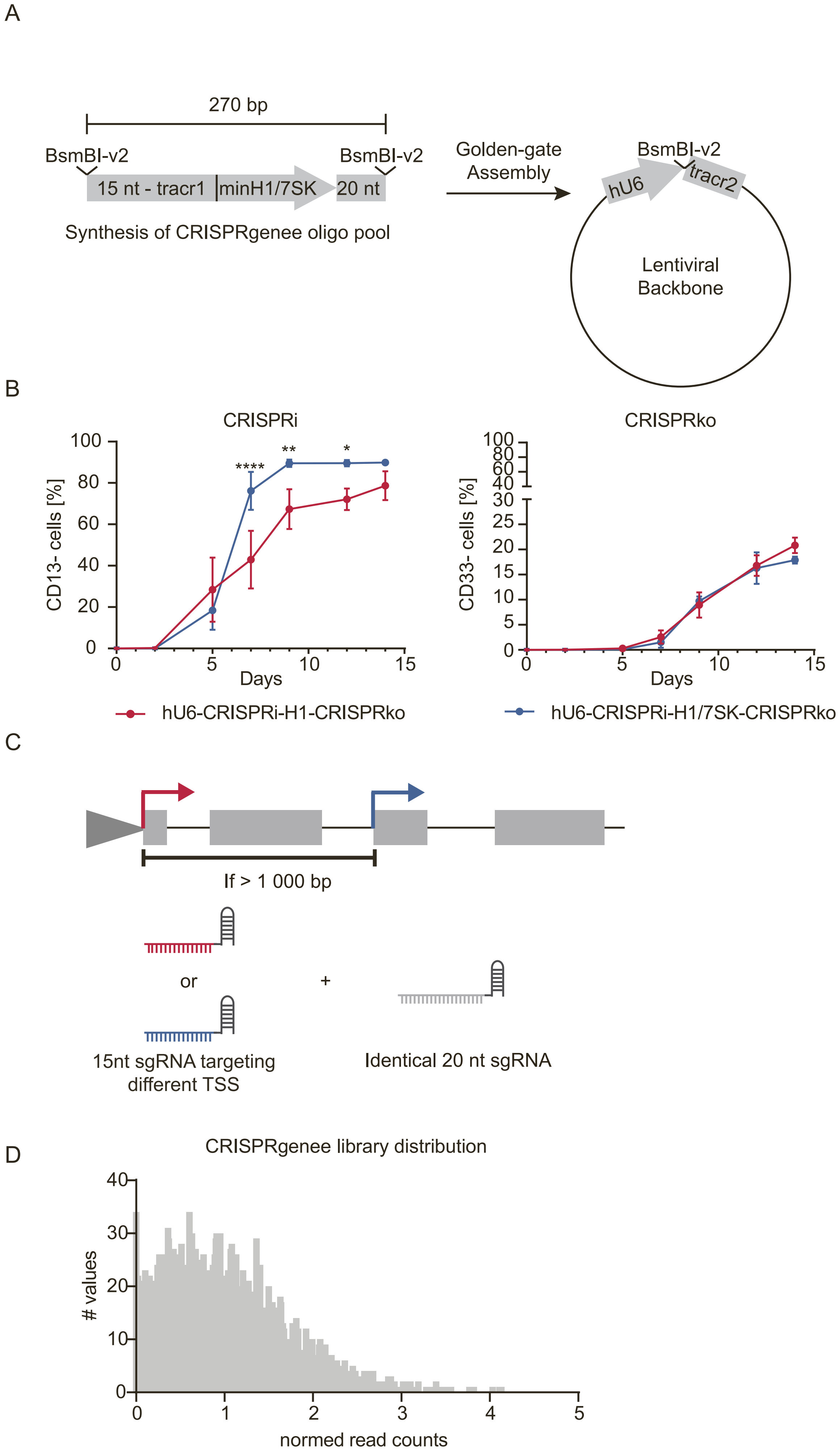
A
270 bp
BsmBI-v2
BsmBI-v2
15 nt - tracr1
minH1/7SK
20 nt
Synthesis of CRISPRgenee oligo pool
Golden-gate Assembly
BsmBI-v2
hU6
tracr2
Lentiviral Backbone
B
CRISPRi
CD13- cells [%]
Days
****
**
*
CRISPRko
CD33- cells [%]
Days
hU6-CRISPRi-H1-CRISPRko
hU6-CRISPRi-H1/7SK-CRISPRko
C
If > 1 000 bp
or
+
15nt sgRNA targeting different TSS
Identical 20 nt sgRNA
D
CRISPRgenee library distribution
values
normed read counts

Figure S6: CRISPRgenee library design. Related to Figure 4
(A) Oligo design of the CRISPRgenee library and the subsequent cloning strategy into the lentiviral backbone.
(B) Time-resolved quantification of dual CD13 and CD33 depletion in TF-1 cells using the hU6 promoter to express identical CD13 sgRNAs as well as the H1 or minimal H1/7SK promoter to express the CD33 targeting sgRNA (n = 3, mean ± S.D.; *P ≤ 0.05, **P ≤ 0.01, ****P ≤ 0.0001; two-way ANOVA with a Sidak post-hoc test).
(C) CRISPRgenee combination strategy for genes harbouring more than one TSS with a distance of > 1000 bp.
(D) CRISPRgenee sgRNA library distribution, measured and calculated by deep-sequencing.

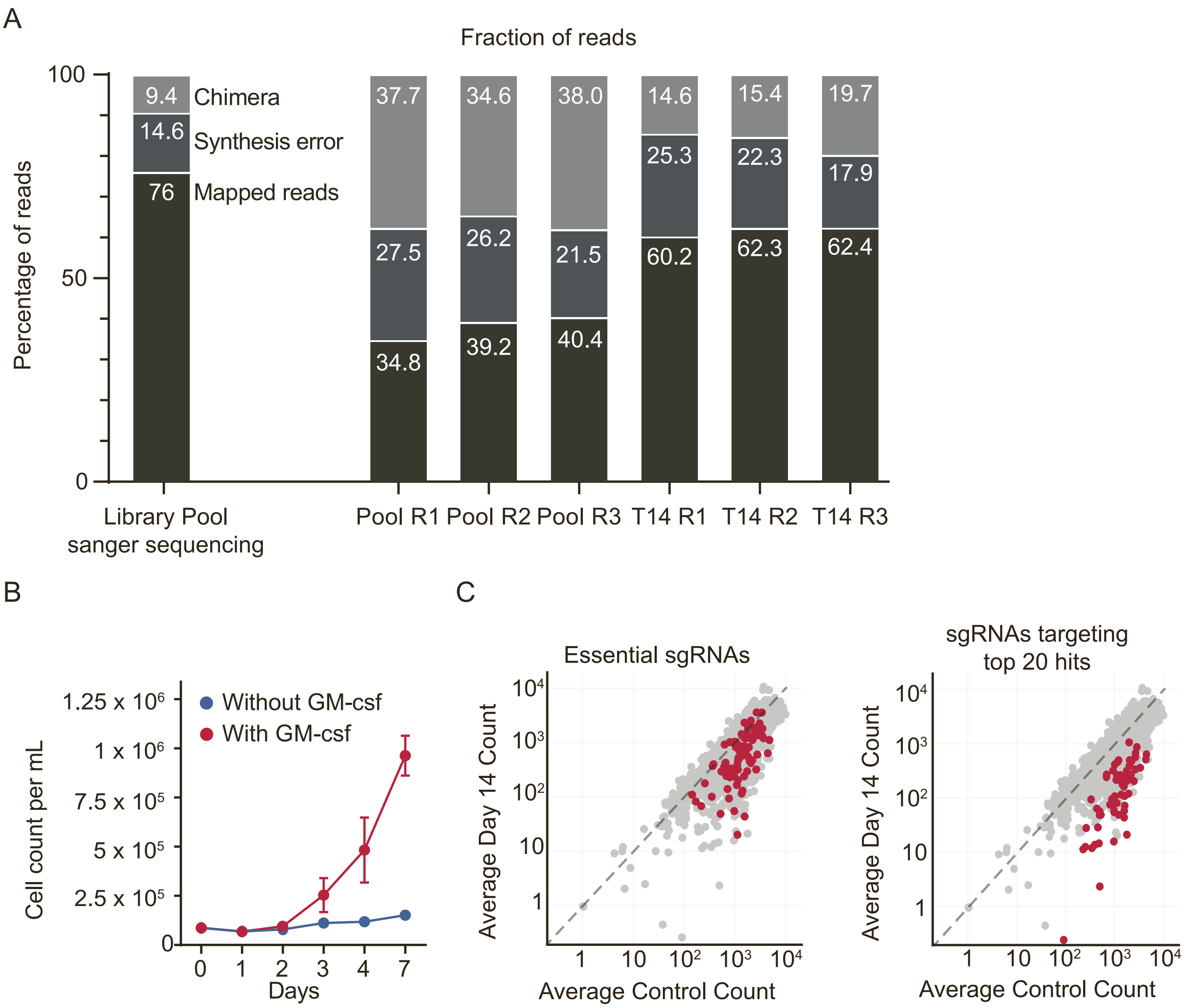
A
Fraction of reads
Percentage of reads
100
50
0
9.4
Chimera
14.6
Synthesis error
76
Mapped reads
37.7
27.5
34.8
34.6
26.2
39.2
38.0
21.5
40.4
14.6
25.3
60.2
15.4
22.3
62.3
19.7
17.9
62.4
Library Pool
sanger sequencing
Pool R1
Pool R2
Pool R3
T14 R1
T14 R2
T14 R3
B
Cell count per mL
1.25 x 10⁶
1 x 10⁶
7.5 x 10⁵
5 x 10⁵
2.5 x 10⁵
0
Without GM-csf
With GM-csf
0 1 2 3 4 7
Days
C
Essential sgRNAs
Average Day 14 Count
Average Control Count
sgRNAs targeting
top 20 hits
Average Day 14 Count
Average Control Count

Figure S7: Chimera rate and reproducibility of the CRISPRgenee library. Related to Figure 4

(A) Attributed mapped reads, synthesis error reads, and the chimera rate of the oligo pool replicates and the day 14 replicates of the CRISPRgenee screen after library preparation and sequencing.

(B) Proliferation of TF-1 cells grown in RPMI media supplemented with or without the growth factor GM-CSF.

(C) Left: normalized read counts of the sgRNAs targeting essential genes for the library pool after 14 days of screening. Right: normalized read counts of sgRNAs targeting the top 20 gene hits identified in the CRISPRgenee screen of the library pool compared to day 14 after induction of ZIM3-Cas9 expression.

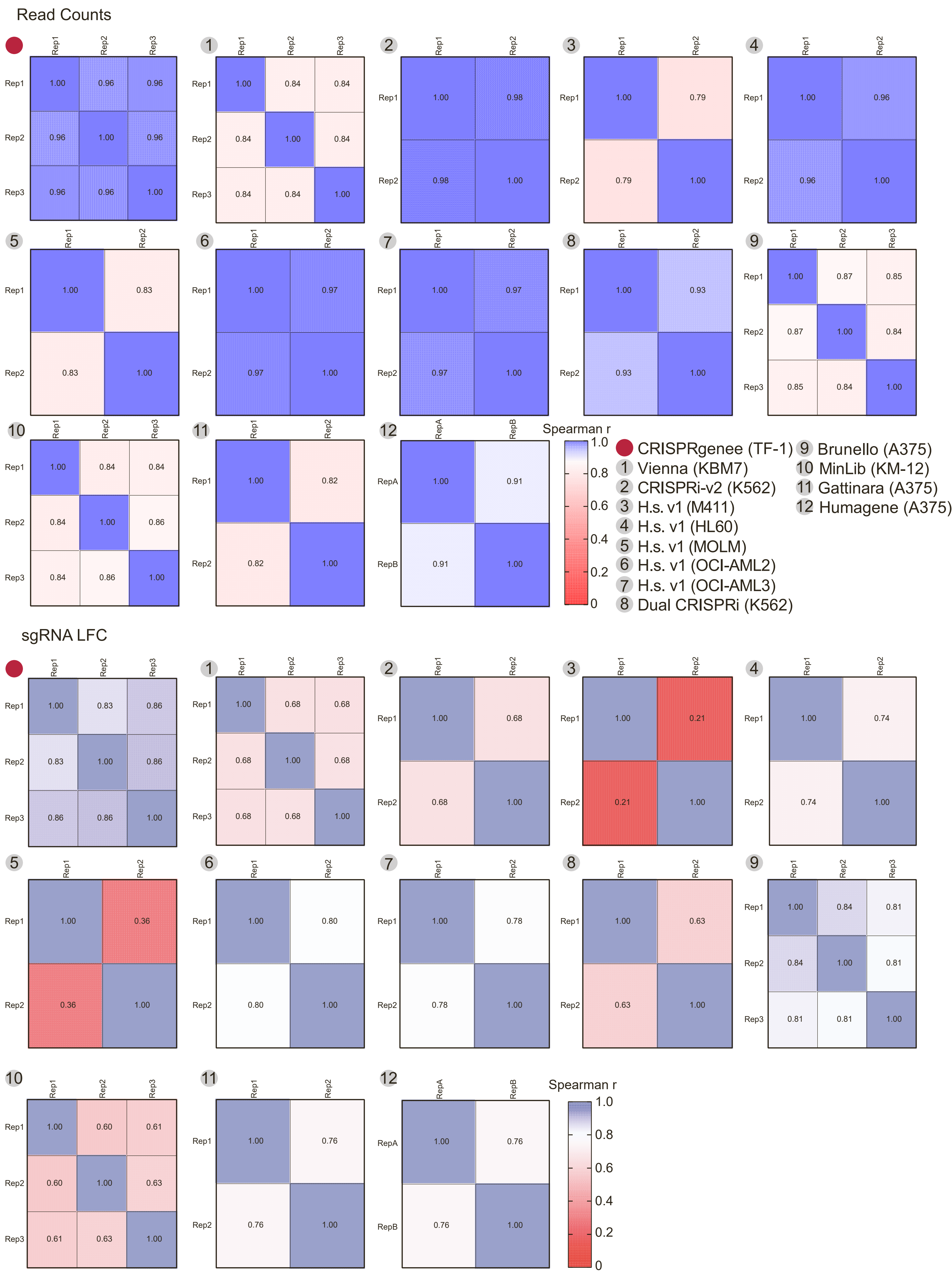
Read Counts
Spearman r
1.0
0.8
0.6
0.4
0.2
0
CRISPRgenee (TF-1)
1 Vienna (KBM7)
2 CRISPRi-v2 (K562)
3 H.s. v1 (M411)
4 H.s. v1 (HL60)
5 H.s. v1 (MOLM)
6 H.s. v1 (OCI-AML2)
7 H.s. v1 (OCI-AML3)
8 Dual CRISPRi (K562)
9 Brunello (A375)
10 MinLib (KM-12)
11 Gattinara (A375)
12 Humagene (A375)
sgRNA LFC
Spearman r
1.0
0.8
0.6
0.4
0.2
0

Figure S8: CRISPRgenee achieves better reproducibility on read count and sgRNA LFC level. Related to Figure 4

Heat maps for each CRISPR screen analyzed in Figure 4D. Top: Spearman correlation of read counts of individual screening replicates for each analyzed screen. Bottom: Spearman correlation of the sgRNA LFC of individual screening replicates for each analyzed screen.

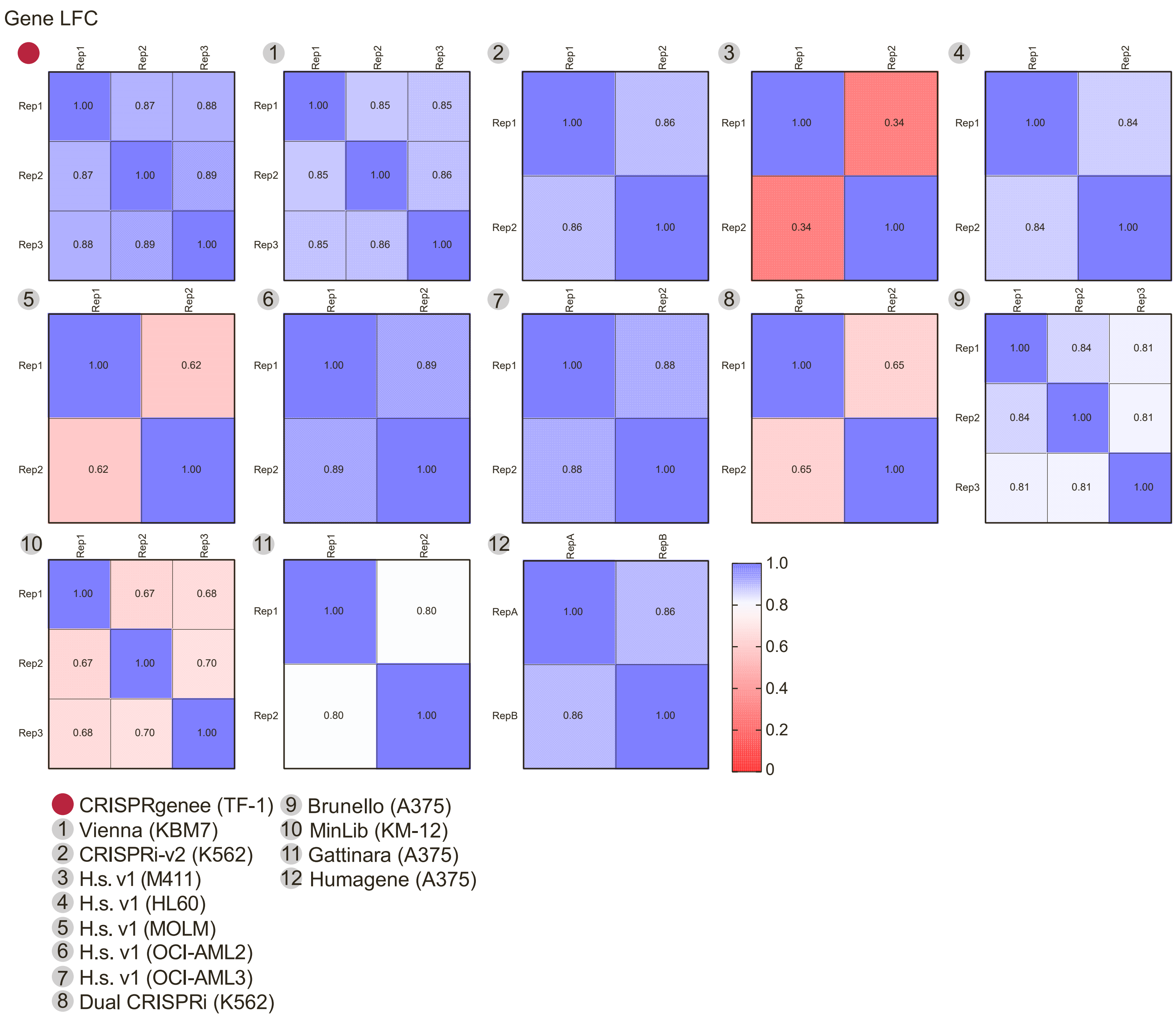

Gene LFC
Rep1 Rep2 Rep3
Rep1 1.00 0.87 0.88
Rep2 0.87 1.00 0.89
Rep3 0.88 0.89 1.00
1
Rep1 1.00 0.85 0.85
Rep2 0.85 1.00 0.86
Rep3 0.85 0.86 1.00
2
Rep1 1.00 0.86
Rep2 0.86 1.00
3
Rep1 1.00 0.34
Rep2 0.34 1.00
4
Rep1 1.00 0.84
Rep2 0.84 1.00
5
Rep1 1.00 0.62
Rep2 0.62 1.00
6
Rep1 1.00 0.89
Rep2 0.89 1.00
7
Rep1 1.00 0.88
Rep2 0.88 1.00
8
Rep1 1.00 0.65
Rep2 0.65 1.00
9
Rep1 1.00 0.84 0.81
Rep2 0.84 1.00 0.81
Rep3 0.81 0.81 1.00
10
Rep1 1.00 0.67 0.68
Rep2 0.67 1.00 0.70
Rep3 0.68 0.70 1.00
11
Rep1 1.00 0.80
Rep2 0.80 1.00
12
RepA RepB
RepA 1.00 0.86
RepB 0.86 1.00
1.0 0.8 0.6 0.4 0.2 0
CRISPRgenee (TF-1)
1 Vienna (KBM7)
2 CRISPRi-v2 (K562)
3 H.s. v1 (M411)
4 H.s. v1 (HL60)
5 H.s. v1 (MOLM)
6 H.s. v1 (OCI-AML2)
7 H.s. v1 (OCI-AML3)
8 Dual CRISPRi (K562)
9 Brunello (A375)
10 MinLib (KM-12)
11 Gattinara (A375)
12 Humagene (A375)

Figure S9: CRISPRgenee achieves better reproducibility on gene LFC level.
Related to Figure 4
Heat maps for each CRISPR screen analysed in Figure 4D in which the gene LFC of individual screening replicates per screen were correlated using the spearman correlation analysis.

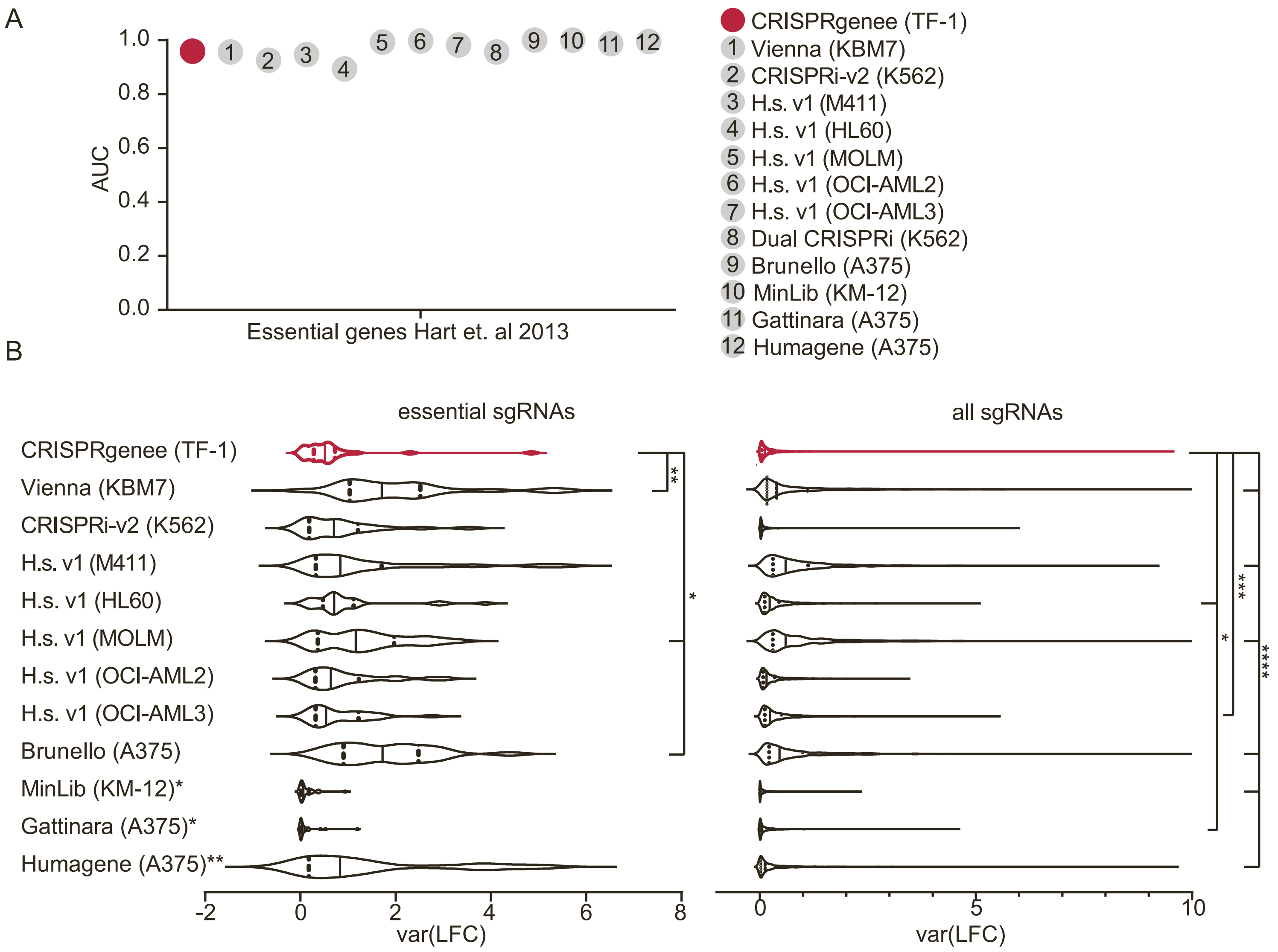

*<3 sgRNAs per gene; **enAsCas12a

Figure S10: CRISPRgenee demonstrates high sensitivity with increased consistency in sgRNA performance. Related to Figure 4

(A) The area under the curve (AUC) of ROC curves based on essential and non-essential genes for the CRISPRgenee screen and other published screens shown in Figure 4C.

(B) Violin plots comparing the variance among sgRNAs targeting the same gene observed with the CRISPRgenee system with a set of published CRISPR screening approaches. Left: Comparison of the variance of sgRNAs targeting the same genes from a subset of essential genes. Right: Comparison of the variance of sgRNAs targeting the same genes for all genes investigated in the CRISPRgenee screen.

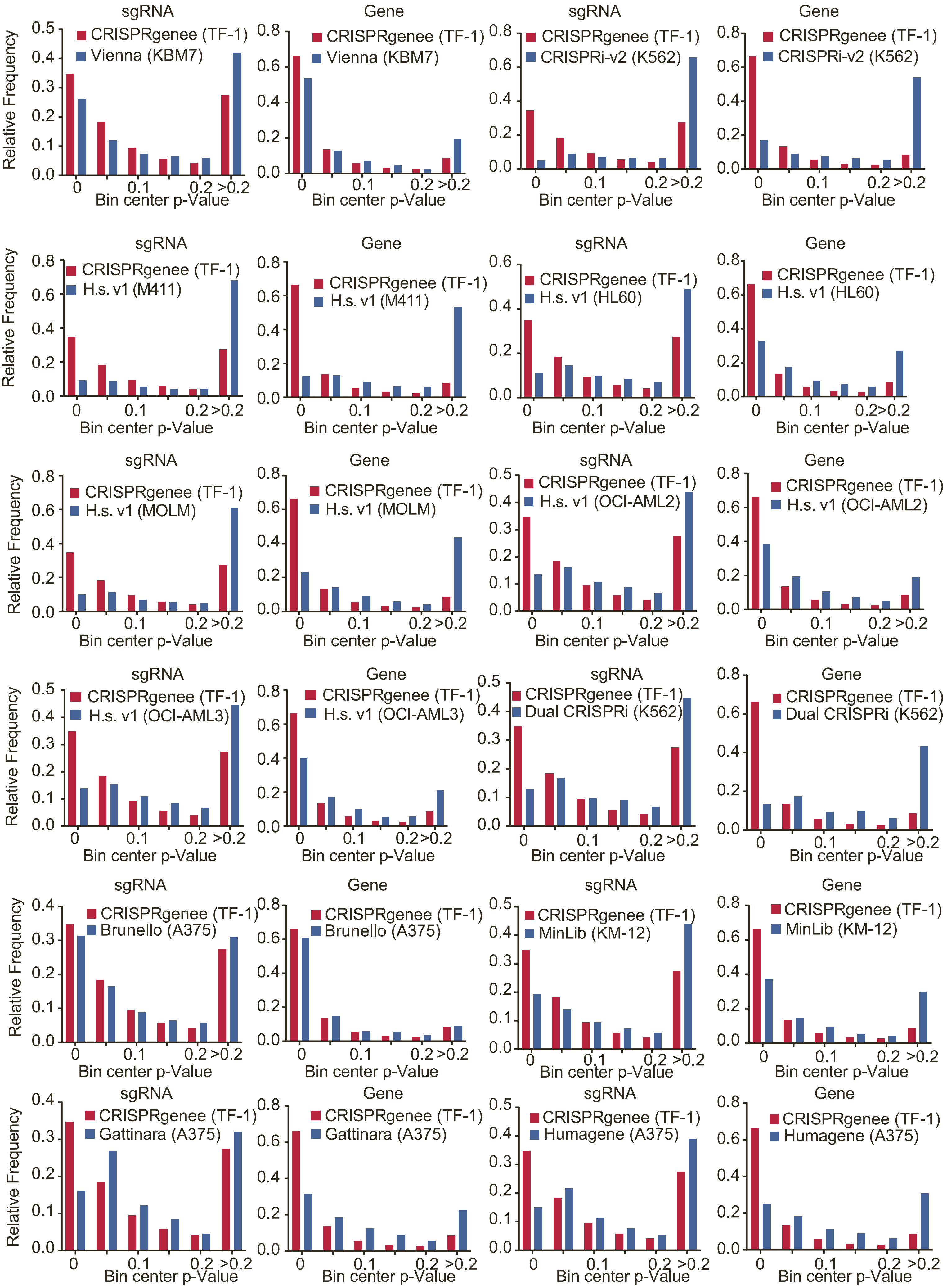
sgRNA
CRISPRgenee (TF-1)
Vienna (KBM7)
Gene
CRISPRgenee (TF-1)
Vienna (KBM7)
sgRNA
CRISPRgenee (TF-1)
CRISPRi-v2 (K562)
Gene
CRISPRgenee (TF-1)
CRISPRi-v2 (K562)
sgRNA
CRISPRgenee (TF-1)
H.s. v1 (M411)
Gene
CRISPRgenee (TF-1)
H.s. v1 (M411)
sgRNA
CRISPRgenee (TF-1)
H.s. v1 (HL60)
Gene
CRISPRgenee (TF-1)
H.s. v1 (HL60)
sgRNA
CRISPRgenee (TF-1)
H.s. v1 (MOLM)
Gene
CRISPRgenee (TF-1)
H.s. v1 (MOLM)
sgRNA
CRISPRgenee (TF-1)
H.s. v1 (OCI-AML2)
Gene
CRISPRgenee (TF-1)
H.s. v1 (OCI-AML2)
sgRNA
CRISPRgenee (TF-1)
H.s. v1 (OCI-AML3)
Gene
CRISPRgenee (TF-1)
H.s. v1 (OCI-AML3)
sgRNA
CRISPRgenee (TF-1)
Dual CRISPRi (K562)
Gene
CRISPRgenee (TF-1)
Dual CRISPRi (K562)
sgRNA
CRISPRgenee (TF-1)
Brunello (A375)
Gene
CRISPRgenee (TF-1)
Brunello (A375)
sgRNA
CRISPRgenee (TF-1)
MinLib (KM-12)
Gene
CRISPRgenee (TF-1)
MinLib (KM-12)
sgRNA
CRISPRgenee (TF-1)
Gattinara (A375)
Gene
CRISPRgenee (TF-1)
Gattinara (A375)
sgRNA
CRISPRgenee (TF-1)
Humagene (A375)
Gene
CRISPRgenee (TF-1)
Humagene (A375)
Relative Frequency
Bin center p-Value
0
0.1
0.2
>0.2

Figure S11: CRISPRgenee achieves more significant hits on sgRNA and gene level. Related to Figure 4
Histograms comparing the relative p-value frequencies of sgRNAs as well as the p-value for each gene in the CRISPRgenee screen with a set of published CRISPR screening approaches.

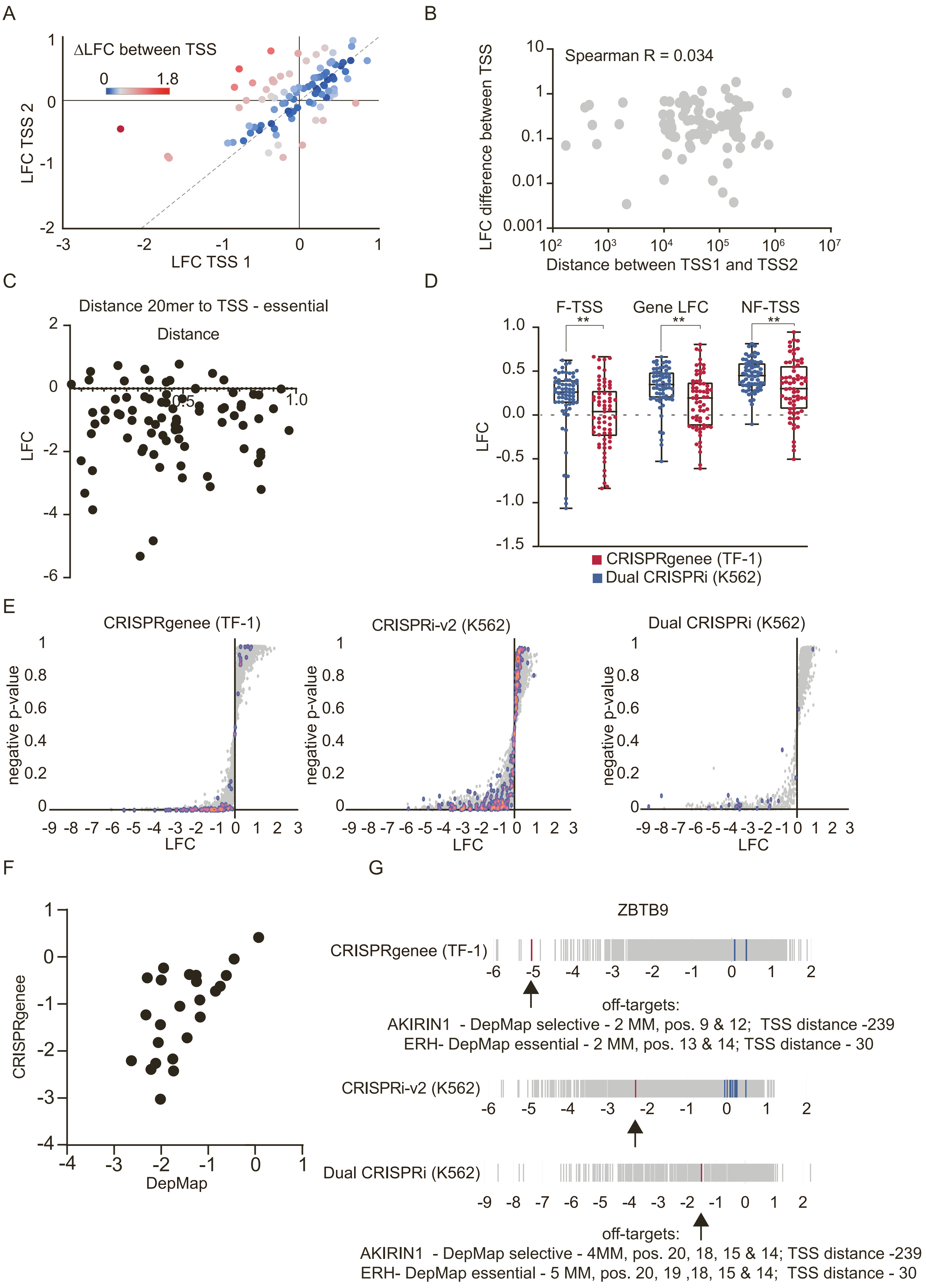
A
ΔLFC between TSS
0
1.8
LFC TSS 2
LFC TSS 1
B
Spearman R = 0.034
LFC difference between TSS
Distance between TSS1 and TSS2
C
Distance 20mer to TSS - essential
Distance
LFC
D
F-TSS
Gene LFC
NF-TSS
**
LFC
CRISPRgenee (TF-1)
Dual CRISPRi (K562)
E
CRISPRgenee (TF-1)
CRISPRi-v2 (K562)
Dual CRISPRi (K562)
negative p-value
LFC
F
CRISPRgenee
DepMap
G
ZBTB9
CRISPRgenee (TF-1)
off-targets:
AKIRIN1 - DepMap selective - 2 MM, pos. 9 & 12; TSS distance -239
ERH- DepMap essential - 2 MM, pos. 13 & 14; TSS distance - 30
CRISPRi-v2 (K562)
Dual CRISPRi (K562)
off-targets:
AKIRIN1 - DepMap selective - 4MM, pos. 20, 18, 15 & 14; TSS distance -239
ERH- DepMap essential - 5 MM, pos. 20, 19 ,18, 15 & 14; TSS distance - 30

Figure S12: CRISPRi efficiency depends on the TSS selection. Related to Figure 5 and Figure 6

(A) Scatter plot depicting the LFC of genes which have more than one TSS and are 1 000 bp distant from each other split into the cumulative LFC for sgRNAs targeting one TSS and sgRNAs targeting the other. The ΔLFC was calculated between the two TSSs and is displayed with a blue-to-red color gradient depending on the ΔLFC.

(B) Relationship between the ΔLFC of two different TSS of the same gene and the distance between both TSSs.

(C) Distance of the CRISPRko sgRNA to the TSS plotted against the LFC to identify if the CRISPRko sgRNA is also affecting gene expression through the ZIM3 domain during Cas9 binding and cleavage.

(D) The difference in LFC between the CRISPRgenee screen and the dual CRISPRi screen when targeting only the functional (F) TSS, the non-functional (NF) TSS, or the cumulative LFC for each gene.

(E) Plot of the p-value for each sgRNA over the LFC of the sgRNA for CRISPRgenee, a CRISPRi screen (16) and a dual CRISPRi screen (28). The sgRNAs targeting essential genes are displayed as density plot in red.

(F) Plot of the CRISPRgenee LFC over the DepMap score for all essential genes targeted in the CRISPRgenee screen.

(G) LFC for the CRISPRgenee sgRNA combinations targeting *ZBTB9*.

Table S1: List of all oligonucleotides used in this study for cloning, qPCR, mismatch cleavage assay, amplicon and CRISPR library sequencing. Related to Figure 1, 3 and 4 and Star Methods.

Name	Sequence (5' to 3')	Experiment
ZIM3-KRAB_Gibson_for	GGATCCACCAGCATGAACAATTCCCAGGGAAGAGTGACCTTCG	Cloning
ZIM3-KRAB_Gibson_rev	TTCTTGTCCATGGTGGCAGCGCTGATCCCGGGCCCGCGGTA	Cloning
Dual_Filler_Gibson_for	CACCTGCGTGGCACCAGA	Cloning
Dual_Filler_Gibson_rev	CACCTGCTCTCAAACGGAGA	Cloning
CRISPRoff_Gibson_for	CTTGCGTTGGATCCACCAGCCTGCAGTCGACGGTACCG	Cloning
CRISPRoff_Gibson_rev	TACCGATAAGCTTGATATCGCTAGGGCTCTTCTCCCTTC	Cloning
CD33_forward1_CTCA	CTCTTTCCCTACACGACGCTCTTCCGATCTNNNNNNNCTCAGTGAGCAGGGAACACCCCC	Amplicon Sequencing CD33 Control PCR1
CD33_forward1_TGAG	CTCTTTCCCTACACGACGCTCTTCCGATCTNNNNNNNTGAGGTGAGCAGGGAACACCCCC	Amplicon Sequencing CD33 15mer PCR1
CD33_forward1_TCGA	CTCTTTCCCTACACGACGCTCTTCCGATCTNNNNNNNTCGAGTGAGCAGGGAACACCCCC	Amplicon Sequencing CD33 20mer PCR1
CD33_reverse1	GTGACTGGAGTTCAGACGTGTGCTCTTCCGATCTCTGGGGCCGTGGGGTGATTA	Amplicon Sequencing CD33 PCR1
CD33_CD13_forward2	AATGATACGGCGACCACCGAGATCTACACTAGATCGCACACTCTTTCCCTACACGACGCTC	Amplicon Sequencing CD33 or CD13 PCR2
CD33_CD13_reverse2	CAAGCAGAAGACGGCATACGAGATATCACGGTGACTGGAGTTCAGACGTGTG	Amplicon Sequencing CD33 or CD13 PCR2
CD13_forward1_TAGG	CTCTTTCCCTACACGACGCTCTTCCGATCTNNNNNNNTAGGACCTGGGTGCTGACTATG	Amplicon Sequencing CD13 Control PCR1
CD13_forward1_GTTC	CTCTTTCCCTACACGACGCTCTTCCGATCTNNNNNNNGTTCACCTGGGTGCTGACTATG	Amplicon Sequencing CD13 15mer PCR1
CD13_forward1_GGAT	CTCTTTCCCTACACGACGCTCTTCCGATCTNNNNNNNGGATACCTGGGTGCTGACTATG	Amplicon Sequencing CD13 20mer PCR1
CD13_reverse1	GTGACTGGAGTTCAGACGTGTGCTCTTCCGATCTAGGAGATGGCGTCAAACAG	Amplicon Sequencing CD13 PCR1
qPCR_B-actin_for	AGAAAATCTGGCACCACACC	qPCR
qPCR_B-actin_rev	AGAGGCGTACAGGGATAGCA	qPCR
BUB1 Exon 23-24_for	CGATTACTTTGGGGTTGCTG	qPCR
BUB1 Exon 23-24_rev	GCTTTTGCCTTAACAAATCCA	qPCR
SMAD2_for	GGGTTTTGAAGCCGTCTATCAGC	qPCR
SMAD2_rev	CCAACCACTGTAGAGGTCCATTC	qPCR
ART1_for	GATGCCTGCTATGATGTCTCTG	qPCR
ART1_rev	AGAAGAGGTCTCGTCGTGTGA	qPCR
Syn1_for	AGCTCAACAAATCCCAGTCTCT	qPCR

Syn1_rev	CGGATGGTCTCAGCTTTCAC	qPCR
CD33_for	ACTTTCTTCCATCCCATACCCT	Missmatch cleavage assay
CD33_rev	GGATGGTTCTCTCCGTAGTCAC	Missmatch cleavage assay
CD13_for	TAGAGTGGTGGAATGACCTGTG	Missmatch cleavage assay
CD13_rev	GTAGGCAAAGGTGTGGAGGTAG	Missmatch cleavage assay
sggeneeSeq5_rev_GGAT	CTCTTTCCCTACACGACGCTCTTCCGATCTNNNNNNATCCTGTTTCCAGCATAGCTCTT	Library Aplification PCR1
sggeneeSeq7_rev_GAAG	CTCTTTCCCTACACGACGCTCTTCCGATCTNNNNNNCTTCTGTTTCCAGCATAGCTCTT	Library Aplification PCR1
sggeneeSeq8_rev_CTTG	CTCTTTCCCTACACGACGCTCTTCCGATCTNNNNNNCAAGTGTTTCCAGCATAGCTCTT	Library Aplification PCR1
sggeneeSeq12_rev_CAGA	CTCTTTCCCTACACGACGCTCTTCCGATCTNNNNNNTCTGTGTTTCCAGCATAGCTCTT	Library Aplification PCR1
sggeneeSeq13_rev_CAAC	CTCTTTCCCTACACGACGCTCTTCCGATCTNNNNNNGTTGTGTTTCCAGCATAGCTCTT	Library Aplification PCR1
sggeneeSeq14_rev_ATCC	CTCTTTCCCTACACGACGCTCTTCCGATCTNNNNNNGGATTGTTTCCAGCATAGCTCTT	Library Aplification PCR1
sggeneeSeq9_rev_CTCA	CTCTTTCCCTACACGACGCTCTTCCGATCTNNNNNNTGAGTGTTTCCAGCATAGCTCTT	Library Aplification PCR1
sggeneeSeq10_rev_CGAA	CTCTTTCCCTACACGACGCTCTTCCGATCTNNNNNNTTCGTGTTTCCAGCATAGCTCTT	Library Aplification PCR1
sggeneeSeq11_rev_CCTA	CTCTTTCCCTACACGACGCTCTTCCGATCTNNNNNNTAGGTGTTTCCAGCATAGCTCTT	Library Aplification PCR1
Fwd1_P7_read	GTGACTGGAGTTCTAGACGTGTGCTCTTCCGATCTTTGTGGAAAGGACGAAACACCG	Library Aplification PCR1
Fwd2_p7_sgDeepSeq	CAAGCAGAAGACGGCATACGAGATGTGACTGGAGTTCAGACGTG	Library Aplification PCR2
Rev2_p5_sgDeepSeq	AATGATACGGCGACCACCGAGATCTACACTCTTTCCCTACACGACGCT	Library Aplification PCR2

Table S2: List of all non-CRISPR library sgRNAs used in this study. Related to Figure 1, 2,3,6 and Supplementary figure 1,2,3,4,5,6 and 12

Target	Method	sgRNA	Related to
CD13	CRISPRko	GTGCATCCACTGCCATCACG	Figure 1
CD33	CRISPRko	GGAAGGAGCCATTATATCCA	Figure 1
CD13	CRISPRko	GCTCATGTTTGACCGCTCCG	Figure 1
CD33	CRISPRko	GAACCAGTAACCATGAACTG	Figure 1
CD13	CRISPRko	GGGGGAGTTGGCAGATGACC	Figure 1
CD33	CRISPRko	GACGCCAGGAGGAGGGATAA	Figure 1
CD33	CRISPRko	GTCCTGGGGCCCAGGGAGGT	Figure 1
CD13	CRISPRko	GTCTTCTGGAAGTGGGGTGC	Figure 1
CD33	CRISPRi	GCACCTGCCCACAGCA	Figure 1
CD33	CRISPRi	GCAGCGGCATGTCTG	Figure 1
CD13	CRISPRi	GCTCGGAACCCGCCA	Figure 1

CD13	CRISPRi	GACCCGCCAGGGTCCA	Figure 1
CD13	CRISPRi	GGCGGCGGCGCAGCT	Figure 1
CD33	CRISPRi	GCCCCTGCTGTGGGC	Figure 1
CD33	CRISPRi	GGGGCCCAGGGAGGT	Figure 1
CD33	CD33 offtarget RNA-seq sgRNA 1 20mer	CTGCTGCCCCTGCTGTGGGC	Figure 2
CD33	CD33 offtarget RNA-seq sgRNA2 20mer	GTCCTGGGGCCCAGGGAGGT	Figure 2
CD33	CD33 offtarget RNA-seq sgRNA1 15mer	GCCCCTGCTGTGGGC	Figure 2
CD33	CD33 offtarget RNA-seq sgRNA2 15mer	GGGGCCCAGGGAGGT	Figure 2
BUB1	CRISPRi	GCGGCTTCTAGTTTG	Figure 3
BUB1	CRISPRi	TGGCCATGGACACCC	Figure 3
BUB1	CRISPRko	GGTAGCAAAACAGTGTACCC	Figure 3
BUB1	CRISPRko	TGATGAATCTTGGGTCATTG	Figure 3
BUB1	CRISPRi	GGGTATTCGAATCGG	Figure 3
BUB1	CRISPRi	CTCGCCGAACGTTTC	Figure 3
BUB1	CRISPRi	AACGGCCCCGGTGAT	Figure 3
BUB1	CRISPRko	AAGGTTAATCCAGCACGTAT	Figure 3
BUB1 (First exon BM)	CRISPRko	TCCTTCAGTAAGTGTCCGTC	Figure 3
BUB1	CRISPRko	GAAACTCAAAAAATTGATGG	Figure 3
BUB1 (Last Exon BM)	CRISPRko	AGATTAGGGCCCTACGTAAT	Figure 3
ART1	CRISPRi	TTCTGAGCAGCTGTG	Figure 3
ART1	CRISPRko	GGGCCACCCCATGCTCATCG	Figure 3
Ngn2	CRISPRi	GTAGGCGTGTACGGT	Figure 3
Ngn2	CRISPRko	CGCTCACGGGCCGTCTCCCG	Figure 3
SMAD2	CRISPRi	GCACCCACCTCCCGG	Figure 3
SMAD2	CRISPRko	GCGCTTATACTACATAGGTG	Figure 3
TDRD12	CRISPRi	GCCTCCCGCACCCAC	Figure 6
TDRD12	CRISPRko	TGTGGAAGAAGATACATTTG	Figure 6
TDRD12	CRISPRko AVANA	CTATTTAGCAACTACCCAGG	Figure 6
KDM1A	CRISPRi	GGGCAGCGTGAAGCG	Figure 6
KDM1A	CRISPRko	GAATAGCAGAGACTCCGGAG	Figure 6
KDM1A	CRISPRko AVANA	GGAATAGCAGAGACTCCGGA	Figure 6
GFI1B	CRISPRi	GGCCAGTCCCGAGAG	Figure 6
GFI1B	CRISPRko	GCTGACGGAGTGCTCCAGGA	Figure 6
GFI1B	CRISPRko AVANA	CTACAAGCCTAGCTTCTCCT	Figure 6
ZBTB9	CRISPRi	CGTGTGTAACGGCGG	Figure 6

ZBTB9	CRISPRko	AGAAGCAGAGGACTGTACTG	Figure 6
ZBTB9	CRISPRko AVANA	GCATCGGCTTCAATGACACT	Figure 6
DPF1	CRISPRi	GATTTCATTCATTCT	Figure 6
DPF1	CRISPRko	GAGGCACAAAGGAAACACAC	Figure 6
DPF1	CRISPRko AVANA	GTACGTGTAAATCTGTCCCG	Figure 6
TETO-EF1as-mCherry	sgRNA shortening 20mer	TGCGGCCTGTGCAAGCGATG	Supplemental figure 1
TETO-EF1as-mCherry	sgRNA shortening 18mer	CGGCCTGTGCAAGCGATG	Supplemental figure 1
TETO-EF1as-mCherry	sgRNA shortening 16mer	GCCTGTGCAAGCGATG	Supplemental figure 1
TETO-EF1as-mCherry	sgRNA shortening 14mer	CTGTGCAAGCGATG	Supplemental figure 1
Ef1as-mCherry	mCherry offtarget analysis	CGAATTTATGATCAATACCG	Supplemental figure 3
Ef1as-mCherry	mCherry offtarget analysis	CGAATTTATGATCAATACCA	Supplemental figure 3
Ef1as-mCherry	mCherry offtarget analysis	CGAATTTATGATCAATACtA	Supplemental figure 3
Ef1as-mCherry	mCherry offtarget analysis	CGAATTTATGATCACTACCA	Supplemental figure 3
Ef1as-mCherry	mCherry offtarget analysis	CGAATTTACGATCAATACCG	Supplemental figure 3
Ef1as-mCherry	mCherry offtarget analysis	CGAATTTACAATCAATACCG	Supplemental figure 3
Ef1as-mCherry	mCherry offtarget analysis	TTATGATCAATACCG	Supplemental figure 3
Ef1as-mCherry	mCherry offtarget analysis	TTATGATCAATACCa	Supplemental figure 3
Ef1as-mCherry	mCherry offtarget analysis	TTATGATCAATACta	Supplemental figure 3
Ef1as-mCherry	mCherry offtarget analysis	TTATGATCACTACCa	Supplemental figure 3
Ef1as-mCherry	mCherry offtarget analysis	TTACGATCAATACCG	Supplemental figure 3

Ef1as-mCherry	mCherry offtarget analysis	TTACAATCAATACCG	Supplemental figure 3
ZBTB9	CRISPRi	CGTGTGTAACGGCGG	Supplemental figure 12
ZBTB9	CRISPRko	AGAAGCAGAGGACTGTACTG	Supplemental figure 12
ZBTB9	CRISPRko AVANA	GCATCGGCTTCAATGACACT	Supplemental figure 12

Table S3: List of all plasmids generated in this study. Related to star methods

EF1a-ZIM3-Cas9-P2A-GFP-PGK-Blasti	Addgene #239610
EF1a-ZIM3-Cas9-P2A-GFP	Addgene #239603
TRE3G-ZIM3-Cas9-P2A-GFP	Addgene #239605
TRE3G-ZIM3-Cas9-P2A-GFP-PGK-Blasti	Addgene #239604
sgRNA-Dual_filler(hU6_H1)-EF1a-Thy1.1-P2A-Neo	Addgene #239608
sgRNA-Dual_filler(hU6_H1/7SK hybrid)-EF1as_Thy1.1_P2A_Neo	Addgene #239609
