# Supplementary material for: CRISPR GENome and epigenome engineering improves loss-of-function genetic-screening approaches
Source: Cell Rep Methods. 2025 Jun 10;5(6):101078. doi: 10.1016/j.crmeth.2025.101078 (PMC12272254; doi:10.1016/j.crmeth.2025.101078)
Supplement: Document S2. Article plus supplemental information [file mmc6.pdf]

Cell Reports
Methods

Article

CRISPR GENome and epigenome engineering improves loss-of-function genetic-screening approaches

Graphical abstract

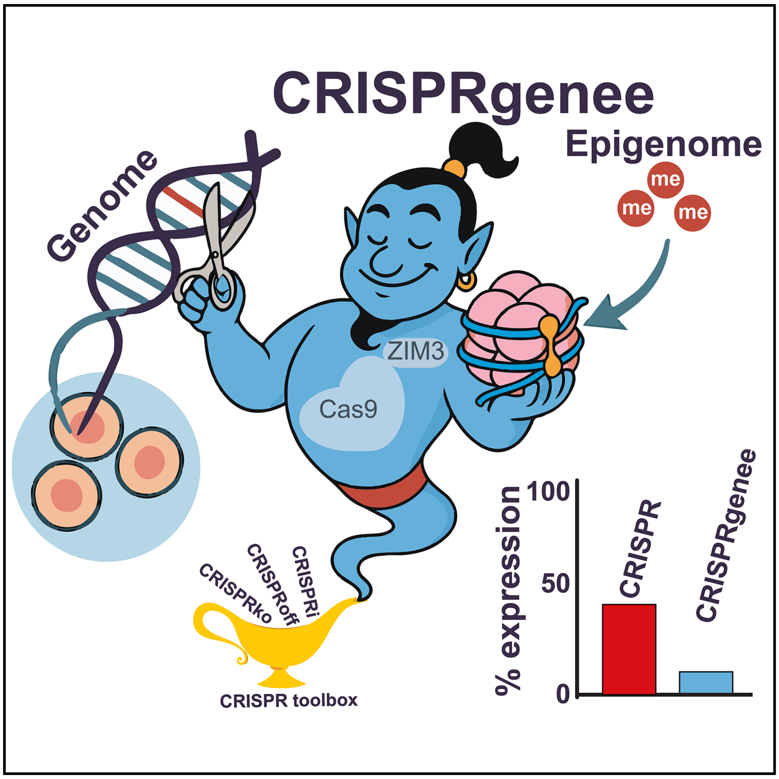

Authors

Jannis Stadager, Chiara Bernardini, Laura Hartmann, ..., Franziska R. Traube, Julian Jude, Philipp Rathert

Correspondence

philipp.rathert@ibc.uni-stuttgart.de

In brief

Stadager et al. present CRISPRgenee, a dual-action gene-editing system that improves the efficiency and reliability of gene loss-of-function studies by combining gene knockout and epigenetic repression in a single step, enabling faster and more reliable identification of genes controlling cell function and disease mechanisms.

Highlights

- CRISPRgenee combines simultaneous gene knockout and epigenetic silencing
- CRISPRgenee significantly improves loss-of-function effects
- CRISPRgenee improves sgRNA heterogeneity improving hit-calling quality
- CRISPRgenee can be used for high-resolution small-library screening

Stadager et al., 2025, Cell Reports Methods *5*, 101078
June 16, 2025 © 2025 The Authors. Published by Elsevier Inc.
https://doi.org/10.1016/j.crmeth.2025.101078

CellPress

Cell Reports Methods

CellPress
OPEN ACCESS

Article

CRISPR GENome and epigenome engineering improves loss-of-function genetic-screening approaches

Jannis Stadager,[1] Chiara Bernardini,[2] Laura Hartmann,[3] Henrik May,[1] Jessica Wiepcke,[1] Monika Kuban,[3] Zeynab Najafova,[4] Steven A. Johnsen,[4] Stefan Legewie,[3] Franziska R. Traube,[2] Julian Jude,[5] and Philipp Rathert[1,6,*]
[1]University Stuttgart, Department of Molecular Biochemistry, Allmandring 31, 70569 Stuttgart, Germany
[2]University Stuttgart, Department of Cellular Biochemistry, Allmandring 31, 70569 Stuttgart, Germany
[3]University Stuttgart, Institute of Biomedical Genetics, Allmandring 31, 70569 Stuttgart, Germany
[4]Robert Bosch Center for Tumor Diseases, Auerbachstraße 112, 70376 Stuttgart, Germany
[5]TWIST Bioscience, 681 Gateway Boulevard, South San Francisco, CA 94080, USA
[6]Lead contact
*Correspondence: philipp.rathert@ibc.uni-stuttgart.de
https://doi.org/10.1016/j.crmeth.2025.101078

MOTIVATION CRISPR technology, including CRISPRko and CRISPRi, enables specific depletion of genes with fewer off-target effects, facilitating precise investigations of gene function. Despite its benefits, CRISPR applications have limitations. Residual active protein expression mediated by in-frame DNA repair or alternative splicing and strong epigenetic regulation along with difficulties in sgRNA design targeting the transcription start site (TSS) hinder the full potential of loss-of-function studies using CRISPRko or CRISPRi. We aimed to achieve robust target gene reduction to improve the reproducibility of the CRISPR technology by integrating the widely used CRISPRko and CRISPRi approaches into a single application.

SUMMARY

CRISPR-Cas9 technology has revolutionized genotype-to-phenotype assignments through large-scale loss-of-function (LOF) screens. However, limitations like editing inefficiencies and unperturbed genes cause significant noise in data collection. To address this, we introduce CRISPR gene and epigenome engineering (CRISPRgenee), which uses two specific single guide RNAs (sgRNAs) to simultaneously repress and cleave the target gene within the same cell, increasing LOF efficiencies and reproducibility. CRISPRgenee outperforms conventional CRISPR knockout (CRISPRko), CRISPR interference (CRISPRi), and CRISPRoff systems in suppressing challenging targets and regulators of cell proliferation. Additionally, it efficiently suppresses modulators of epithelial-to-mesenchymal transition (EMT) and impairs neuronal differentiation in a human induced pluripotent stem cell (iPSC) model. CRISPRgenee exhibits improved depletion efficiency, reduced sgRNA performance variance, and accelerated gene depletion compared to individual CRISPRi or CRISPRko screens, ensuring consistency in phenotypic effects and identifying more significant gene hits. By combining CRISPRko and CRISPRi, CRISPRgenee increases LOF rates without increasing genotoxic stress, facilitating library size reduction for advanced LOF screens.

INTRODUCTION

CRISPR represents the ideal genome engineering system for large-scale forward-genetic-screening approaches to systematically identify new factors involved in normal and pathological processes. Such screens have been employed in many studies,[1] but applications around CRISPR knockout (CRISPRko) can show unpredictable outcomes of non-homologous end joining (NHEJ), resulting in in-frame DNA repair or alternative splicing products[2–9] leading to residual active protein expression. CRISPR interference (CRISPRi), without relying on error-prone DNA double-strand break (DSB) repair, exhibits a more homogeneous response and an improved gene-depletion efficiency without inducing genotoxic stress, which increases with each DSB leading to severe off-target effects in dual-CRISPRko screens.[10] However, studies have shown that the binding position in the promoter region as well as the native epigenetic landscape play an important role in CRISPRi silencing efficiency, with genes harboring multiple TSSs complicating complete repression.[11–14] To compensate for possible inefficient CRISPRi/CRISPRko gene suppression, each gene is generally targeted by 5–20 single guide RNAs (sgRNAs),[13,15,16] which not only increases the overall variability of the investigated phenotype but also drastically elevates the costs of library synthesis as well as sequencing depth. This is

This is an open access article under the CC BY license (http://creativecommons.org/licenses/by/4.0/).

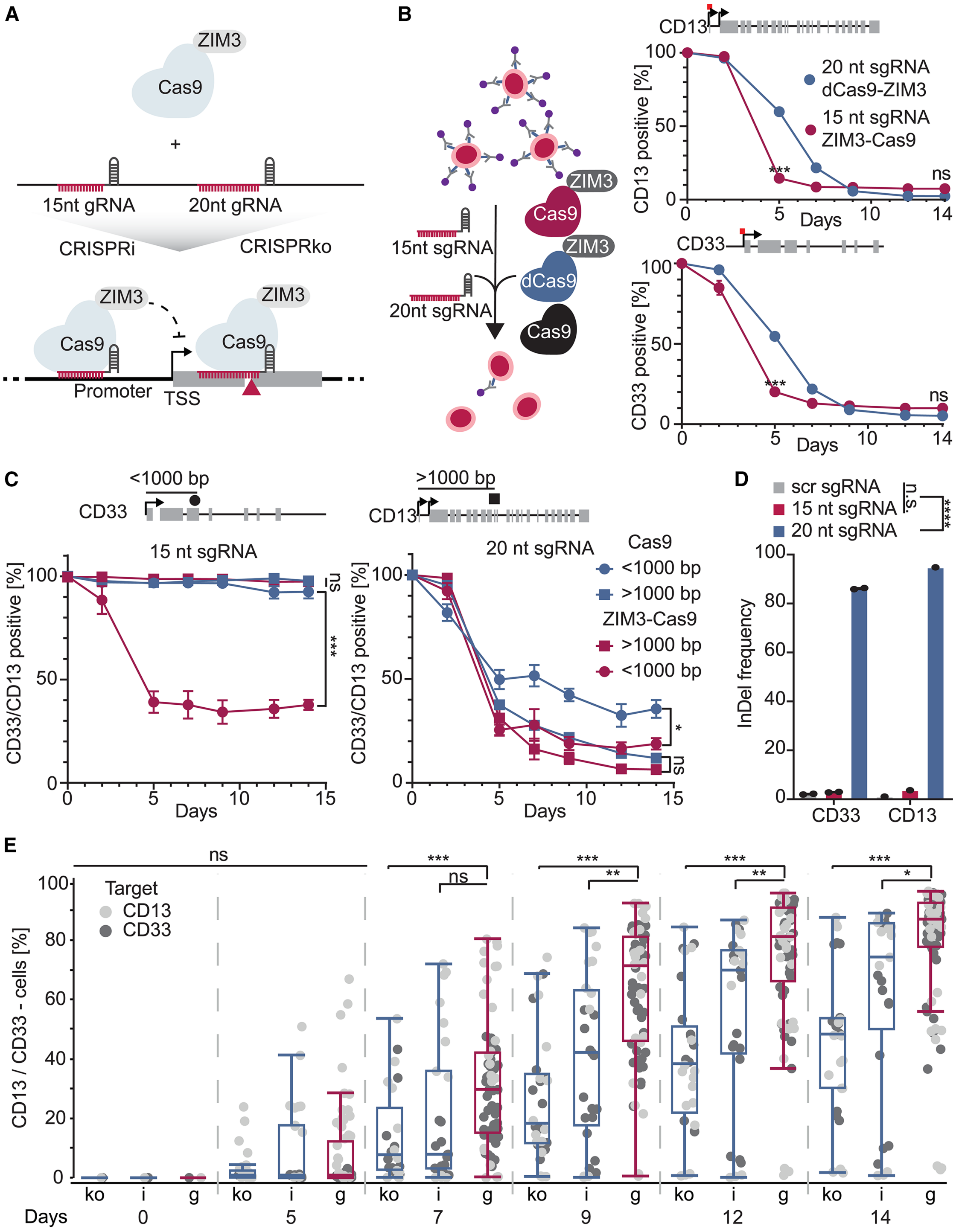

Figure 1. Design and validation of the CRISPRgenee concept to improve standard CRISPR LOF approaches

(A) Schematic overview of the CRISPRgenee setup. An irreversible DNA DSB is introduced by targeting ZIM3-Cas9 to an exon of the gene of interest using a 20-nt guide RNA. This is combined with a simultaneous recruitment of ZIM3-Cas9 to the promoter region of the target gene using a truncated guide RNA inducing a stable repression of gene expression.

(B) Flow-cytometry analysis of the depletion of the non-essential proteins CD13 and CD33 using standard 20-nt-long sgRNAs or truncated 15-nt sgRNAs in TF-1 cells expressing the dCas9-ZIM3 fusion protein. The location of the sgRNA target region is highlighted in red. (n = 3, mean ± SD).

(legend continued on next page)

not ideal for experimental designs in which cell numbers are limited. Nevertheless, such large-scale sgRNA libraries have been deployed to conduct systematic genetic screens to identify essential protein-coding and non-coding genes[13,17–21] and to uncover gene regulatory networks and regulators of disease-associated states[13,22–24] among other things.

To date, several strategies have been employed to optimize current CRISPR LOF approaches to improve on-target and reduce off-target efficiency.[13,15,25–31] Nonetheless, commonly used libraries target each gene with five or more sgRNAs[1,15] with the development of newer highly active LOF libraries reducing the number of sgRNAs to 2–4 sgRNAs per gene.[32–34] The generation of an ultra-compact (1–3 sgRNAs per gene), highly active dual CRISPRko or CRISPRi sgRNA library[27,35] was another recent approach aiming to reduce the constraints imposed by the large size of sgRNA libraries and challenges in generating cell models with consistent CRISPRi-mediated knockdown. However, the high representation of genes targeted only with a single construct complicates the robust identification of screen hits and increases the identification of false-positive hits due to off-target binding.[1] In some cases, the dual-guide approach was not capable of overwriting strong epigenetic marks,[27] and dual-guide libraries targeting only one gene with a single combination are largely ineffective in identifying potential off-target effects. The development of compact, highly active sgRNA libraries would enable CRISPR LOF screens in primary or stem-cell-derived models *in vivo* as well as in pooled CRISPR screens with spatial transcriptome or proteome resolution with high-content readout and other experimental designs where cell numbers are limiting. To address this problem and to improve the reproducibility of the technology in LOF studies, we have developed a CRISPR system, which enables robust target gene reduction through the combination of Cas9 nuclease-mediated DNA cleavage and repressive epigenome editing of the same target gene. Through the fusion of active Cas9 to a powerful transcriptional repressor and the delivery of two sgRNAs from a dual-expression construct, this approach makes use of the optimized sgRNAs developed previously for CRISPRko approaches, which already demonstrate high knockout (KO) effects[31] and simultaneously induces the additional downregulation of residual target transcript expression. The system leads to an increase in the overall LOF of the entire cell population allowing for smaller libraries with much lower sgRNA variation per gene and replicate. The combinatorial CRISPR gene and epigenome engineering (CRISPRgenee) approach paired with reduced-scale sgRNA libraries enables systematic forward genetic screens to interrogate the depletion of genes essential for cell growth and the resulting phenotypes with high efficacy and reproducibility.

RESULTS

Truncated sgRNAs prevent CRISPR nuclease activity while simultaneously silencing gene expression

To improve CRISPR LOF screens, our approach was focused on increasing the phenotypic effect by simultaneous gene and epigenome engineering (CRISPRgenee). Repression of target gene expression while introducing a DNA DSB in a shared exon appeared most effective (Figure 1A). For this purpose, we fused functional Cas9 to the KRAB domain of ZIM3 (ZIM3), which has previously been reported to show superior silencing efficiency among a high number of KRAB domains.[27,29] We confirmed these findings by comparing a fusion of dCas9 with either ZNF10-KRAB or ZIM3-KRAB in an NIH/3T3 reporter cell line expressing mCherry.[36] Promoter targeting revealed stronger silencing by ZIM3-KRAB than ZNF10-KRAB over 14 days (Figure S1A). To simultaneously achieve repression and DNA cleavage, the nuclease activity of Cas9 has to be controlled to maintain the recruitment of Cas9 at the promoter region. It was recently shown that the Cas9 DNA cleavage activity is impaired when sgRNAs are shortened from the 5′-end[37,38] and that targeting of dCas9 can be achieved with <20-nt sgRNAs to repress gene expression.[39] We tested whether truncated sgRNAs could recruit dCas9-ZIM3 and induce an efficient and continuous downregulation of *mCherry* reporter gene expression (Figure S1B). Recruitment of dCas9-ZIM3 to the synthetic promoter for 8 days did not result in a significant difference in reporter gene silencing when comparing the 20-nt sgRNA and various PAM-distal truncated sgRNAs (Figure S1B). We fused ZIM3-KRAB to active Cas9 (ZIM3-Cas9) in a conditional lentiviral expression vector allowing the timed induction of expression via doxycycline (Dox). We transduced either the ZIM3-Cas9 fusion or dCas9-ZIM3 into the erythroleukemia cell line TF-1 with sgRNAs targeting two genes encoding non-essential transmembrane receptor proteins. We targeted the TSS of *CD13* and *CD33* in TF-1 cells using 20- or 15-nt sgRNAs (Figure 1B). Fluorescent antibody staining showed that both ZIM3-Cas9 with a 15-nt sgRNA and dCas9-ZIM3 with a 20-nt sgRNA reduced CD13 and CD33 levels, with faster silencing by truncated sgRNAs at day 5 but comparable efficiency by day 14 (Figure 1B). As a next step, we wanted to investigate the DNA cleavage ability of ZIM3-Cas9 using 15-nt sgRNAs as well as 20-nt sgRNAs and compare this effect to Cas9 alone. ZIM3-Cas9 or Cas9 expression was induced through the addition of Dox for 14 days with both 20-nt sgRNAs targeting either *CD13* (>1,000 bp distance to the

(C) Flow-cytometry analysis of the depletion of CD13 and CD33 using standard 20-nt sgRNAs or truncated 15-nt sgRNAs in TF-1 cells expressing either Cas9 or the ZIM3-Cas9 fusion protein. The location of the sgRNA target region is highlighted in black, indicating whether the sgRNA is within 1,000 bp of the nearest transcription start site (TSS) ($n = 3$, mean ± SEM).

(D) Indel frequency of the *CD13* and *CD33* locus in ZIM3-Cas9+ TF-1 cells treated with control, 15-nt, or 20-nt sgRNAs.

(E) Time-resolved quantification of CD13 and CD33 negative TF-1 cells expressing the indicated sgRNAs after induction of ZIM3-Cas9. A total of eight CRISPRko (ko) and seven CRISPRi (i) sgRNA designs were combined in 22 CRISPRgenee (g) constructs for comparison. Data are displayed as a single datapoint for each sgRNA or sgRNA combination and replicate summarized in a boxplot ($n = 3$, mean, box, and whiskers minimum to maximum); *$p \leq 0.05$, **$p \leq 0.01$, ***$p \leq 0.001$; n.s., non-significant.

Significance was determined using a one-way ANOVA for (D) and a two-way ANOVA for (B), (C), and (E).

See also Figures S1 and S2.

TSS) or *CD33* (<1,000 bp distance to the TSS) resulting in a strong reduction of CD13 and CD33 in ZIM3-Cas9 as well as in Cas9-expressing cells (Figure 1C). Both ZIM3-Cas9 as well as wild-type (WT) Cas9 had comparable efficiencies with the 20-nt sgRNA guide, whereas the 15-nt analog targeting the same genomic region within the gene body of *CD13* failed to induce any loss of functional protein. However, we observed that the 15-nt sgRNA that targets the gene body of *CD33* did induce a significant protein reduction in cells expressing ZIM3-Cas9 but not with WT Cas9 (Figure 1C). After Dox withdrawal, we tracked *CD13*/*CD33* expression over 64 days (Figure S1C). No recovery of *CD13* expression was observed in cells where ZIM3-Cas9 or Cas9 was targeted with the 20-nt sgRNA hinting at an irreversible DNA DSB induced by ZIM3-Cas9 or Cas9 cleavage (Figure S1C). The reduction of CD33 observed for the truncated sgRNA when using ZIM3-Cas9 was not stable after the removal of Dox, hinting at a reversible CRISPRi effect on the 800-bp distant promoter region. In contrast, the identical 20-nt-long sgRNA demonstrated an irreversible reduction of CD33, observed after removal of Dox in both ZIM3-Cas9 and Cas9 cells (Figure S1D). The striking difference observed between the two targets when using the truncated sgRNA could be explained by the distance between the targeted region and the TSS. For *CD33*, the targeting region was within 1,000 bp of the TSS, whereas, for *CD13*, the targeting region was outside this range (Figure 1C) suggesting that, in the case of *CD33*, binding of ZIM3-Cas9 was able to silence *CD33* expression, which was recovered after Dox removal (Figure S1D). To confirm that CD13 and CD33 loss with 20-nt sgRNAs resulted from DNA cleavage, we performed a mismatch-cleavage assay, detecting cleavage products only with 20-nt guides, not with 15-nt sgRNAs. (Figure S1D). Additionally, we performed amplicon sequencing of the Cas9 cleavage site to quantify the amount of DSB breaks observed for the 15- and 20-nt guides (Figure 1D) and did not detect a significantly higher insertion or deletion (indel) frequency when using the 15-nt guide compared to the control, whereas, when using the 20-nt guide, a significant increase in indel frequency was observed (Table S4). This suggested that, in the case of CD33, for which ZIM3-Cas9 had a significantly stronger silencing effect compared to Cas9 when using the 20-nt sgRNA, we observed the simultaneous effect of genome editing and transcriptional interference, which was not anticipated, demonstrating that our envisioned approach could improve LOF approaches by simultaneous gene editing and epigenome interference.

CRISPRgenee significantly accelerates and increases CRISPR-mediated gene depletion

We next tested whether CRISPRgenee improves the time and efficiency of gene silencing compared to CRISPRi and CRISPRko. To this end, we performed a time-resolved reduction of CD13 and CD33 protein levels in TF-1 cells using the same inducible ZIM3-Cas9 construct. We used sgRNAs targeting promoters or gene bodies and tracked CD13/CD33 protein levels by flow cytometry (Figure 1E). In order to investigate a potential combinatorial effect when using CRISPRgenee, the *CD13* and *CD33* sgRNAs were chosen based on their predicted *in silico* depletion efficiencies[40] (low, medium, and high) and used in combination (CRISPRgenee) compared to CRISPRi or CRISPRko alone. Given that 15-nt sgRNAs silence as effectively but faster than 20-nt sgRNAs (Figure 1B), we used them as CRISPRi controls to isolate CRISPRgenee's benefits to the combination of gene and epigenome editing. Initial CRISPRgenee combinations of sgRNAs with varying predicted efficiency significantly enhanced *CD33* suppression (Figure S1E).

Building on these initial results, we tested a broader set of CRISPRko and CRISPRi sgRNAs (with varied *in silico* prediction scores)[40] and their CRISPRgenee combinations on *CD13*/*CD33* expression over 14 days (Figure 1E). Overall, CRISPRgenee showed significantly stronger CD13/CD33 suppression after 7 days, consolidating over time. (Figure 1E). Strikingly, it also reduced variance in sgRNA performance, yielding more consistent silencing than CRISPRi/CRISPRko (Figure 1E). To assess whether the increased depletion efficiencies follow a combinatorial effect, we analyzed the amount of CD13/CD33 negative cells at day 9 and day 14 for all CRISPRi sgRNAs with their respective CRISPRko sgRNA combinations. Interestingly, we were not able to identify a clear trend for the combinatorial effect (Figure S2A). While combining two non-functional sgRNAs did not boost depletion, pairing a weak with a stronger sgRNA improved silencing. Thus, CRISPRgenee accelerates and standardizes gene depletion compared to single-guide LOF approaches (Figures 1E and S2A). We confirmed the combinatorial benefit by comparison to conventional dual-sgRNA setups and observed that CRISPRgenee led to faster, more efficient silencing (Figure S2B). Overall, these results demonstrate that the combination of CRISPRko and CRISPRi sgRNAs within the CRISPRgenee system leads to a beneficial LOF effect.

Truncated and normal-length sgRNAs exhibit similar off-target effects

We anticipated broader off-target effects from the 15-nt sgRNA due to its role in transcriptional repression, while 20-nt guides were expected to behave like standard Cas9 guides. Although recent literature highlights that only the first five bases in the seed region influence on- and off-target activity of dCas9,[41–43] and therefore the reduction of 5 bp in the PAM-distal part should not influence binding, we aimed to determine the transcriptional effects of two sgRNAs targeting *CD33*. To this end, we selected sgRNAs predicted to harbor several perfect off-targets in the genome. We transduced dCas9-ZIM3 TF-1 cells with 15- or 20-nt sgRNAs and analyzed transcriptome changes after 14 days by RNA sequencing (RNA-seq) (Table S5). Interestingly, the principal-component analysis (PCA) showed that truncated and full-length guides clustered closely, indicating minimal differences (Figure 2A). Log fold change (LFC) values (sgRNA to control) showed high Spearman correlation between matched 15- and 20-nt sgRNAs, suggesting the final five bases to have minimal impact on off-target effects (Figure 2B). Direct comparison revealed only minor differences in gene expression between 15- and 20-nt guides (Figure 2C). We predicted all off-targets with up to three mismatches (MMs) *in silico* and filtered for all off-targets in the range of −1,000 to 1,000 bp to the nearest TSS. No significant differences in LFCs were observed between sgRNA lengths, regardless of mismatch number (Figures 2D and S3A). Exemplarily, we identified a subset of off-targets with a

CellPress
OPEN ACCESS

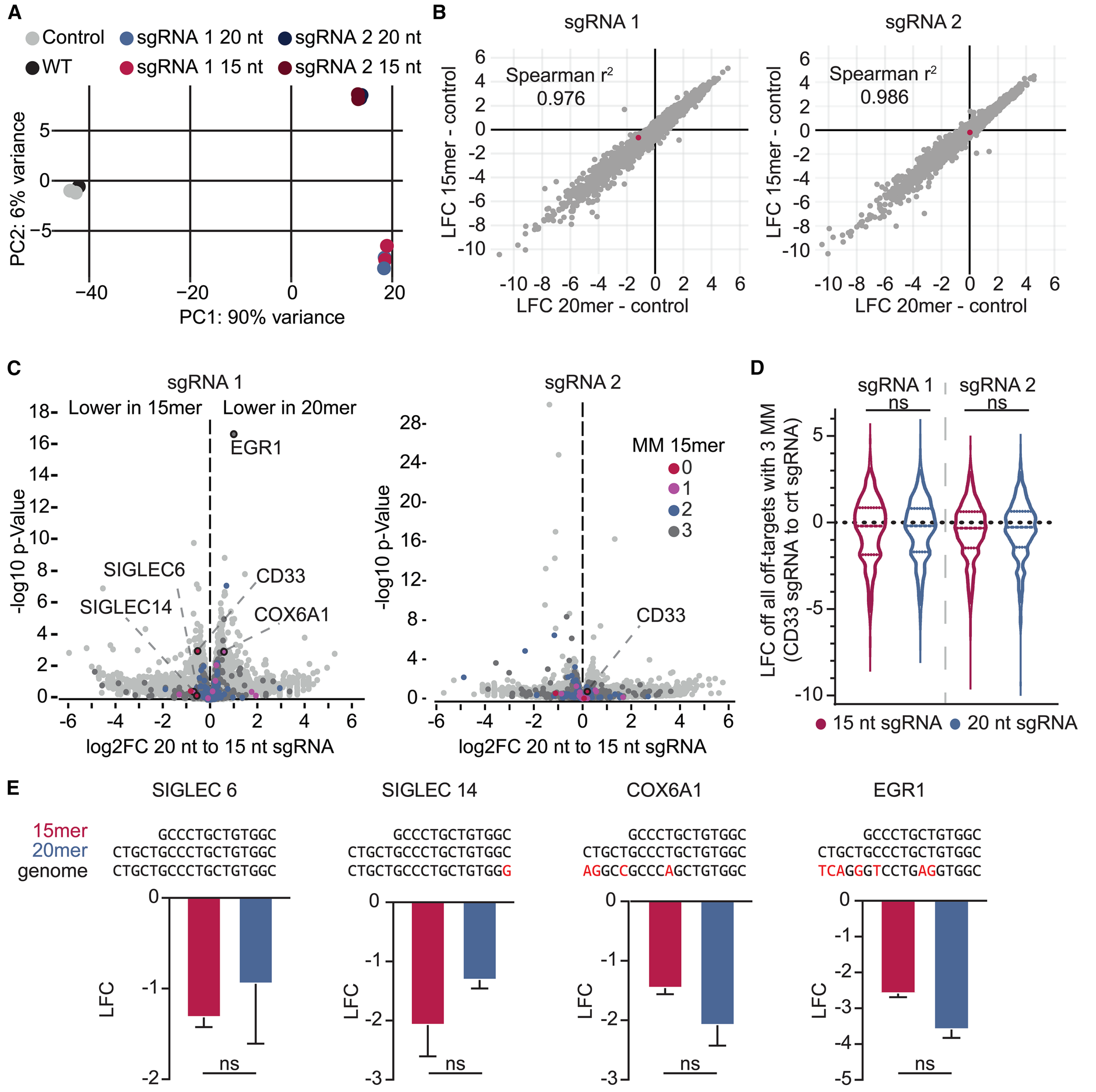

Figure 2. Off-target analysis of 15- and 20-nt-long sgRNAs does not reveal significant differences

(A) Principal-component analysis (PCA) of the RNA-seq data for two full-length (shades of blue) and truncated (shades of red) *CD33*-targeting sgRNAs, non-targeting control (black), and WT (gray).

(B) Correlation of the log2 fold change of sgRNA to control for each transcript between the truncated and full-length sgRNA was assessed using Spearman correlation analysis.

(C) Log2 fold change of 15- and 20-nt sgRNAs targeting the TSS of *CD33*. Off-targets toward TSSs of other genes were predicted *in silico* for up to three mismatches for the 15-nt sgRNA and are indicated by color.

(D) Log2 fold change of the 15- or 20-nt *CD33*-targeting sgRNA and the sgRNA control for all potential off-targets identified *in silico*.

(E) Log2 fold change of identified off-targets for the 15-nt sgRNA calculated for both sgRNA variations with the DNA base mismatches indicated in red.

See also Figure S3.

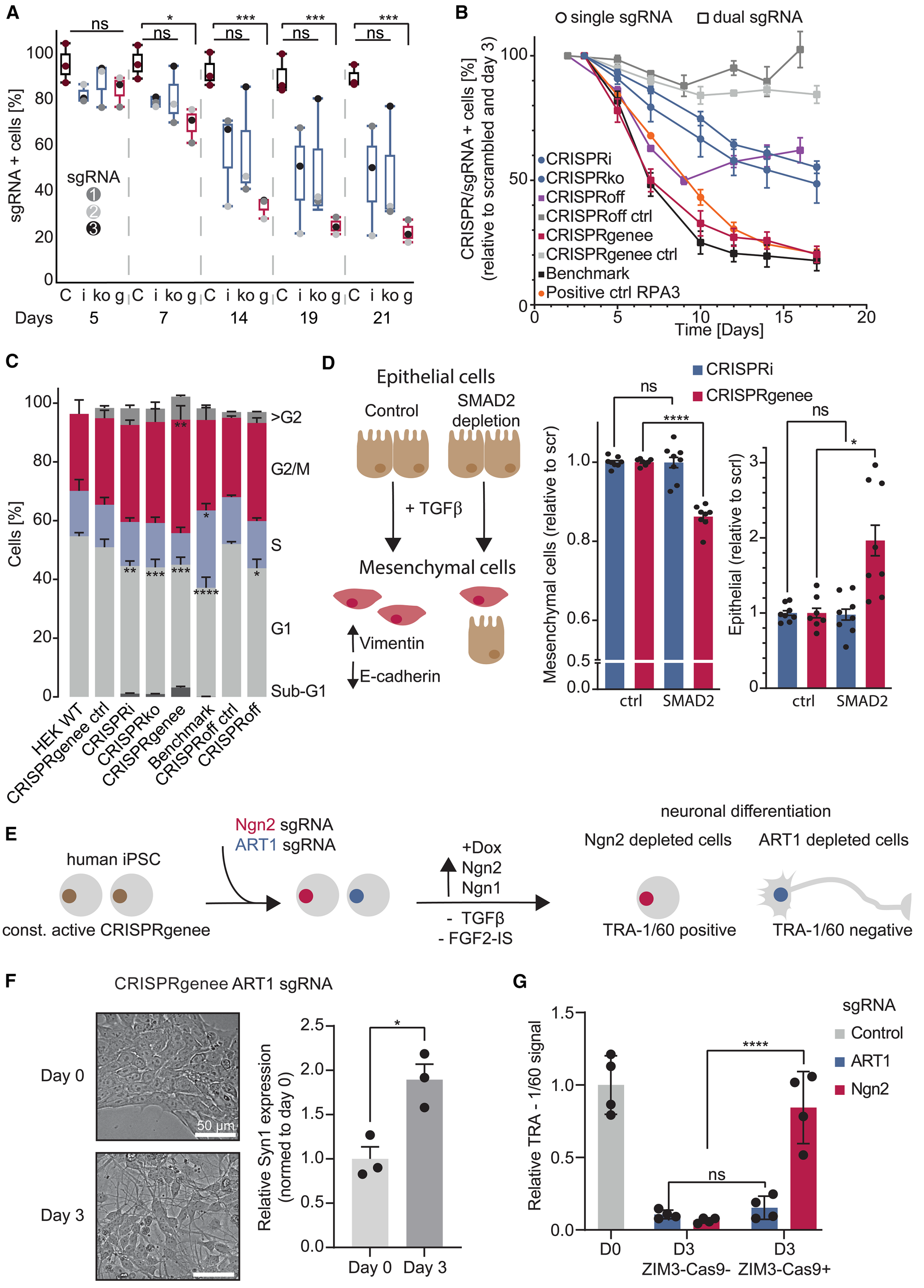

(legend on next page)

variety of MMs to the sgRNA, demonstrating similar effects of the 15-nt sgRNA and 20-nt sgRNA (Figure 2E). Overall, recruitment of dCas9 to the DNA seems to tolerate MMs more than expected, to the extent that there are no significant differences when using a 15-nt sgRNA with two MMs to the target region or the corresponding 20-nt sgRNA with four or five MMs to the target region (Figure 2D).

We validated this in the *mCherry* reporter system[36] using matched sgRNAs with up to two MMs (Figure S3B). Only two consecutive mismatches in the seed region significantly impacted the silencing of *mCherry*, whereas guide length had no effect (Figures S3B and S3C). These data imply that DNA cleavage-based off-target models for Cas9 do not reliably predict dCas9 binding activity, aligning with recent studies.[41–43]

Assessment of phenotypic consequences following CRISPRgenee-mediated BUB1 depletion

Given CRISPRgenee's improved depletion of CD13/CD33, we hypothesized it would cause stronger phenotypic effects than conventional methods. To evaluate this hypothesis, we applied CRISPRgenee to BUB1, a key spindle assembly checkpoint (SAC) kinase essential for cell-cycle progression and proliferation.[44] However, the essentiality of BUB1 was under debate[45–48] since KO cells often retain partial expression via alternative splicing.[44,47–49] Only the removal of the remaining *BUB1* mRNA by RNAi or the complete removal of the *BUB1* gene substantially affected the cell cycle.[50] Targeting distinct domains of *BUB1* did not result in a loss of functionality, with alternative splicing rendering the generated indels useless.[49,51] Only the full removal of the gene by CRISPR was successful to result in a complete loss of BUB1 in haploid cells, making it an ideal target to benchmark CRISPRgenee.[51,52]

Standard LOF screening approaches assess the essentiality of a specific gene by determining the phenotypic effects of 5–20 sgRNAs. To determine whether the increased efficiency observed on the level of non-essential proteins (Figure 1E) can be translated to a potential multiplexed LOF screening approach we aimed to assess whether targeting *BUB1* would generate comparable phenotypic results, which was shown to express alternative variants after CRISPR-Cas knockout.[44,49] To this end, we selected the top-five predicted sgRNAs for CRISPRko and CRISPRi[40] targeting *BUB1* and sequentially combined the top-three sgRNAs to be used in all possible combinations in the CRISPRgenee system (Figure S4A). TF-1 cells expressing conditional ZIM3-Cas9 were transduced with the respective *BUB1* sgRNAs, and the fraction of sgRNA-positive cells was monitored for 21 days. Despite selecting top-ranked sgRNAs, most CRISPRi/CRISPRko guides showed weak and variable effects (Figure S4A). We integrated the mean effect of the top-three sgRNAs for CRISPRi, CRISPRko, and the respective CRISPRgenee combinations to simulate a screening setup in which three sgRNAs per gene would be used (Figure 3A). CRISPRgenee combinations caused early and strong depletion, with effects growing over time. Overall, the CRISPRgenee sgRNA combinations not only led to a stronger phenotypic effect after a shorter time frame but also displayed less variance, resulting in better statistical significance compared to the three sgRNAs used for CRISPRi and CRISPRko (Figure 3A), suggesting that CRISPRgenee achieves significant effects at earlier time points. With the top-three predicted CRISPRi sgRNAs targeting *BUB1* producing a high heterogeneity in response to BUB1 depletion, we wanted to assess whether transcriptional silencing of *BUB1* might be inefficient due to the essentiality of this gene. Therefore, we used the recently published CRISPRoff technology[30] in combination with three dual-sgRNA plasmids targeting the TSS of *BUB1*. CRISPRoff produced delayed and inconsistent effects, with significant depletion only by day 19 (Figures S4B and S4C). To investigate the biological impact of BUB1 depletion on cell-cycle progression, we selected *BUB1* sgRNA combinations, which displayed a faster negative proliferative effect and investigated these in HEK293 cells expressing conditional ZIM3-Cas9 or CRISPRoff (Figure 3B). Additionally, we compared CRISPRgenee to conventional Cas9 using a dual-sgRNA strategy simultaneously targeting the first and last exon of *BUB1*, which was previously reported to achieve a full removal of the *BUB1* gene in HAP1 cells but not in any other cell line.[51] CRISPRgenee matched the benchmark dual-cutting Cas9 strategy in reducing proliferation (Figure 3B). The BUB1 depletion using CRISPRoff (dual sgRNA) resulted in a faster phenotypic effect compared to CRISPRi but reached similar endpoint values and was not able to increase the negative proliferative effect further (Figure 3B). CRISPRoff led to toxicity even with scrambled (scr)

Figure 3. CRISPRgenee outperforms standard CRISPR LOF methods and validates the essential role of BUB1 in cell-cycle progression

(A) Competitive proliferation assays of TF-1 cells expressing the indicated sgRNAs (gray scale) targeting *BUB1*. The data show the mean relative fraction of GFP+/sgRNA+ cells for three CRISPRi (i) and CRISPRko (ko) sgRNAs, and the respective CRISPRgenee (g) sgRNA combination, relative to the initial measurement over a 21-day period ($n = 3$, mean ± SD).

(B) Competitive proliferation assays of HEK293 cells expressing ZIM3-Cas9 or the CRISPRoff construct and the indicated sgRNAs (dual- or single-sgRNA setup) targeting *BUB1* to validate the improved CRISPRgenee effect observed in TF-1 cells ($n = 3$, mean ± SEM).

(C) Inferred distribution of cell-cycle phases of HEK cells harvested at day 5 of BUB1 depletion as indicated in (B). Percentages in each phase of the cell cycle were automatically assigned using FlowJo ($n = 3$, mean ± SEM).

(D) EMT was induced in MCF10A cells expressing either dCas9-ZIM3 or ZIM3-Cas9 and scr (ctrl) or *SMAD2*-targeting sgRNAs using TGFβ. The fold change of cells detected in the epithelial and mesenchymal population was calculated in SMAD2-depleted cells relative to the control ($n = 3$, mean ± SEM).

(E) Schematic overview of the experimental setup used to validate the tolerability of CRISPRgenee in iPSCs.

(F) Differentiation of iPSCs expressing CRISPRgenee and an *ART1*-targeting sgRNA was monitored for 3 days by assessing cell morphology and expression of *Syn1*.

(G) Relative TRA-1/60 signal (a marker for pluripotency) of ZIM3-Cas9-positive and ZIM3-Cas9-negative Dox-stimulated iPSCs either expressing an sgRNA targeting *ART1* or the transgene *Ngn2* responsible for neuronal differentiation.

*$p \leq 0.05$, **$p \leq 0.01$, ***$p \leq 0.001$; n.s., non-significant. Significance was determined using a two-way ANOVA for (A)–(C) and a one-way ANOVA for (D).
See also Figures S4 and S5.

guides, likely due to nonspecific silencing by DNMT3A-3L, consistent with prior reports (Figures S4D and S4E).[28,50,53,54] CRISPRgenee reduced *BUB1* mRNA to 30%, compared to ∼50% for CRISPRi/CRISPRoff Figure S4E). To determine whether the negative proliferative effect observed upon *BUB1* suppression is a result of its essential function in cell-cycle regulation, which will result in DNA replication stress and subsequent apoptosis, we performed DNA content analysis to assess cell-cycle effects of BUB1 depletion (Figure S4F). Both CRISPRgenee and CRISPRoff in combination with an sgRNA targeting a non-essential gene did not result in significant differences in cell-cycle distribution compared to the HEK WT cells, whereas a significantly decreased G1 cell population was observed for all BUB1 depletion samples (CRISPRi, CRISPRko, CRISPRgenee, benchmark, and CRISPRoff) (Figure 3C). However, only CRISPRgenee and the benchmark resulted in a significant difference in other cell-cycle stages compared to HEK WT cells. The benchmark sgRNAs demonstrated a significant accumulation of cells in the S phase, whereas CRISPRgenee resulted in a significantly higher cell population in the G2/M phase of the cell cycle, which would be in conjunction with the proposed function of BUB1 in the M phase of mitosis.[44] Only CRISPRgenee increased the sub-G1 population, indicating apoptosis, which was absent in other samples (Figure 3C). These findings imply that the observed phenotype does not stem from higher genome toxicity but, rather, from the impaired cell cycle attributed to the improved reduction of BUB1.

CRISPRgenee extends to non-tumorigenic and stem cell contexts

Next, we wanted to assess whether CRISPRgenee can be applied to cells sensitive to DNA damage and epigenetic silencing such as primary cells or stem cells. To test whether we could effectively perturb epithelial-to-mesenchymal transition (EMT) induced by transforming growth factor (TGF)-β, we transduced the non-tumorigenic epithelial MCF10A cells with dCas9-ZIM3 or ZIM3-Cas9 and observed normal growth behavior for either construct. We then transduced these cells with sgRNAs targeting *SMAD2*, a known mediator of TGF-β signaling as well as an scr sgRNA control. After 1 week, we induced EMT using TGF-β, stained for E-cadherin and vimentin and validated the loss of *SMAD2* via qPCR (markers of EMT) (Figures 3D, S5A, and S5B). Cells expressing the scr sgRNA control showed reduced E-cadherin and increased vimentin, while SMAD2-depleted cells showed significantly reduced EMT using CRISPRgenee but not CRISPRi (Figure 3D). Importantly, the cells that did not react to TGF-β-induced EMT due to downregulation of *SMAD2* by CRISPRgenee did not exhibit negative effects on viability due to genotoxic stress but instead remained in the epithelial cell population (Figure 3D).

We further validated CRISPRgenee in human induced pluripotent stem cells (iPSCs) engineered for neuronal differentiation upon Dox induction via mouse transgenes *Neurogenin-2* (*Ngn2*) and *Neurogenin-1* (*Ngn1*) (Figure 3E).[55] We designed CRISPRgenee sgRNA combinations, one targeting a non-essential gene expressed in neuronal cells (*ART1*) and another sgRNA combination targeting the transgene *Ngn2*, validated the loss of *ART1* via qPCR (Figure S5C), and induced neuronal differentiation. iPSCs expressing the sgRNA targeting *ART1* showed the anticipated morphological changes after 3 days (Figure 3F) and upregulation of *SYN1* (Figure 3F), a gene that is activated in neuronal differentiation.[55] The cells were harvested and stained for a cell-surface pluripotency marker (TRA-1/60), which is reduced after differentiation to neuronal cells.[55] We measured a significant loss of TRA-1/60 compared to the uninduced WT, without finding any significant differences between the ZIM3-Cas9-negative and ZIM3-Cas9-positive cells expressing the sgRNA targeting *ART1* (Figure 3G). This indicates that the iPSCs maintain normal phenotypic behavior and signaling, unaffected by potential DNA damage stress from CRISPRgenee expression. However, targeting *Ngn2* significantly impaired differentiation, as shown by higher TRA-1/60 levels in ZIM3-Cas9-positive cells compared to negative controls (Figure 3G), confirming that CRISPRgenee effectively disrupts cellular differentiation.

CRISPRgenee improves LOF screen performance

Having demonstrated that CRISPRgenee improves depletion efficiency and phenotypic consistency with reduced variance (Figures 1E, 3A, and 3B), we hypothesized that its enhanced suppression and lower variance would enable faster, more robust hit calling at lower library coverage. We therefore decided to test the CRISPRgenee system using a multiplexed LOF phenotypic screening approach and collaborated with Twist Bioscience to design and clone dual-sgRNA oligos comprising a 15-nt sgRNA, tracr, promoter, and 20-nt sgRNA (Figure S6A). To synthesize dual sgRNAs on a <300-nt oligo, we used a minimal hybrid H1 promoter substituting all core elements except the Staf domain with 7SK promoter elements (minH1/7SK), which reportedly only shows Pol III activity.[56] Since the H1/7SK promoter substitutes the H1 promoter used for the CRISPRko sgRNA, we tested previous CRISPRko sgRNAs targeting *CD33* observing no significant differences in *CD33* depletion (Figure S6B). Notably, replacing H1 with minH1/7SK significantly enhanced hU6-driven truncated CRISPRi sgRNA activity, yielding faster CD13 reduction (Figure S6B). Furthermore, silencing effects using CRISPRi can be seen faster compared to when using CRISPRko, which might also explain why a difference could be detected for the CRISPRi but not the CRISPRko. Using the minH1/7SK promoter, we synthesized and cloned a CRISPRgenee oligonucleotide pool designed to target 1137 genes (including 10 internal controls) involved in chromatin regulation.[57,58] For each gene, three CRISPRgenee combinations were designed by pairing the top-three CRISPRi (15 nt) and CRISPRko sgRNAs selected using the latest prediction algorithms.[31,40] For genes with multiple annotated transcription start sites (TSSs) with a distance of >1,000 bp from each other, the same CRISPRko sgRNAs were combined with different 15-nt sgRNAs targeting each TSS (Figure S6C). The initial 270-nt oligo pool was synthesized and quality control (QC) checked by deep sequencing post cloning into the screening vector, and the overall distribution was calculated (Figure S6D). The CRISPRgenee library harboring 3686 sgRNAs in total (Table S6) was transduced in triplicate into three independent conditional ZIM3-Cas9 TF-1

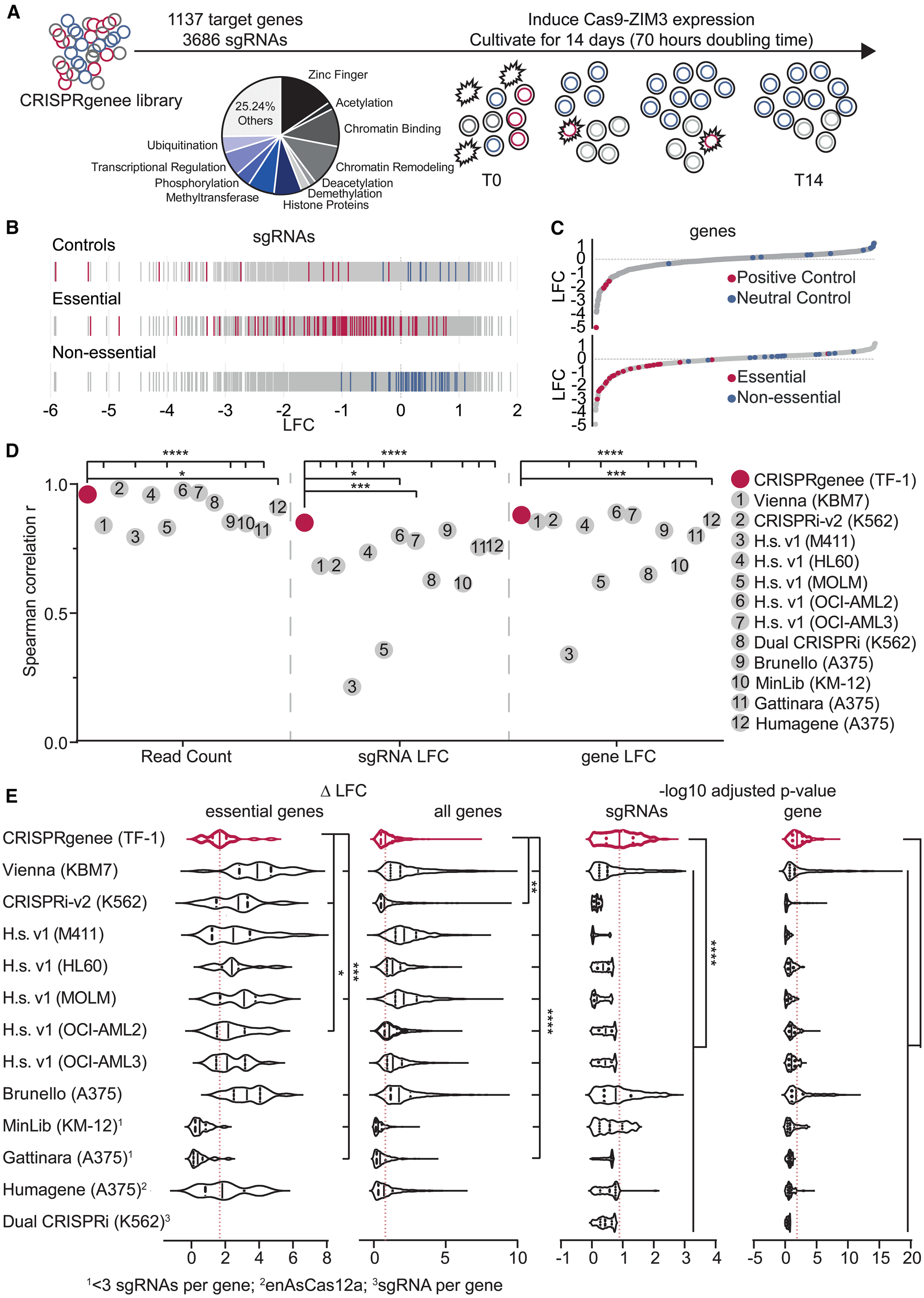

(legend on next page)

cell clones. After selection with neomycin (NEO) for 7 days, ZIM3-Cas9 expression was induced by the addition of Dox (Figure 4A). Based on encouraging BUB1 suppression results (Figures 3A and 3B), showing earlier, more reproducible negative proliferative effects with CRISPRgenee, cells were passaged only seven times (14 days, 70-h doubling time).[27] The initial chimera rate in the oligo pool was 9.4%, which increased after library preparation from gDNA ranging from 14% to 20% per single-cell clone (Figure S7A), while library preparation of the oligo pool produced a 34.6%–38.0% chimera rate, likely due to unfavorable PCR conditions optimized for sgRNA amplification from gDNA. We filtered sequenced reads where the 15-nt and 20-nt sgRNAs matched the designed oligonucleotides. Overall, the CRISPRgenee screen showed remarkable reproducibility across replicates (Figures S8 and S9), and we assessed sgRNA and gene performance using the CRISPRBetaBinomial (CB2) algorithm.[59] Neutral-control sgRNAs targeted non-essential *CD13*/*CD33* genes, whereas positive controls targeted *RPA3*, *MYC*, and *CSF2RA*, a receptor subunit of the granulocyte colony-stimulating factor (G-CSF), essential for TF-1 proliferation (Figure S7B). As expected, neutral-control sgRNAs did not show a strong effect on cell proliferation in contrast to positive sgRNAs (Figure 4B). Next, we investigated the effect of individual sgRNAs targeting a set of established common essential and non-essential genes.[60] Targeting essential genes led to negative LFC values contrary to sgRNAs targeting non-essential genes, which clustered around LFC values from 0 to 1. This effect was also visible on the gene LFC level (Figures 4C and S7C). We calculated the receiver operating characteristic (ROC) area under the curve (AUC) using all sgRNAs targeting gold-standard essential/non-essential genes (Figure S10A), demonstrating that CRISPRgenee, despite fewer sgRNAs per gene and passages, rivals many high-end published CRISPR screens (Table S7).[13,15,21,27,31–33,35] Spearman correlations between replicates for read counts, sgRNA, and gene LFC showed high reproducibility (Figures 4D, S8, and S9). Our previous data suggested that CRISPRgenee reduces the overall noise within phenotypic effects among sgRNAs targeting the same gene (Figures 1 and 3). Therefore, we compared the ΔLFC (maximum LFC minus minimum LFC) between sgRNAs targeting the same gene to the identical genes in recently published LOF screens.[13,15,21,27,31–33,35] Among essential gene targets, CRISPRgenee showed significantly lower ΔLFC across sgRNAs when three or more sgRNAs per gene were used (Figure 4E), which was also the case when filtering for essential genes, indicating an overall more consistent sgRNA performance (Figure 4E). A similar trend was observed when we investigated the variance of the LFC for all sgRNAs targeting the same gene (Figure S10B). To test whether reduced variance improved hit calling, we performed the same analysis on –log10 adjusted *p* values (false discovery rate [FDR]) for sgRNAs in published screens filtered for CRISPRgenee chromatin library genes, showcasing that sgRNAs exhibited a much higher reproducibility in significant phenotypic effects in the CRISPRgenee screen compared to the other analyzed public screens (Figures 4E and S11). Interestingly, high sgRNA-level significance in some published LOF screens did not transfer to the gene level, suggesting that sgRNA heterogeneity raises variance and reduces gene-level significance. With the CRISPRgenee approach, this loss in significance was not observed (Figure 4E), resulting in more consistent significant gene hits with the CRISPRgenee screening approach.

CRISPRgenee generates improved dropout effects through a combinatorial effect of CRISPRi and CRISPRko

To test whether CRISPRgenee's improved performance (Figure 4) resulted from combining CRISPRko and CRISPRi sgRNAs on one construct, as suggested by earlier experiments (Figures 1 and 3), we compared dropout effects in genes with two targeted TSSs. We identified genes showing strong LFC differences between two TSSs (Figure S12A), independent of their distance (Figure S12B). Additionally, we did not identify any correlation between LFC and distance of the targeting KO sgRNA to the TSS for essential genes (Figure S12C), suggesting no epigenetic silencing over long distances through retention of ZIM3-Cas9 after DNA cleavage. We further classified TSSs as functional (F-TSS, where CRISPRi enhances LOF) or non-functional (NF-TSS, no CRISPRi effect), revealing a significant gene-depletion difference when targeting different TSSs (Figures 5A and S12D). For instance, CRISPRgenee sgRNAs targeting the myeloid selective genes *ADAR* and *ERG* (DepMap)[61,62] resulted in a relatively moderate negative LFC when sgRNAs were directed against the NF-TSS. However, combining the same CRISPRko sgRNAs with sgRNAs targeting the F-TSS led to a drastic LFC decrease for all three CRISPRko sgRNAs (Figure 5B). Since the CRISPRi sgRNAs used to assemble the

Figure 4. CRISPRgenee outperforms published LOF dropout screens with improved sgRNA consistency

(A) Schematic overview of the screening setup used for the CRISPRgenee screen. A library composed of 3,686 sgRNAs targeting 1,137 chromatin-related genes was virally transduced into TF-1 erythroleukemia cells expressing ZIM3-Cas9. After antibiotic selection, cells were treated with Dox for 14 days.

(B) Performance of individual sgRNAs. The log fold change (LFC) of all sgRNAs targeting internal positive (red) and neutral controls (blue) as well as sgRNAs targeting known essential (red) and non-essential genes (blue) is depicted. Data are averaged across three individual replicates.

(C) Scatterplot depicting all genes ranked by the average LFC of all sgRNAs per gene across all three replicates. Internal positive (red) and neutral (blue) control genes (top), as well as essential (red) and non-essential genes (blue) are highlighted (bottom).

(D) Spearman correlation r was calculated for the replicates of the CRISPRgenee screen and replicates of published screen datasets on read count, sgRNA LFC, and gene LFC level.

(E) Violin plots comparing the CRISPRgenee system with a set of published CRISPR screening approaches. Left: comparison of the ΔLFC (maximum LFC − minimum LFC) of sgRNAs targeting the same gene depicted for essential genes and all genes investigated in the screen. Right: comparison of the −log 10 adjusted *p*-value distribution at the sgRNA and gene level. The black vertical lines depict the median for each screen and the red dashed line is the median of the CRISPRgenee screen (*$p \leq 0.05$, ***$p \leq 0.001$, ****$p \leq 0.0001$; one-way ANOVA with a Dunnett *post hoc* test).

See also Figures S6–S11.

CellPress
OPEN ACCESS

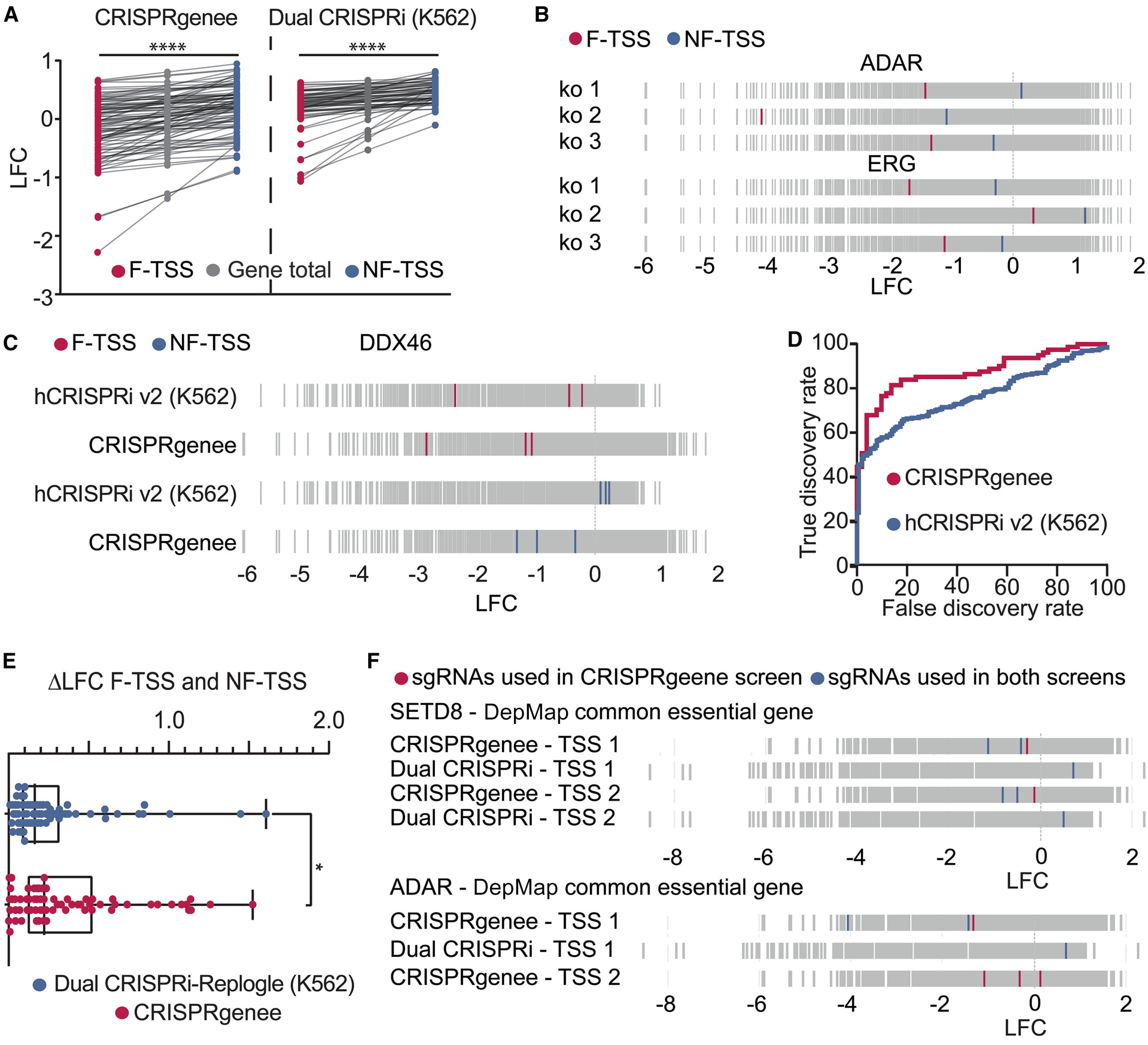

Figure 5. Combination of CRISPRi and CRISPRko has a highly additive effect in a multiplexed LOF screening setup

(A) Differential depletion effect when targeting the functional (F) and non-functional (NF) TSS in the CRISPRgenee screening approach to the effect observed in a published screening dataset using the same sgRNAs.[27]

(B) Performance of individual sgRNA combinations demonstrating that CRISPRgenee improves the KO effect observed when targeting the NF-TSS compared to when targeting the F-TSS of essential genes.

(C) CRISPRgenee rescues nonfunctioning CRISPRi indicated by the depletion observed when the NF-TSS is targeted compared to a published CRISPRi screen using the same sgRNAs.[13]

(D) ROC sensitivity curve of the CRISPRgenee screen compared to a published CRISPRi screen that utilized the same CRISPRi sgRNAs[13] based on sgRNAs targeting essential and non-essential genes.

(E) Individual sgRNA performance comparison of CRISPRgenee with a published screening approach employing a dual-CRISPRi sgRNA strategy targeting the same TSS.[27] The CRISPRi sgRNAs identical in both screens are highlighted (blue) as well as the CRISPRi sgRNA solely used in the CRISPRgenee screen (red).

(F) Boxplot depicting the ΔLFC difference when targeting the F and NF-TSS comparing CRISPRgenee with a published dual-CRISPRi screening dataset[27] (*$p \leq 0.05$; non-parametric Wilcoxon test).

See also Figure S12.

CRISPRgenee constructs were adapted from the CRISPRi-v2 library,[13] we were able to compare the effects of targeting individual TSSs of the essential gene *DDX46*[60] in both CRISPRi-v2 as well as CRISPRgenee screens. The effect observed with sgRNAs for the F-TSS resulted in a strong negative LFC in both screens; however, only the CRISPRgenee approach was able to produce a negative LFC when targeting the NF-TSS (Figure 5C). This improved performance was also visible when

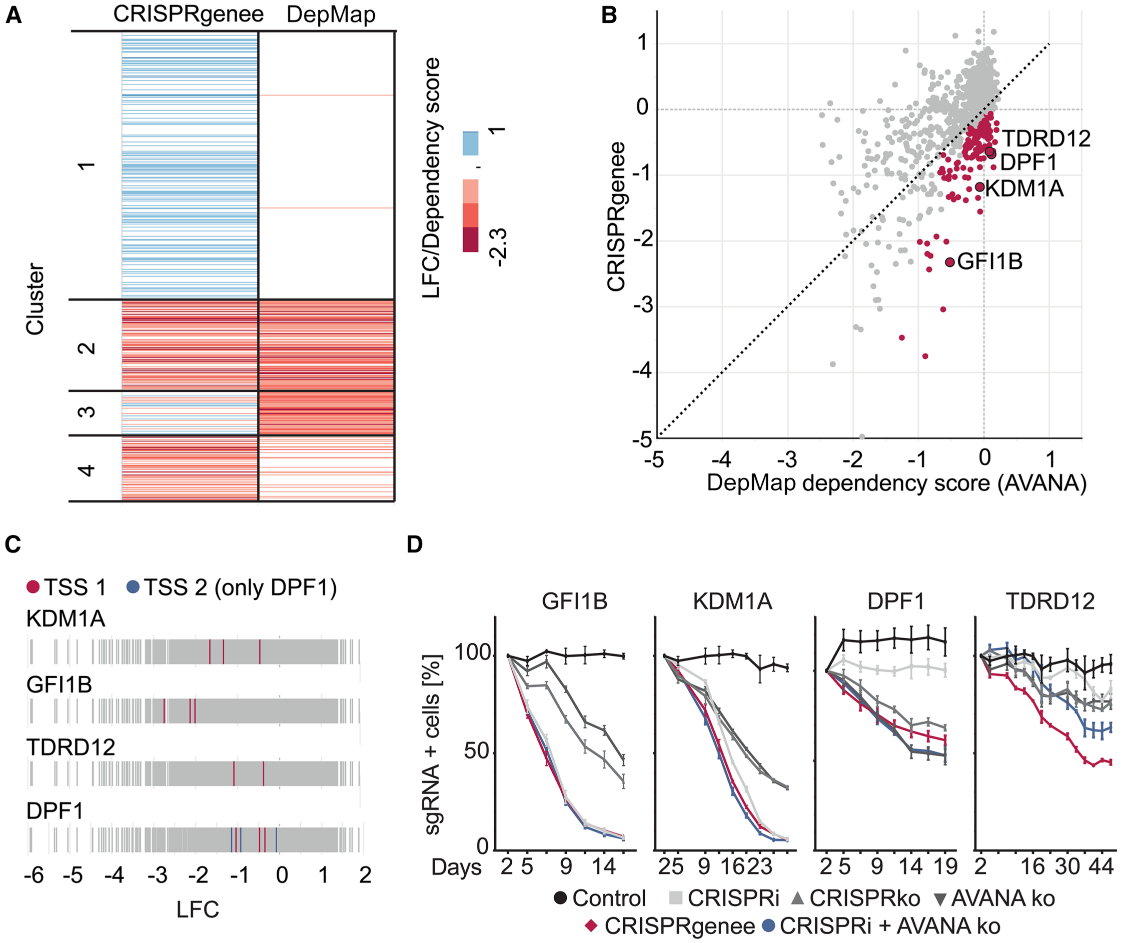

Figure 6. CRISPRgenee can identify novel dependencies in multiplexed LOF screening approaches

(A) Heatmap showing the LFC of genes targeted in the CRISPRgenee screen compared to the dependency score of TF-1 cells from DepMap and clustered into four distinct groups using k-means clustering.

(B) Scatterplot showing the CRISPRgenee gene-level depletion compared to the dependency score from the DepMap portal (https://depmap.org/portal/) for TF-1 cells. Cluster 4 is depicted in red and the differential genes selected for validation of the improved CRISPRgenee effect are highlighted with a black circle.

(C) Individual sgRNA performance of sgRNAs targeting the indicated differential genes.

(D) Competitive proliferation assays in TF-1 cells expressing ZIM3-Cas9 and the indicated sgRNAs. For the validation, the highest-performing CRISPRgenee combination and the resulting single sgRNA controls as well as the best *in silico* predicted CRISPRko sgRNA from the Avana library[16] used to contribute to the DepMap dependency score and a CRISPRgenee combination using this KO sgRNA was used (n = 3, mean ± SEM).

See also Figure S12.

comparing false-positive rates determined by non-essential genes and plotted against the true-positive rate for both screens (Figure 5D) suggesting that combining CRISPRi with CRISPRko leads to a significantly improved gene-dropout effect in multiplexed screening approaches (Figure S12E).

To verify the combinatorial phenotypic effect of our CRISPR genee library, we conducted the same analysis for a dual-CRISPRi screen utilizing the same sgRNAs[13,27] and observed a significantly higher ΔLFC between the sgRNAs targeting individual TSSs of the same gene in the CRISPRgenee approach compared to the dual-CRISPRi screen (Figure 5E). Again, we observed the same improvement when combining a CRISPRi with a CRISPRko in the dropout effect (Figure 5F) as illustrated for *SETD8* and *ADAR* (DepMap common essential genes) (Figure 5F). These data suggest that the additional CRISPRko effect has a greater influence on the LFC than targeting the same TSS with two CRISPRi sgRNAs, as seen when comparing sgRNA performance on essential genes in CRISPRgenee TF-1 screens versus CRISPRi-v2 and dual-CRISPRi LOF screens (Figure S12E).

Hit validation illustrates specific CRISPRgenee benefits

Finally, we benchmarked the CRISPRgenee screen against DepMap dependency scores (https://depmap.org/portal/) for the same cell line, first comparing the essential genes targeted in the CRISPRgenee library and observing similar effects between the CRISPRgenee screen and DepMap annotation (Figure S12F).[61,62] After k-means clustering, we observed four distinct groups (Figure 6A). Group 3 had strong negative DepMap scores but positive/slightly negative CRISPRgenee

effects, whereas group 4 showed strong negative CRISPRgenee LFCs but positive/slightly negative DepMap scores (Figure 6A). From the 147 genes in group 4, we focused on *TDRD12*, *KDM1A*, *GFI1B*, and *DPF1*, which showed minor DepMap depletion but strong CRISPRgenee phenotypic effects (Figure 6B). Interestingly, KDM1A (LSD1) was recently shown to retain residual mRNA and protein in CRISPRko approaches due to alternative splice sites rescuing mutation effects,[2–9] potentially explaining the weak phenotypic effects in previous screens,[61,62] although TF-1 cells are sensitive to LSD1 inhibition.[63] In addition, GFI1B was shown to play an important role in LSD1 biology[64] and scored as the top-20 hit in our screen (Figure 6B). We assessed the performance of all sgRNAs targeting the respective genes, confirming that all single CRISPRgenee sgRNAs resulted in a negative LFC (Figure 6C) and validated the CRISPRgenee benefits, comparing its phenotypic effects to individual sgRNAs used to design the CRISPRgenee construct (CRISPRi-v2 and Vienna Bioactivity CRISPR score)[13,31] as well as the Brunello/AVANA sgRNAs[15] used to derive the dependency score from DepMap. In all cases, CRISPRgenee outperformed single sgRNAs (Figure 6D). So far, the central role of the LSD1 and GFI1B interaction in leukemia[63,64] has never been observed in a multiplexed LOF screening approach, although the LSD1-GFI1B regulatory axis is critical for cell proliferation.[63,64] Again, we identified rescue effects, with one sgRNA of the CRISPRgenee construct contributing more to the negative proliferative effect in case the CRISPRi (*DPF1*, *TDRD12)* or CRISPRko (*GFI1B*, *KDM1A*) did not induce significant effects. These data further highlight the beneficial effect achieved by the combination of CRISPRi and CRISPRko, demonstrating the relevance of the CRISPRgenee system for future LOF approaches.

DISCUSSION

Pooled LOF genetic screens are highly effective in investigating specific phenotypes like proliferation or cell survival. Efforts to reduce library size to reduce cost and increase scalability often rely on fewer sgRNAs per gene,[13,15,16,31,65,66] but this can lead to the discovery of false-positive hits (Figure S12E). Meanwhile, standard CRISPRi and CRISPRko screens face limitations in suppression efficiency, particularly with essential or tightly regulated genes.[17,27,29,67,68] This hinders a broader application of pooled CRISPR LOF screening approaches with complex molecular readouts or when cell numbers are limited.

To address these problems, we developed CRISPRgenee, a dual-sgRNA approach combining a 20-nt sgRNA for Cas9-mediated cleavage and a truncated 15-nt sgRNA for epigenetic repression at the TSS (Figure 1). We confirmed that 15-nt sgRNAs do not induce cleavage and provide faster initial silencing likely due to lower ΔG for duplex unwinding and RNA secondary-structure disruption.[69] This combination significantly improved phenotypic effects, even when single guides were ineffective (Figures 1, 3, 5, and 6), and performed reliably in non-tumorigenic cells like MCF10A and iPSCs without affecting proliferation or differentiation (Figure 3). Finally, we confirmed that CRISPRgenee can also be used as an LOF tool for genetic-dropout screens (Figure 4) performing similarly to comparable pooled LOF screens (Figures 5 and 6).

Truncated guides theoretically raise concerns about off-target binding, but our transcriptomic analysis found no significant differences between 15- and 20-nt sgRNAs (Figures 2 and S3). Consistent with literature, off-target effects are largely governed by the seed region, not PAM-distal bases.[37,70,71] A large portion of Cas9 off-target analysis involves DNA-cleavage assays rather than recruitment assays of Cas9 to DNA. More recent studies investigating dCas9 off-target binding also confirm that mismatches within the seed region lead to severely reduced dCas9 association rates,[41,72] demonstrating that the binding of Cas9 mainly depends on five bases within the seed region.[42,43]

By using three CRISPRgenee sgRNA combinations per gene and requiring two to show consistent effects, we maintain statistical robustness even with reduced library size (Figure S12G).[41]

To streamline dual-sgRNA cloning, we used a compact H1/7SK promoter system under 300 bp, enabling accurate synthesis and low chimera rates ($\sim$5%) compared to $\sim$30% in other systems[27] (Figure S7A). While alternative cloning strategies exist, our design minimizes recombination and supports paired-end sequencing for sgRNA pair validation. However, several sophisticated cloning strategies for the generation of dual-sgRNA libraries have already been published, which can be employed.[26,27,73–75]

Overall, our initial findings that the combination of gene and epigenome engineering improves individual CRISPRko and CRISPRi LOF approaches were confirmed in a multiplexed screening approach, ranking as comparable to or higher than other published screening approaches in terms of consistency in performance across sgRNAs targeting the same gene, thereby increasing the significance of hit calling.

Beyond standard dropout screens, CRISPRgenee can be applied to dual-CRISPRi or CRISPRko formats and adapted to orthogonal screening, including *in vivo* mouse or patient-derived xenograft (PDX) models and primary patient contexts. When combined with emerging technologies such as single-cell and time-resolved transcriptomics,[76–78] orthogonal screening approaches,[79] or organoid model screening,[80] CRISPRgenee offers a powerful tool to dissect genetic dependencies at scale and resolution previously unattainable.

Limitations of the study

As a hybrid of CRISPRi and CRISPRko, CRISPRgenee cannot fully compensate when both sgRNAs are ineffective. This limitation can be mitigated by using $\geq$3 sgRNAs per gene and advanced design algorithms. We also observed high chimera rates during library amplification, likely due to template switching and recombination, which can be reduced through optimized dual-sgRNA cloning strategies.

Although we did not observe adverse effects in our models, Cas9-induced DSBs could potentially trigger DNA damage responses or transient cell-cycle arrest, possibly leading to false positives in essential gene screens.[81] However, prior work indicates single cuts may not activate γH2AX signaling.[82]

The use of truncated sgRNAs for dCas9-ZIM3 targeting increases the theoretical risk of perfect off-target binding. While our transcriptomic analyses did not reveal significant effects,

the full extent of off-targets remains to be determined. Until more precise models emerge, off-target effects should be controlled using conservative thresholds (e.g., requiring consistent LFC reductions from multiple sgRNAs, as applied in our screen).

Looking forward, CRISPRgenee shows strong potential as a go-to approach for LOF studies and high-throughput screening. While its advantages were evident in TF-1 cells, broader applications across diverse cell types, *in vivo* models, and disease contexts are needed to fully assess its generalizability. Continued functional validation will help establish CRISPRgenee's robustness, precision, and therapeutic potential. Addressing current limitations will further refine the system and unlock its full utility in genetic and biomedical research.

RESOURCE AVAILABILITY

Lead contact

Further information and requests for resources and reagents should be directed to and will be fulfilled by the lead contact, Philipp Rathert (Philipp.rathert@ibtb.uni-stuttgart.de).

Materials availability

Plasmids generated in this study are available from Addgene (# 239603, 239604, 239605, 239608, 239609, and 239610).

Data and code availability

- The raw CRISPRgenee screening files, the off-target RNA-seq, as well as amplicon sequencing of the truncated and non-truncated sgRNAs are available at GEO (GEO: GSE238225). All other data are available from the corresponding author upon request.
- This paper does not report original code.
- Any additional information required to reanalyze the data reported in this paper is available from the lead contact upon request.

ACKNOWLEDGMENTS

We thank all laboratory members for constructive discussions and Regina Philipp and Ama Amoateng for technical support. We are grateful to A. Jeltsch for advice throughout this study. We thank the Twist Bioscience team, in particular Tavneet Gill, Xianan Liu, Caitlin Hoeber, and Carlo Antonio Bilbao, for cloning the CRISPRgenee library. pCAG-Eco was a gift from Arthur Nienhuis and Patrick Salmon. pCMVR8.74 was a gift from Didier Trono.

We thank Johannes Zuber (The Research Institute of Molecular Pathology [IMP], Vienna, Austria) for sharing reagents and all members of the Rathert and Jeltsch labs for reagents, protocols, and discussions.

The work described here was supported by the Wilhelm Sander Foundation (2016.082.1 and 2020.055.1) and the German Cancer Aid (70113426). The work of S.A.J. and Z.N. was funded by the Robert Bosch Stiftung. The work of C.B. and F.R.T. was funded by CRC1309 (grant no. 325871075, project C08).

AUTHOR CONTRIBUTIONS

Conceptualization, J.J. and P.R.; data curation, J.S.; formal analysis, J.S. and P.R.; funding acquisition, P.R., S.A.J., S.L., and F.R.T.; investigation, J.S., H.M., J.W., C.B., L.H., and Z.N.; project administration, P.R.; resources, C.B., L.H., M.K., S.L., S.A.J., and F.R.T; supervision, S.L., F.R.T., and P.R.; visualization, J.S. and P.R.; writing – original draft preparation, J.S. and P.R.; writing – review & editing, J.S., J.J., and P.R.

All authors have read and agreed to the published version of the manuscript.

DECLARATION OF INTERESTS

J.J. is an employee of Twist Bioscience.

STAR★METHODS

Detailed methods are provided in the online version of this paper and include the following:

- KEY RESOURCES TABLE
- EXPERIMENTAL MODEL AND STUDY PARTICIPANT DETAILS
  - Cell culture
- METHOD DETAILS
  - Plasmids
  - sgRNA and library cloning
  - Cell culture, lentiviral transduction, generation of Tet-on competent cells and single-cell clones
  - Immunodetection of CD13 and CD33 depletion
  - RNA-seq for off-target detection
  - Competitive proliferation assay
  - Cell cycle analysis
  - Gene expression analysis
  - Amplicon sequencing and T7 endonuclease assay
  - TGFβ induced EMT
  - Neuronal stem cell differentiation and analysis of Tra-1/60
  - Multiplexed CRISPRgenee LOF screen
- QUANTIFICATION AND STATISTICAL ANALYSIS

SUPPLEMENTAL INFORMATION

Supplemental information can be found online at https://doi.org/10.1016/j.crmeth.2025.101078.

Received: May 14, 2024
Revised: August 30, 2024
Accepted: May 19, 2025
Published: June 10, 2025

REFERENCES

1. Bock, C., Datlinger, P., Chardon, F., Coelho, M.A., Dong, M.B., Lawson, K. A., Lu, T., Maroc, L., Norman, T.M., Song, B., et al. (2022). High-content CRISPR screening. Nat. Rev. Methods Primers *2*, 9. https://doi.org/10.1038/s43586-022-00098-7.
2. Smits, A.H., Ziebell, F., Joberty, G., Zinn, N., Mueller, W.F., Clauder-Münster, S., Eberhard, D., Fälth Savitski, M., Grandi, P., Jakob, P., et al. (2019). Biological plasticity rescues target activity in CRISPR knock outs. Nat. Methods *16*, 1087–1093. https://doi.org/10.1038/s41592-019-0614-5.
3. Mou, H., Smith, J.L., Peng, L., Yin, H., Moore, J., Zhang, X.O., Song, C.Q., Sheel, A., Wu, Q., Ozata, D.M., et al. (2017). CRISPR/Cas9-mediated genome editing induces exon skipping by alternative splicing or exon deletion. Genome Biol. *18*, 108. https://doi.org/10.1186/s13059-017-1237-8.
4. van Overbeek, M., Capurso, D., Carter, M.M., Thompson, M.S., Frias, E., Russ, C., Reece-Hoyes, J.S., Nye, C., Gradia, S., Vidal, B., et al. (2016). DNA Repair Profiling Reveals Nonrandom Outcomes at Cas9-Mediated Breaks. Mol. Cell *63*, 633–646. https://doi.org/10.1016/j.molcel.2016.06.037.
5. Allen, F., Crepaldi, L., Alsinet, C., Strong, A.J., Kleshchevnikov, V., De Angeli, P., Páleníková, P., Khodak, A., Kiselev, V., Kosicki, M., et al. (2018). Predicting the mutations generated by repair of Cas9-induced double-strand breaks. Nat. Biotechnol. *37*, 64–72. https://doi.org/10.1038/nbt.4317.
6. Shen, M.W., Arbab, M., Hsu, J.Y., Worstell, D., Culbertson, S.J., Krabbe, O., Cassa, C.A., Liu, D.R., Gifford, D.K., and Sherwood, R.I. (2018). Predictable and precise template-free CRISPR editing of pathogenic variants. Nature *563*, 646–651. https://doi.org/10.1038/s41586-018-0686-x.
7. Chakrabarti, A.M., Henser-Brownhill, T., Monserrat, J., Poetsch, A.R., Luscombe, N.M., and Scaffidi, P. (2019). Target-Specific Precision of

CRISPR-Mediated Genome Editing. Mol. Cell *73*, 699–713.e6. https://doi.org/10.1016/j.molcel.2018.11.031.

8. Anderson, J.L., Mulligan, T.S., Shen, M.C., Wang, H., Scahill, C.M., Tan, F. J., Du, S.J., Busch-Nentwich, E.M., and Farber, S.A. (2017). mRNA processing in mutant zebrafish lines generated by chemical and CRISPR-mediated mutagenesis produces unexpected transcripts that escape nonsense-mediated decay. PLoS Genet. *13*, e1007105. https://doi.org/10.1371/journal.pgen.1007105.
9. Tuladhar, R., Yeu, Y., Tyler Piazza, J., Tan, Z., Rene Clemenceau, J., Wu, X., Barrett, Q., Herbert, J., Mathews, D.H., Kim, J., et al. (2019). CRISPR-Cas9-based mutagenesis frequently provokes on-target mRNA misregulation. Nat. Commun. *10*, 4056. https://doi.org/10.1038/s41467-019-12028-5.
10. Gonatopoulos-Pournatzis, T., Aregger, M., Brown, K.R., Farhangmehr, S., Braunschweig, U., Ward, H.N., Ha, K.C.H., Weiss, A., Billmann, M., Durbic, T., et al. (2020). Genetic interaction mapping and exon-resolution functional genomics with a hybrid Cas9-Cas12a platform. Nat. Biotechnol. *38*, 638–648. https://doi.org/10.1038/s41587-020-0437-z.
11. Larson, M.H., Gilbert, L.A., Wang, X., Lim, W.A., Weissman, J.S., and Qi, L. S. (2013). CRISPR interference (CRISPRi) for sequence-specific control of gene expression. Nat. Protoc. *8*, 2180–2196. https://doi.org/10.1038/nprot.2013.132.
12. Rots, M.G., and Jeltsch, A. (2018). Editing the Epigenome: Overview, Open Questions, and Directions of Future Development. Methods Mol. Biol. *1767*, 3–18. https://doi.org/10.1007/978-1-4939-7774-1_1.
13. Horlbeck, M.A., Gilbert, L.A., Villalta, J.E., Adamson, B., Pak, R.A., Chen, Y., Fields, A.P., Park, C.Y., Corn, J.E., Kampmann, M., and Weissman, J. S. (2016). Compact and highly active next-generation libraries for CRISPR-mediated gene repression and activation. eLife *5*, e19760. https://doi.org/10.7554/eLife.19760.
14. de Groote, M.L., Verschure, P.J., and Rots, M.G. (2012). Epigenetic Editing: targeted rewriting of epigenetic marks to modulate expression of selected target genes. Nucleic Acids Res. *40*, 10596–10613. https://doi.org/10.1093/nar/gks863.
15. Sanson, K.R., Hanna, R.E., Hegde, M., Donovan, K.F., Strand, C., Sullender, M.E., Vaimberg, E.W., Goodale, A., Root, D.E., Piccioni, F., and Doench, J.G. (2018). Optimized libraries for CRISPR-Cas9 genetic screens with multiple modalities. Nat. Commun. *9*, 5416. https://doi.org/10.1038/s41467-018-07901-8.
16. Doench, J.G., Fusi, N., Sullender, M., Hegde, M., Vaimberg, E.W., Donovan, K.F., Smith, I., Tothova, Z., Wilen, C., Orchard, R., et al. (2016). Optimized sgRNA design to maximize activity and minimize off-target effects of CRISPR-Cas9. Nat. Biotechnol. *34*, 184–191. https://doi.org/10.1038/nbt.3437.
17. Gilbert, L.A., Horlbeck, M.A., Adamson, B., Villalta, J.E., Chen, Y., Whitehead, E.H., Guimaraes, C., Panning, B., Ploegh, H.L., Bassik, M.C., et al. (2014). Genome-Scale CRISPR-Mediated Control of Gene Repression and Activation. Cell *159*, 647–661. https://doi.org/10.1016/j.cell.2014.09.029.
18. Haswell, J.R., Mattioli, K., Gerhardinger, C., Maass, P.G., Foster, D.J., Peinado, P., Wang, X., Medina, P.P., Rinn, J.L., and Slack, F.J. (2021). Genome-wide CRISPR interference screen identifies long non-coding RNA loci required for differentiation and pluripotency. PLoS One *16*, e0252848. https://doi.org/10.1371/journal.pone.0252848.
19. Liu, S.J., Horlbeck, M.A., Cho, S.W., Birk, H.S., Malatesta, M., He, D., Attenello, F.J., Villalta, J.E., Cho, M.Y., Chen, Y., et al. (2017). CRISPRi-based genome-scale identification of functional long noncoding RNA loci in human cells. Science *355*, aah7111. https://doi.org/10.1126/science.aah7111.
20. Raffeiner, P., Hart, J.R., García-Caballero, D., Bar-Peled, L., Weinberg, M. S., and Vogt, P.K. (2020). An MXD1-derived repressor peptide identifies noncoding mediators of MYC-driven cell proliferation. Proc. Natl. Acad. Sci. USA *117*, 6571–6579. https://doi.org/10.1073/pnas.1921786117.
21. Tzelepis, K., Koike-Yusa, H., De Braekeleer, E., Li, Y., Metzakopian, E., Dovey, O.M., Mupo, A., Grinkevich, V., Li, M., Mazan, M., et al. (2016). A CRISPR Dropout Screen Identifies Genetic Vulnerabilities and Therapeutic Targets in Acute Myeloid Leukemia. Cell Rep. *17*, 1193–1205. https://doi.org/10.1016/j.celrep.2016.09.079.
22. de Almeida, M., Hinterndorfer, M., Brunner, H., Grishkovskaya, I., Singh, K., Schleiffer, A., Jude, J., Deswal, S., Kalis, R., Vunjak, M., et al. (2021). AKIRIN2 controls the nuclear import of proteasomes in vertebrates. Nature *599*, 491–496. https://doi.org/10.1038/s41586-021-04035-8.
23. Coukos, R., Yao, D., Sanchez, M.I., Strand, E.T., Olive, M.E., Udeshi, N.D., Weissman, J.S., Carr, S.A., Bassik, M.C., and Ting, A.Y. (2021). An engineered transcriptional reporter of protein localization identifies regulators of mitochondrial and ER membrane protein trafficking in high-throughput CRISPRi screens. eLife *10*, e69142. https://doi.org/10.7554/eLife.69142.
24. Liang, J., Zhao, H., Diplas, B.H., Liu, S., Liu, J., Wang, D., Lu, Y., Zhu, Q., Wu, J., Wang, W., et al. (2020). Genome-Wide CRISPR-Cas9 Screen Reveals Selective Vulnerability of ATRX-Mutant Cancers to WEE1 Inhibition. Cancer Res. *80*, 510–523. https://doi.org/10.1158/0008-5472.Can-18-3374.
25. Joberty, G., Fälth-Savitski, M., Paulmann, M., Bösche, M., Doce, C., Cheng, A.T., Drewes, G., and Grandi, P. (2020). A Tandem Guide RNA-Based Strategy for Efficient CRISPR Gene Editing of Cell Populations with Low Heterogeneity of Edited Alleles. CRISPR J. *3*, 123–134. https://doi.org/10.1089/crispr.2019.0064.
26. Diehl, V., Wegner, M., Grumati, P., Husnjak, K., Schaubeck, S., Gubas, A., Shah, V.J., Polat, I.H., Langschied, F., Prieto-Garcia, C., et al. (2021). Minimized combinatorial CRISPR screens identify genetic interactions in autophagy. Nucleic Acids Res. *49*, 5684–5704. https://doi.org/10.1093/nar/gkab309.
27. Replogle, J.M., Bonnar, J.L., Pogson, A.N., Liem, C.R., Maier, N.K., Ding, Y., Russell, B.J., Wang, X., Leng, K., Guna, A., et al. (2022). Maximizing CRISPRi efficacy and accessibility with dual-sgRNA libraries and optimal effectors. eLife *11*, e81856. https://doi.org/10.7554/eLife.81856.
28. Pflueger, C., Tan, D., Swain, T., Nguyen, T., Pflueger, J., Nefzger, C., Polo, J.M., Ford, E., and Lister, R. (2018). A modular dCas9-SunTag DNMT3A epigenome editing system overcomes pervasive off-target activity of direct fusion dCas9-DNMT3A constructs. Genome Res. *28*, 1193–1206. https://doi.org/10.1101/gr.233049.117.
29. Alerasool, N., Segal, D., Lee, H., and Taipale, M. (2020). An efficient KRAB domain for CRISPRi applications in human cells. Nat. Methods *17*, 1093–1096. https://doi.org/10.1038/s41592-020-0966-x.
30. Nuñez, J.K., Chen, J., Pommier, G.C., Cogan, J.Z., Replogle, J.M., Adriaens, C., Ramadoss, G.N., Shi, Q., Hung, K.L., Samelson, A.J., et al. (2021). Genome-wide programmable transcriptional memory by CRISPR-based epigenome editing. Cell *184*, 2503–2519.e2517. https://doi.org/10.1016/j.cell.2021.03.025.
31. Michlits, G., Jude, J., Hinterndorfer, M., de Almeida, M., Vainorius, G., Hubmann, M., Neumann, T., Schleiffer, A., Burkard, T.R., Fellner, M., et al. (2020). Multilayered VBC score predicts sgRNAs that efficiently generate loss-of-function alleles. Nat. Methods *17*, 708–716. https://doi.org/10.1038/s41592-020-0850-8.
32. DeWeirdt, P.C., Sangree, A.K., Hanna, R.E., Sanson, K.R., Hegde, M., Strand, C., Persky, N.S., and Doench, J.G. (2020). Genetic screens in isogenic mammalian cell lines without single cell cloning. Nat. Commun. *11*, 752. https://doi.org/10.1038/s41467-020-14620-6.
33. DeWeirdt, P.C., Sanson, K.R., Sangree, A.K., Hegde, M., Hanna, R.E., Feeley, M.N., Griffith, A.L., Teng, T., Borys, S.M., Strand, C., et al. (2021). Optimization of AsCas12a for combinatorial genetic screens in human cells. Nat. Biotechnol. *39*, 94–104. https://doi.org/10.1038/s41587-020-0600-6.
34. Zhu, S., Cao, Z., Liu, Z., He, Y., Wang, Y., Yuan, P., Li, W., Tian, F., Bao, Y., and Wei, W. (2019). Guide RNAs with embedded barcodes boost CRISPR-pooled screens. Genome Biol. *20*, 20. https://doi.org/10.1186/s13059-019-1628-0.

35. Gonçalves, E., Thomas, M., Behan, F.M., Picco, G., Pacini, C., Allen, F., Vinceti, A., Sharma, M., Jackson, D.A., Price, S., et al. (2021). Minimal genome-wide human CRISPR-Cas9 library. Genome Biol. *22*, 40. https://doi.org/10.1186/s13059-021-02268-4.

36. Pinter, S., Knodel, F., Choudalakis, M., Schnee, P., Kroll, C., Fuchs, M., Broehm, A., Weirich, S., Roth, M., Eisler, S.A., et al. (2021). A functional LSD1 coregulator screen reveals a novel transcriptional regulatory cascade connecting R-loop homeostasis with epigenetic regulation. Nucleic Acids Res. *49*, 4350–4370. https://doi.org/10.1093/nar/gkab180.

37. Fu, Y., Sander, J.D., Reyon, D., Cascio, V.M., and Joung, J.K. (2014). Improving CRISPR-Cas nuclease specificity using truncated guide RNAs. Nat. Biotechnol. *32*, 279–284. https://doi.org/10.1038/nbt.2808.

38. Sternberg, S.H., LaFrance, B., Kaplan, M., and Doudna, J.A. (2015). Conformational control of DNA target cleavage by CRISPR-Cas9. Nature *527*, 110–113. https://doi.org/10.1038/nature15544.

39. Dahlman, J.E., Abudayyeh, O.O., Joung, J., Gootenberg, J.S., Zhang, F., and Konermann, S. (2015). Orthogonal gene knockout and activation with a catalytically active Cas9 nuclease. Nat. Biotechnol. *33*, 1159–1161. https://doi.org/10.1038/nbt.3390.

40. Labun, K., Montague, T.G., Krause, M., Torres Cleuren, Y.N., Tjeldnes, H., and Valen, E. (2019). CHOPCHOP v3: expanding the CRISPR web toolbox beyond genome editing. Nucleic Acids Res. *47*, W171–W174. https://doi.org/10.1093/nar/gkz365.

41. Rostain, W., Grebert, T., Vyhovskyi, D., Pizarro, P.T., Tshinsele-Van Bellingen, G., Cui, L., and Bikard, D. (2023). Cas9 off-target binding to the promoter of bacterial genes leads to silencing and toxicity. Nucleic Acids Res. *51*, 3485–3496. https://doi.org/10.1093/nar/gkad170.

42. Kuscu, C., Arslan, S., Singh, R., Thorpe, J., and Adli, M. (2014). Genome-wide analysis reveals characteristics of off-target sites bound by the Cas9 endonuclease. Nat. Biotechnol. *32*, 677–683. https://doi.org/10.1038/nbt.2916.

43. Wu, X., Scott, D.A., Kriz, A.J., Chiu, A.C., Hsu, P.D., Dadon, D.B., Cheng, A.W., Trevino, A.E., Konermann, S., Chen, S., et al. (2014). Genome-wide binding of the CRISPR endonuclease Cas9 in mammalian cells. Nat. Biotechnol. *32*, 670–676. https://doi.org/10.1038/nbt.2889.

44. Zhang, Y., Song, C., Wang, L., Jiang, H., Zhai, Y., Wang, Y., Fang, J., and Zhang, G. (2022). Zombies Never Die: The Double Life Bub1 Lives in Mitosis. Front. Cell Dev. Biol. *10*, 870745. https://doi.org/10.3389/fcell.2022.870745.

45. Perera, D., Tilston, V., Hopwood, J.A., Barchi, M., Boot-Handford, R.P., and Taylor, S.S. (2007). Bub1 maintains centromeric cohesion by activation of the spindle checkpoint. Dev. Cell *13*, 566–579. https://doi.org/10.1016/j.devcel.2007.08.008.

46. Klebig, C., Korinth, D., and Meraldi, P. (2009). Bub1 regulates chromosome segregation in a kinetochore-independent manner. J. Cell Biol. *185*, 841–858. https://doi.org/10.1083/jcb.200902128.

47. Raaijmakers, J.A., van Heesbeen, R.G.H.P., Blomen, V.A., Janssen, L.M.E., van Diemen, F., Brummelkamp, T.R., and Medema, R.H. (2018). BUB1 Is Essential for the Viability of Human Cells in which the Spindle Assembly Checkpoint Is Compromised. Cell Rep. *22*, 1424–1438. https://doi.org/10.1016/j.celrep.2018.01.034.

48. Currie, C.E., Mora-Santos, M., Smith, C.A., McAinsh, A.D., and Millar, J.B.A. (2018). Bub1 is not essential for the checkpoint response to unattached kinetochores in diploid human cells. Curr. Biol. *28*, R929–R930. https://doi.org/10.1016/j.cub.2018.07.040.

49. Rodriguez-Rodriguez, J.A., Lewis, C., McKinley, K.L., Sikirzhytski, V., Corona, J., Maciejowski, J., Khodjakov, A., Cheeseman, I.M., and Jallepalli, P.V. (2018). Distinct Roles of RZZ and Bub1-KNL1 in Mitotic Checkpoint Signaling and Kinetochore Expansion. Curr. Biol. *28*, 3422–3429.e5. https://doi.org/10.1016/j.cub.2018.10.006.

50. Zhang, G., Kruse, T., Guasch Boldú, C., Garvanska, D.H., Coscia, F., Mann, M., Barisic, M., and Nilsson, J. (2019). Efficient mitotic checkpoint signaling depends on integrated activities of Bub1 and the RZZ complex. EMBO J. *38*, e100977. https://doi.org/10.15252/embj.2018100977.

51. Raaijmakers, J.A., and Medema, R.H. (2019). Killing a zombie: a full deletion of the BUB1 gene in HAP1 cells. Embo j *38*, e102423. https://doi.org/10.15252/embj.2019102423.

52. Meraldi, P. (2019). Bub1-the zombie protein that CRISPR cannot kill. EMBO J. *38*, e101912. https://doi.org/10.15252/embj.2019101912.

53. Galonska, C., Charlton, J., Mattei, A.L., Donaghey, J., Clement, K., Gu, H., Mohammad, A.W., Stamenova, E.K., Cacchiarelli, D., Klages, S., et al. (2018). Genome-wide tracking of dCas9-methyltransferase footprints. Nat. Commun. *9*, 597. https://doi.org/10.1038/s41467-017-02708-5.

54. Broche, J., Kungulovski, G., Bashtrykov, P., Rathert, P., and Jeltsch, A. (2021). Genome-wide investigation of the dynamic changes of epigenome modifications after global DNA methylation editing. Nucleic Acids Res. *49*, 158–176. https://doi.org/10.1093/nar/gkaa1169.

55. Busskamp, V., Lewis, N.E., Guye, P., Ng, A.H.M., Shipman, S.L., Byrne, S.M., Sanjana, N.E., Murn, J., Li, Y., Li, S., et al. (2014). Rapid neurogenesis through transcriptional activation in human stem cells. Mol. Syst. Biol. *10*, 760. https://doi.org/10.15252/msb.20145508.

56. Gao, Z., van der Velden, Y.U., Fan, M., van der Linden, C.A., Vink, M., Herrera-Carrillo, E., and Berkhout, B. (2021). Engineered miniature H1 promoters with dedicated RNA polymerase II or III activity. J. Biol. Chem. *296*, 100026. https://doi.org/10.1074/jbc.RA120.015386.

57. Rathert, P., Roth, M., Neumann, T., Muerdter, F., Roe, J.S., Muhar, M., Deswal, S., Cerny-Reiterer, S., Peter, B., Jude, J., et al. (2015). Transcriptional plasticity promotes primary and acquired resistance to BET inhibition. Nature *525*, 543–547. https://doi.org/10.1038/nature14898.

58. Bauer, T.L., Collmar, K., Kaltofen, T., Loeffler, A.K., Decker, L., Mueller, J., Pinter, S., Eisler, S.A., Mahner, S., Fraungruber, P., et al. (2021). Functional Analysis of Non-Genetic Resistance to Platinum in Epithelial Ovarian Cancer Reveals a Role for the MBD3-NuRD Complex in Resistance Development. Cancers (Basel) *13*, 3801. https://doi.org/10.3390/cancers13153801.

59. Jeong, H.H., Kim, S.Y., Rousseaux, M.W.C., Zoghbi, H.Y., and Liu, Z. (2019). Beta-binomial modeling of CRISPR pooled screen data identifies target genes with greater sensitivity and fewer false negatives. Genome Res. *29*, 999–1008. https://doi.org/10.1101/gr.245571.118.

60. Hart, T., Chandrashekhar, M., Aregger, M., Steinhart, Z., Brown, K.R., MacLeod, G., Mis, M., Zimmermann, M., Fradet-Turcotte, A., Sun, S., et al. (2015). High-Resolution CRISPR Screens Reveal Fitness Genes and Genotype-Specific Cancer Liabilities. Cell *163*, 1515–1526. https://doi.org/10.1016/j.cell.2015.11.015.

61. Tsherniak, A., Vazquez, F., Montgomery, P.G., Weir, B.A., Kryukov, G., Cowley, G.S., Gill, S., Harrington, W.F., Pantel, S., Krill-Burger, J.M., et al. (2017). Defining a Cancer Dependency Map. Cell *170*, 564–576.e16. https://doi.org/10.1016/j.cell.2017.06.010.

62. Meyers, R.M., Bryan, J.G., McFarland, J.M., Weir, B.A., Sizemore, A.E., Xu, H., Dharia, N.V., Montgomery, P.G., Cowley, G.S., Pantel, S., et al. (2017). Computational correction of copy number effect improves specificity of CRISPR-Cas9 essentiality screens in cancer cells. Nat. Genet. *49*, 1779–1784. https://doi.org/10.1038/ng.3984.

63. Ishikawa, Y., Gamo, K., Yabuki, M., Takagi, S., Toyoshima, K., Nakayama, K., Nakayama, A., Morimoto, M., Miyashita, H., Dairiki, R., et al. (2017). A Novel LSD1 Inhibitor T-3775440 Disrupts GFI1B-Containing Complex Leading to Transdifferentiation and Impaired Growth of AML Cells. Mol. Cancer Ther. *16*, 273–284. https://doi.org/10.1158/1535-7163.Mct-16-0471.

64. Maiques-Diaz, A., Spencer, G.J., Lynch, J.T., Ciceri, F., Williams, E.L., Amaral, F.M.R., Wiseman, D.H., Harris, W.J., Li, Y., Sahoo, S., et al. (2018). Enhancer Activation by Pharmacologic Displacement of LSD1 from GFI1 Induces Differentiation in Acute Myeloid Leukemia. Cell Rep. *22*, 3641–3659. https://doi.org/10.1016/j.celrep.2018.03.012.

65. Doench, J.G., Hartenian, E., Graham, D.B., Tothova, Z., Hegde, M., Smith, I., Sullender, M., Ebert, B.L., Xavier, R.J., and Root, D.E. (2014). Rational design of highly active sgRNAs for CRISPR-Cas9-mediated gene inactivation. Nat. Biotechnol. *32*, 1262–1267. https://doi.org/10.1038/nbt.3026.

66. Xu, H., Xiao, T., Chen, C.H., Li, W., Meyer, C.A., Wu, Q., Wu, D., Cong, L., Zhang, F., Liu, J.S., et al. (2015). Sequence determinants of improved CRISPR sgRNA design. Genome Res. *25*, 1147–1157. https://doi.org/10.1101/gr.191452.115.

67. Yeo, N.C., Chavez, A., Lance-Byrne, A., Chan, Y., Menn, D., Milanova, D., Kuo, C.C., Guo, X., Sharma, S., Tung, A., et al. (2018). An enhanced CRISPR repressor for targeted mammalian gene regulation. Nat. Methods *15*, 611–616. https://doi.org/10.1038/s41592-018-0048-5.

68. Carleton, J.B., Berrett, K.C., and Gertz, J. (2017). Multiplex Enhancer Interference Reveals Collaborative Control of Gene Regulation by Estrogen Receptor α-Bound Enhancers. Cell Syst. *5*, 333–344.e5. https://doi.org/10.1016/j.cels.2017.08.011.

69. Alkan, F., Wenzel, A., Anthon, C., Havgaard, J.H., and Gorodkin, J. (2018). CRISPR-Cas9 off-targeting assessment with nucleic acid duplex energy parameters. Genome Biol. *19*, 177. https://doi.org/10.1186/s13059-018-1534-x.

70. Fu, Y., Foden, J.A., Khayter, C., Maeder, M.L., Reyon, D., Joung, J.K., and Sander, J.D. (2013). High-frequency off-target mutagenesis induced by CRISPR-Cas nucleases in human cells. Nat. Biotechnol. *31*, 822–826. https://doi.org/10.1038/nbt.2623.

71. Hsu, P.D., Scott, D.A., Weinstein, J.A., Ran, F.A., Konermann, S., Agarwala, V., Li, Y., Fine, E.J., Wu, X., Shalem, O., et al. (2013). DNA targeting specificity of RNA-guided Cas9 nucleases. Nat. Biotechnol. *31*, 827–832. https://doi.org/10.1038/nbt.2647.

72. Boyle, E.A., Andreasson, J.O.L., Chircus, L.M., Sternberg, S.H., Wu, M.J., Guegler, C.K., Doudna, J.A., and Greenleaf, W.J. (2017). High-throughput biochemical profiling reveals sequence determinants of dCas9 off-target binding and unbinding. Proc. Natl. Acad. Sci. USA *114*, 5461–5466. https://doi.org/10.1073/pnas.1700557114.

73. Wegner, M., Diehl, V., Bittl, V., de Bruyn, R., Wiechmann, S., Matthess, Y., Hebel, M., Hayes, M.G., Schaubeck, S., Benner, C., et al. (2019). Circular synthesized CRISPR/Cas gRNAs for functional interrogations in the coding and noncoding genome. eLife *8*, e42549. https://doi.org/10.7554/eLife.42549.

74. Li, R., Klingbeil, O., Monducci, D., Young, M.J., Rodriguez, D.J., Bayyat, Z., Dempster, J.M., Kesar, D., Yang, X., Zamanighomi, M., et al. (2022). Comparative optimization of combinatorial CRISPR screens. Nat. Commun. *13*, 2469. https://doi.org/10.1038/s41467-022-30196-9.

75. Sánchez-Rivera, F.J., Diaz, B.J., Kastenhuber, E.R., Schmidt, H., Katti, A., Kennedy, M., Tem, V., Ho, Y.J., Leibold, J., Paffenholz, S.V., et al. (2022). Base editing sensor libraries for high-throughput engineering and functional analysis of cancer-associated single nucleotide variants. Nat. Biotechnol. *40*, 862–873. https://doi.org/10.1038/s41587-021-01172-3.

76. Dixit, A., Parnas, O., Li, B., Chen, J., Fulco, C.P., Jerby-Arnon, L., Marjanovic, N.D., Dionne, D., Burks, T., Raychowdhury, R., et al. (2016). Perturb-Seq: Dissecting Molecular Circuits with Scalable Single-Cell RNA Profiling of Pooled Genetic Screens. Cell *167*, 1853–1866.e17. https://doi.org/10.1016/j.cell.2016.11.038.

77. Adamson, B., Norman, T.M., Jost, M., Cho, M.Y., Nuñez, J.K., Chen, Y., Villalta, J.E., Gilbert, L.A., Horlbeck, M.A., Hein, M.Y., et al. (2016). A Multiplexed Single-Cell CRISPR Screening Platform Enables Systematic Dissection of the Unfolded Protein Response. Cell *167*, 1867–1882.e21. https://doi.org/10.1016/j.cell.2016.11.048.

78. Datlinger, P., Rendeiro, A.F., Schmidl, C., Krausgruber, T., Traxler, P., Klughammer, J., Schuster, L.C., Kuchler, A., Alpar, D., and Bock, C. (2017). Pooled CRISPR screening with single-cell transcriptome readout. Nat. Methods *14*, 297–301. https://doi.org/10.1038/nmeth.4177.

79. Cetin, R., Wegner, M., Luwisch, L., Saud, S., Achmedov, T., Süsser, S., Vera-Guapi, A., Müller, K., Matthess, Y., Quandt, E., et al. (2023). Optimized metrics for orthogonal combinatorial CRISPR screens. Sci. Rep. *13*, 7405. https://doi.org/10.1038/s41598-023-34597-8.

80. Ringel, T., Frey, N., Ringnalda, F., Janjuha, S., Cherkaoui, S., Butz, S., Srivatsa, S., Pirkl, M., Russo, G., Villiger, L., et al. (2020). Genome-Scale CRISPR Screening in Human Intestinal Organoids Identifies Drivers of TGF-β Resistance. Cell Stem Cell *26*, 431–440.e8. https://doi.org/10.1016/j.stem.2020.02.007.

81. Aguirre, A.J., Meyers, R.M., Weir, B.A., Vazquez, F., Zhang, C.Z., Ben-David, U., Cook, A., Ha, G., Harrington, W.F., Doshi, M.B., et al. (2016). Genomic Copy Number Dictates a Gene-Independent Cell Response to CRISPR/Cas9 Targeting. Cancer Discov. *6*, 914–929. https://doi.org/10.1158/2159-8290.Cd-16-0154.

82. van den Berg, J., G Manjón, A., Kielbassa, K., Feringa, F.M., Freire, R., and Medema, R.H. (2018). A limited number of double-strand DNA breaks is sufficient to delay cell cycle progression. Nucleic Acids Res. *46*, 10132–10144. https://doi.org/10.1093/nar/gky786.

83. Bolger, A.M., Lohse, M., and Usadel, B. (2014). Trimmomatic: a flexible trimmer for Illumina sequence data. Bioinformatics *30*, 2114–2120. https://doi.org/10.1093/bioinformatics/btu170.

84. Kim, D., Langmead, B., and Salzberg, S.L. (2015). HISAT: a fast spliced aligner with low memory requirements. Nat. Methods *12*, 357–360. https://doi.org/10.1038/nmeth.3317.

85. Love, M.I., Huber, W., and Anders, S. (2014). Moderated estimation of fold change and dispersion for RNA-seq data with DESeq2. Genome Biol. *15*, 550. https://doi.org/10.1186/s13059-014-0550-8.

STAR★METHODS

KEY RESOURCES TABLE

REAGENT or RESOURCE	SOURCE	IDENTIFIER
**Antibodies**		
Anti-CD90.1(Thy1.1)-APC	Thermo Fisher	AB_469420
Anti-CD90.1(Thy1.1)-APC	BioLegend	AB_1595470
PE anti-human CD33	BioLegend	AB_314347
PE anti-human CD13	BioLegend	AB_314179
anti-CD324 (E-Cadherin) - clone 4A2 monoclonal mouse	Cell signaling	AB_2728770
anti-Vimentin - clone D21H3 - monoclonal rabbit	Cell signaling	AB_10695149
Alexa Fluor 647 goat anti-mouse IgG (H + L)	Thermo Fisher	AB_2536165
Alexa Fluor 488 goat anti-rabbit IgG (H + L)	Thermo Fisher	AB_143165
TRA-1-60 Antibody, anti-human, REAfinity™	Miltenyi Biotec	AB_2654228
**Bacterial and virus strains**		
E. coli Stbl3™	Invitrogen	C737303
**Chemicals, peptides, and recombinant proteins**		
Recombinant human GM-CSF	Miltenyi Biotec	130-095-372
hHOLO-Transferrin	Merck Millipore	616424
hrInsulin	Sigma-Aldrich	I9278
hFGF-2-IS	Miltenyi Biotec	130-104-921
hrTGF-b1	Miltenyi Biotec	130-095-067
**Deposited data**		
CRISPRgate screen, RNA-seq and amplicon seq	This paper	https://www.ncbi.nlm.nih.gov/geo/query/acc.cgi?acc=GSE238225
**Experimental models: Cell lines**		
NIH3T3	ATCC	CRL-1658
LentiX293T	Takara Bio	632180
HEK293T	DSMZ	ACC 635
TF-1	ATCC	CRL-2003
iNGNs	Collaboration partner	N/A
MCF10A	ATCC	CRL-10317
**Oligonucleotides**		
Primers for cloning, amplification cleavage site, qPCR and illumina sequencing see Table S1	This paper	N/A
sgRNAs used in this study see Table S2	This paper	N/A
CRISPRgate library see Table S6	This paper	N/A
**Recombinant DNA**		
Plasmids generated in this study are deposited at addgene see Table S3	N/A	N/A
**Software and algorithms**		
Graphpad Prism 5	Graphpad Software, Inc	N/A
Illustrator CS6	Adobe	N/A
Excel 2016	Microsoft	N/A
Tableau 2023.2	Tableau	N/A
R	The R Project	N/A
R-studio	Posit	N/A

(*Continued on next page*)

Continued

REAGENT or RESOURCE	SOURCE	IDENTIFIER
Other		
Normalized screen count of all analyzed screens in this publication see Table S7	This paper	N/A
Normalized RNA-seq count and DE-seq analysis see Table S5	This paper	N/A
Amplicon sequencing count see Table S4	This paper	N/A

EXPERIMENTAL MODEL AND STUDY PARTICIPANT DETAILS

Cell culture

All media were supplemented with 10% Fetal Bovine Serum, 4 mM L-Glutamine, 10 mM HEPES, 1 mM Sodium pyruvate solution, 100 U/mL Penicillin and 100 μg/mL Streptomycin. NIH/3T3 (male), Lenti-X293T (female) and HEK293T (female) cells were cultivated in DMEM high glucose media (Sigma-Aldrich) and TF-1 (male) cells were cultivated in RPMI 1640 supplemented with 2 ng/mL of recombinant human GM-CSF (130-095-372 Miltenyi Biotec). MCF10A cells were seeded in in DMEM/F12 medium (Thermo Fisher, #21331020) supplemented with 100 ng/mL cholera toxin (Sigma, #C8052), 20 ng/mL epidermal growth factor (EGF) (Preprotech, #AF-100-15), 10 μg/mL insulin (Sigma, #I9278), 500 ng/mL hydrocortisone (Sigma, #H0888), GlutaMax (Thermo Fisher, #35050038), 5% horse serum (Thermo Fisher, #16050122), 1× penicillin/streptomycin (Thermo Fisher, #15140122). iNGNs[55] were grown on Geltrex (ThermoScientific #A1413202)-coated tissue plates and cultivated at 37°C in water-saturated, CO_2-enriched (5%) atmosphere. Uninduced iNGNs were cultured in hiPSCs-medium (1:1 DMEM:F-12, GlutaMAX-Supplement (Gibco #10565018), supplemented with 0.2 mM L-ascorbic acid 2-phosphate, 77.6 nM sodium selenite, 10.90 mM NaCl, 0.1 mM nicotinamide, 10 μg/mL hHOLO-Transferrin (Merck Millipore #616424), 20 μg/mL hrInsulin (Sigma-Aldrich #I9278), 20 ng/mL hFGF-2-IS (Miltenyi Biotec #130-104-921), 2.0 ng/mL hrTGF-b1 (Miltenyi Biotec #130-095-067).

METHOD DETAILS

Plasmids

The DNA sequence encoding ZIM3-KRAB (ZIM3), without the stop codon, was synthesized and cloned in front of Cas9 in a Dox inducible pRRL-TRE3G-Cas9-P2A-GFP plasmid kindly provided by the Zuber lab[22] using standard cloning methods. ZIM3-Cas9 expression was coupled to GFP via a P2A element. Additionally, antibiotic resistance against Blasticidin driven by a PGK promoter was added to select for positively transduced cells (TRE3G-ZIM3-Cas9-NLS-P2A-GFP-PGK-BlastR). For the constitutive CRISPRgenee vector, the ZIM3 element without the stop codon was cloned in front of Cas9 of an EF1as-Cas9-P2A-GFP plasmid using standard cloning methods. Additionally, a Blasticidin resistance driven by a PGK promoter was added. For dual sgRNA expression, a dual filler plasmid was cloned adding an additional tracr and promoter to a lentiviral single sgRNA expression vector kindly provided by the Zuber lab[22] using an hU6 promoter to express the first sgRNA and either the H1 or minimal H17SK to express the second plasmid. Successful transduction of the dual sgRNA expressing plasmid was monitored via an outer membrane protein (Thy1.1) driven by an Ef1a short promoter and coupled to Neomycin resistance via a P2A element (hU6-filler1-tracr-H1/minH17SK-filler2-tracr-Ef1as-Thy1.1-P2A-Neo).

sgRNA and library cloning

The CRISPRi and CRISPRko sgRNAs were ordered as complementary oligos harboring overhangs fitting to the BsmBI restriction site of the respective fillers. The complementary sgRNA oligos were phosphorylated, annealed and afterward cloned into the dual-sgRNA filler plasmid using golden-gate assembly.

For the pooled sgRNA library we designed 270 bp long oligo fragments consisting of the 15 nt CRISPRi sgRNA, a tracr, the minimal H1/7SK promoter and the 20 nt CRISPRko sgRNA. TWIST Biosciences pool synthesized the oligo fragments and cloned these into the filler of a PRRL lentiviral sgRNA vector (PRRL-PBS-hU6-filler-tracr-Ef1as-Thy1.1-P2A-Neo) harboring a primer binding site for PCR amplification for Illumina sequencing kindly provided by the Zuber lab.[22] TWIST performed library quality control and deep sequencing, identifying an initial chimera rate of 9.78%.

Cell culture, lentiviral transduction, generation of Tet-on competent cells and single-cell clones

Generation of Tet-on competent cells was performed as previously described.[57] For lentiviral packaging of pRRL-vectors, plasmids were mixed with helper plasmids pCMVR8.74 (Addgene plasmid #22036) and pCAG-Eco (Addgene plasmid #35617) and 3× (w/w) excess of polyethyleneimine 25K in DMEM. The mix was added dropwise to LentiX cells at 70–80% confluency. Media was exchanged after 12 and 24 h. The virus particles were harvested 48h after transfection with an optional second and third harvest after 56 to 72 h. To prevent contamination of the target cells with LentiX, the virus was filtered using a 45 μm filter. pRRL

vectors for expression of ZIM3-Cas9 were introduced into the Tet-on competent target cells by transduction at a transduction efficiency <20% to ensure single plasmid integration. For selection, TF-1 cells were treated with 4 μg/mL Blasticidin. The dual sgRNA expression vector was transduced and the cells were selected with 500 μg/mL G418 solution for 7 days. Afterward, expression of ZIM3-Cas9 was induced by the addition of 1 μg/mL Doxycycline (Dox). Successful integration and expression of the desired plasmids was analyzed two days after transduction and monitored throughout selection and flow cytometry using a MACSQuant Vyb flow cytometer. To generate single-cell clones for the CRISPRgenee screen, expression of ZIM3-Cas9 was induced in TF-1 cells and single-cell clones were sorted based on the GFP fluorescence using the Sony SH800S FACS. The single-cell clones were monitored based on the GFP signal, keeping the cells that were able to reversibly induce ZIM3-Cas9 expression by addition and removal of Dox. We then validated the single-cell clones by transducing these with sgRNAs targeting CD13 and CD33, induced the expression of ZIM3-Cas9 using Dox and monitored the depletion of CD13/CD33. We chose three distinct sgRNAs which all showed similar CD13/CD33 depletion efficiencies and were able to specifically respond to Dox induction and removal.

Immunodetection of CD13 and CD33 depletion

sgRNAs targeting CD13 and CD33 (Table S2) were stably integrated using lentiviral transduction. ZIM3-Cas9 expression was induced by the addition of 1 μg/mL Doxycycline (Dox) and the loss of functional CD13 and CD33 was monitored every two days for 14 days through AB staining of CD13 and CD33 and detection by flow cytometry.

RNA-seq for off-target detection

Two sgRNAs targeting *CD33*, were selected based on their *in-silico* predicted off-target activity (minimum of one perfect off-target for the 15mer) and cloned as standard 20 nt sgRNA or the respective 15 nt truncation. The sgRNAs targeting *CD33* as well as a scrambled sgRNA (scr) were transduced into dCas9-tagBFP-ZIM3 expressing cells and selected for one week. After two weeks of sgRNA expression, the cells were sorted based on the dCas9-ZIM3 and sgRNA expression, harvested and RNA was isolated using the RNeasy Plus Mini Kit (QIAGEN, Hilden, Germany). RNA concentration and quality were assessed using the 260/280 and 260/230 ratios obtained at the NanoDrop. We enriched for mRNA using the NEBNext Poly(A) mRNA Magnetic Isolation Module (NEB) and used this as input for library generation using the NEBNext Ultra II RNA Library Prep (NEB). The quality and concentration of the cDNA library was assessed using the BioAnalyzer 2100 high sensitivity DNA kit. In case adapter fragments were detected, size exclusion DNA purification using NEBNext Sample Purification Beads (NEB) was performed according to the manufacturer's instructions. The samples were sent for deep sequencing and the obtained reads were filtered using Trimmomatic[83] removing adapter contamination and filtering reads based on sequencing quality, keeping high-quality reads for further analysis. Next, we aligned both forward and reverse reads to the human genome (hg38) using HISAT2[84] and obtained the gene count files, which were used for DESeq2 analysis[85] comparing both sgRNAs (15 vs. 20) with each other as well as comparing the individual sgRNAs and the scr control (Table S5).

Competitive proliferation assay

TF-1 cells were transduced with the indicated sgRNAs (Table S2) and ZIM3-Cas9 expression was induced using Dox. The amount of Cas9 and sgRNA positive cells were measured after two days and set as reference. The proliferation of ZIM3-Cas9 and sgRNA-positive cells were monitored for the indicated time points. HEK293T cells were transduced with the indicated sgRNAs and selected for one week. Afterward, 80% of sgRNA-positive cells were mixed with 20% WT cells, and expression of ZIM3-Cas9 was induced using Dox. Two days after, the amount of sgRNA and ZIM3-Cas9 positive cells were measured and set as reference, and the proliferation was monitored for the indicated time points.

Cell cycle analysis

200 000 HEK293T cells were harvested after five days of BUB1 depletion and fixed in 70% ice-cold ethanol for 2h. Afterward, ethanol was removed by centrifugation and the pellet was resuspended in 200 μL cell cycle staining solution (100 μg/mL RNAseA, 50 μg/mL PI, 0.1% Triton X- in PBS) and incubated for 30 to 60 min at room temperature in the dark. Directly after incubation, the DNA content was measured by flow cytometry and the cell cycle tool (model Dean-Jett-Fox) of FlowJo was used to automatically detect the cell cycle phase distribution of the different samples.

Gene expression analysis

Cells were harvested after five days of BUB1 depletion, and mRNA was extracted by using the RNeasy Plus Mini Kit (QIAGEN, Hilden, Germany). RNA concentration and quality was assessed using the 260/280 and 260/230 ratios obtained at the NanoDrop. Reverse transcription was performed with 500 ng of purified RNA using oligo(dT)$_{18}$ primers for the Multiscribe Reverse Transcriptase (Invitrogen) according to the manufacturer's instructions. Quantitative PCR reactions were carried out using the CFX Connect Real-Time System from Bio Rad using the ORA SEE qPCR Green ROX H Mix (highQu) and the human *BUB1* and *ACTIN* primer set (Table S1). The following cycling conditions were performed: 95°C for 3 min, 39 cycles of 95°c for 5 s then 60°C for 30s with the cycling conditions for the melt curve performed afterward.

Amplicon sequencing and T7 endonuclease assay

TF-1 cells were transduced with 20 nt or 15 nt long sgRNAs targeting either *CD13* or *CD33* as well as with a scr control sgRNA and selected for seven days. ZIM3-Cas9 expression was turned on using Dox and the cells were harvested after 14 days. Primers were designed to amplify the sgRNA targeting region either for amplicon sequencing or mismatch cleavage assay (Table S1). Genomic DNA of >1 Mio sgRNA positive and WT cells was amplified and extracted from an agarose gel and the DNA concentration was determined. For amplicon sequencing, Illumina adapters were added to the PCR product using a second PCR step, the resulting PCR amplicons were then extracted from an agarose gel. The size, concentration, and purity of the DNA amplicon was assessed using the BioAnalyzer 2100 high sensitivity DNA kit. All amplicons were pooled and sent for Illumina sequencing. For analysis, the reads were split based on the internal barcode, the site of cleavage was identified and the percentage of InDels was calculated for the scr control, 15 nt and 20 nt sgRNAs (Table S4). For the T7 assay, 200 ng of PCR product (WT and sgRNA) was stepwise annealed and T7 endonuclease was added. Cleaved fragments were determined using agarose gel electrophoresis.

TGFβ induced EMT

MCF10A cells stably expressing dCas9-ZIM3 or ZIM3-Cas9 were transduced with sgRNAs targeting *SMAD2*, the cells were stained using PE-labeled antibodies against Thy1.1 (receptor co-expressed with the sgRNA) and sgRNA-positive cells were sorted two days after transduction. After one week of sgRNA expression cells were supplemented with 1:1000 Doxycycline to induce the expression of ZIM3-Cas9. One day after initial seeding, MCF10A cells were treated for 8 days with 100 pM TGFβ1 (Prepotech, #100-21C) with restimulation every 48 h, and harvested by TrypLETM (ThermoFisher, #12604013). For cell fixation and permeabilization, 100.000 cells per condition were treated with 4% PFA in DPBS for 15 min, and 0.1% Triton in FACS media (DPBS +5% FHS) for 15 min at 4°C. Cells were then incubated with anti-CD324 (E-Cadherin) antibody (1:150) and anti-Vimentin antibody (1:150) for 1 h. After blocking in FACS media for 10 min, cells were incubated with secondary antibodies Alexa Fluor 647 goat anti-mouse IgG (H + L), (1:1000) and Alexa Fluor 488 goat anti-rabbit IgG (H + L) (1:250) for 45 min. All antibody incubations were performed in FACS media. Cells were washed between fixation with DPBS and during antibody staining twice with FACS media and afterward analyzed by flow cytometry. For validation of the SMAD2 depletion, a subset of cells was harvested before TGFβ stimulation, and the RNA was isolated and reverse-transcribed into cDNA. Using specific primers for *SMAD2* (Table S1) the relative expression compared to *ACTIN* was measured using qPCR as described in "Gene expression analysis".

Neuronal stem cell differentiation and analysis of Tra-1/60

iNGNs were passaged in the uninduced state when they reached about 70–80% confluency. For passaging, 2 μM (final concentration) of thiazovivin (Merck Millipore 420220) was added to the hiPSCs medium for 24 h, afterward medium was changed to hiPSCs-medium without thiazovivin. iNGNs were stably transduced with EF1a-ZIM3-Cas9-P2A-GFP-PGK-BlastR and selected for one week using 2μg/mL blasticidin. Successful selection and expression of ZIM3-Cas9 was monitored by measuring the expression of GFP at the MacsQuant. Afterward, sgRNAs targeting ART1 or Ngn2 were stably transduced and cells were selected for one week using 100–200 μg/mL G418 solution. For induction of neuronal differentiation, the medium was changed to induction medium (hiPSCs-medium without FGF2-IS and TGF-β1), and Doxycycline (Sigma-Aldrich #D9891) was added to a final concentration of 0.5 μg/mL. Neuronal differentiation was induced for three days before harvesting for qPCR and Tra-1/60 antibody staining.

Multiplexed CRISPRgenee LOF screen

A CRISPRgenee library designed to target 1137 target genes involved in chromatin regulation using a set of 3686 sgRNAs (Table S6) was transduced into three independent TF-1 cell clones with comparable ZIM3-Cas9 expression levels of with an sgRNA representation of 1 000 ×. After 7 days of antibiotic selection, ZIM3-Cas9 expression was induced using Dox and cells were cultivated for 14 days (7 passages, 70 h doubling time). During cultivation, sgRNA representation and ZIM3-Cas9 expression was monitored using flow cytometry. After 14 days (7 passages), genomic DNA for the three single-cell clones was isolated using phenol-extraction using PhaseLock tubes, followed by ethanol precipitation. Multiple parallel 50 μL PCR reactions, each containing 1 μL gDNA template adding up to 40 μg gDNA and 300 ng for the library pool, using the AmpliTaq Gold Polymerase (Life Technologies) were performed to maintain sgRNA representation. In a first round of PCRs, random barcodes and sample barcodes were added to the sgRNA sequences using the following cycling parameters: 95°C for 10 min; 28 cycles of (95°C for 30 s, 54°C for 45 s and 72°C for 60 s); 72°C for 7 min. PCR products for each single cell clone were combined and purified using the NucleoSpin Gel and PCR clean-up kit (Macherey-Nagel). Afterward using a second round of PCR, using similar cycling conditions with the exception of 10 ng input as template and 7 total cycles the standard Illumina P7 and P5 adaptors were added. All primers used for the library preparation are listed in Table S1. The final libraries were cleaned up from a 2% agarose gel, pooled, and analyzed on a P2 flow cell (400 mio reads) using the Illumina NextSeq 2000 with a 35% PhiX spike-in (75 bp paired-end), using standard Illumina primers. Sequence processing was performed using a custom Galaxy workflow (www.usegalaxy.eu). sgRNA count data, as well as data used for comparison from other screens, are provided in Table S7. Forward and reverse reads were combined and non-mapped reads as well as sgRNA chimeras were filtered out. The read count for each sgRNA combination was normalized as count per million using the r-studio tool "CB2".[59] Using the same tool fold depletion of individual sgRNA combinations as well as for the targeted genes was calculated.

QUANTIFICATION AND STATISTICAL ANALYSIS

All details regarding statistical analysis are provided in the respective figure legends and figures, including numbers of replicates for each experiment, statistical tests used and the obtained *p* values. Results are presented as means ± standard error of the mean [S.E.M.]. If not stated otherwise, statistical significance was calculated by two-way ANOVA with a post-hoc test indicated for each experiment with $p \leq 0.05$ considered statistically significant. Statistical significance levels are denoted as follows: ****$p \leq 0.0001$; ***$p \leq 0.001$; **$p \leq 0.01$; *$p \leq 0.05$; n.s. = non-significant.

Cell Reports Methods, Volume *5*

Supplemental information

CRISPR GENome and epigenome engineering improves loss-of-function genetic-screening approaches

Jannis Stadager, Chiara Bernardini, Laura Hartmann, Henrik May, Jessica Wiepcke, Monika Kuban, Zeynab Najafova, Steven A. Johnsen, Stefan Legewie, Franziska R. Traube, Julian Jude, and Philipp Rathert

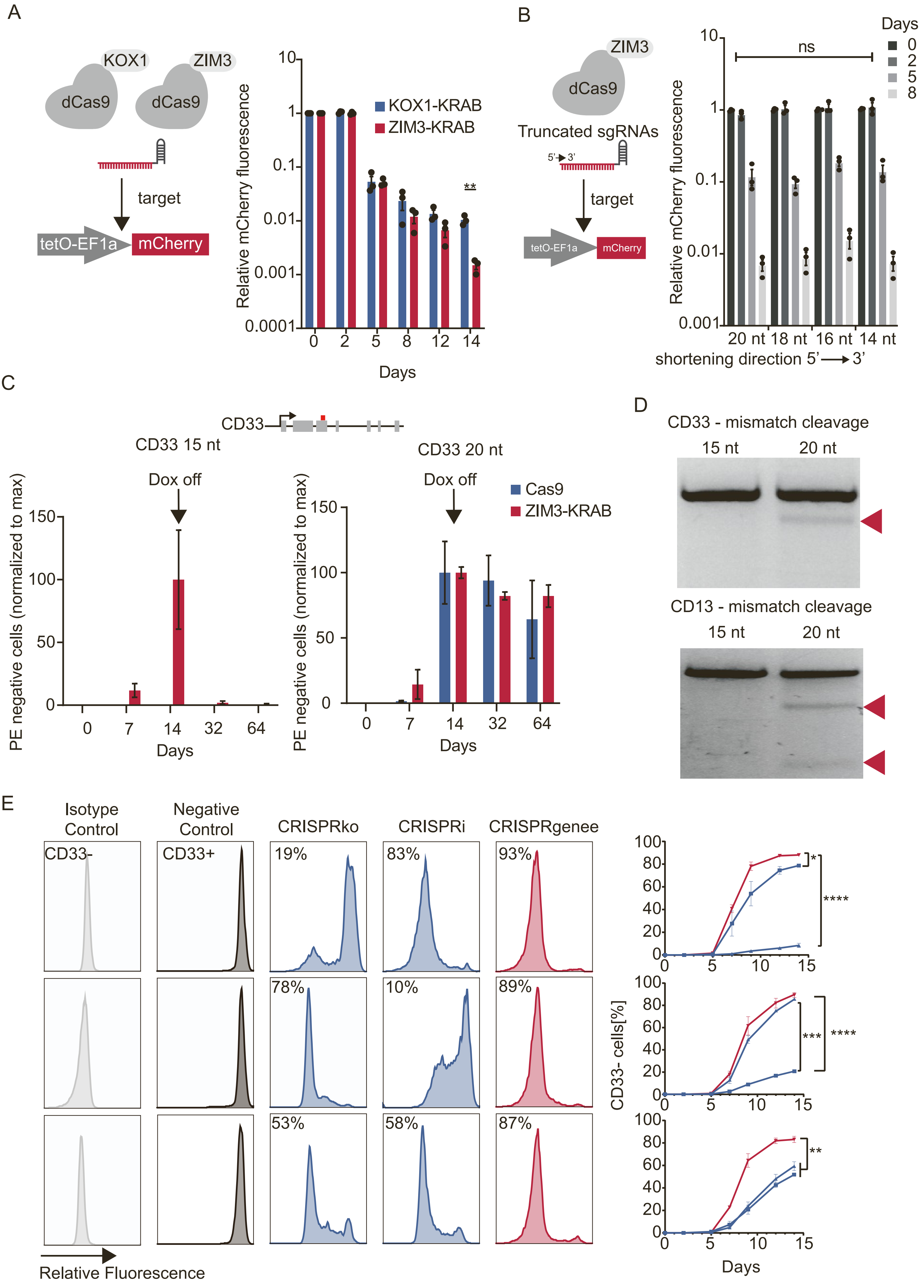
A
KOX1
ZIM3
dCas9
dCas9
target
tetO-EF1a
mCherry
Relative mCherry fluorescence
KOX1-KRAB
ZIM3-KRAB
**
Days
B
ZIM3
dCas9
Truncated sgRNAs
5'→3'
target
tetO-EF1a
mCherry
Relative mCherry fluorescence
ns
Days
0
2
5
8
20 nt 18 nt 16 nt 14 nt
shortening direction 5'→3'
C
CD33
CD33 15 nt
Dox off
PE negative cells (normalized to max)
Days
CD33 20 nt
Dox off
Cas9
ZIM3-KRAB
PE negative cells (normalized to max)
Days
D
CD33 - mismatch cleavage
15 nt
20 nt
CD13 - mismatch cleavage
15 nt
20 nt
E
Isotype Control
Negative Control
CRISPRko
CRISPRi
CRISPRgenee
CD33-
CD33+
19%
83%
93%
78%
10%
89%
53%
58%
87%
Relative Fluorescence
CD33- cells[%]
Days
*
****
***
****
**

Figure S1: Validation of the CRISPRgenee KRAB domain and setup of the proof of concept experiment. Related to Figure 1

(A) Flow-cytometric analysis of the mCherry expression in NIH/3T3 cells co-expressing the mCherry reporter, the dCas9-ZIM3 or dCas9-KOX1 fusion proteins and a sgRNA targeting the promoter region of the reporter. (n = 3, mean ± S.D..; **P ≤ 0.01, n.s. = non-significant; multiple t-tests with a two-stage set-up method of Benjamin to account for FDR).

(B) Flow-cytometric analysis of the mCherry expression in NIH/3T3 cells co-expressing the mCherry reporter, the dCas9-ZIM3 fusion protein, and truncated sgRNAs (5' to 3') with the indicated length targeting the promoter region of the reporter. (n = 3, mean ± S.D..; n.s. = non-significant, ordinary two-way ANOVA with Tukey post-hoc test).

(C) Quantification of CD33 expression by immunostaining of TF-1 erythroleukemia cells expressing Cas9 (blue) or ZIM3-Cas9 (red) and identical sgRNAs either 15 nt or 20 nt long. ZIM3-Cas9/Cas9 expression was induced for 14 days and afterward, on day 14 the percentage of CD33 negative cells was set as a reference, dox was removed and the recovery of the CD33 expression was monitored to determine the consistency of the induced genomic changes. (n = 3, mean ± S.D).

(D) Representative gel image of a DNA mismatch cleavage assay at the end of the experiment determining non-homologous end-joining efficiency for the indicated sgRNAs at the CD13 and CD33 genes. Red arrowheads indicate cleavage products due to mismatches between WT CD13/CD33 DNA and Cas9-targeted CD13/CD33 genes.

(E) Flow cytometry quantification of the isotype and non-targeting control as well as for different *CD33* targeting sgRNAs and the respective CRISPRgenee combination after 14 days as well as the overall time course of CD33 depletion (n = 3, mean ± s.e.m.; *P ≤ 0.05, **P ≤ 0.01, ***P ≤ 0.001, ****P ≤ 0.0001, n.s. = non-significant; two-way ANOVA with a Tukey post-hoc test).

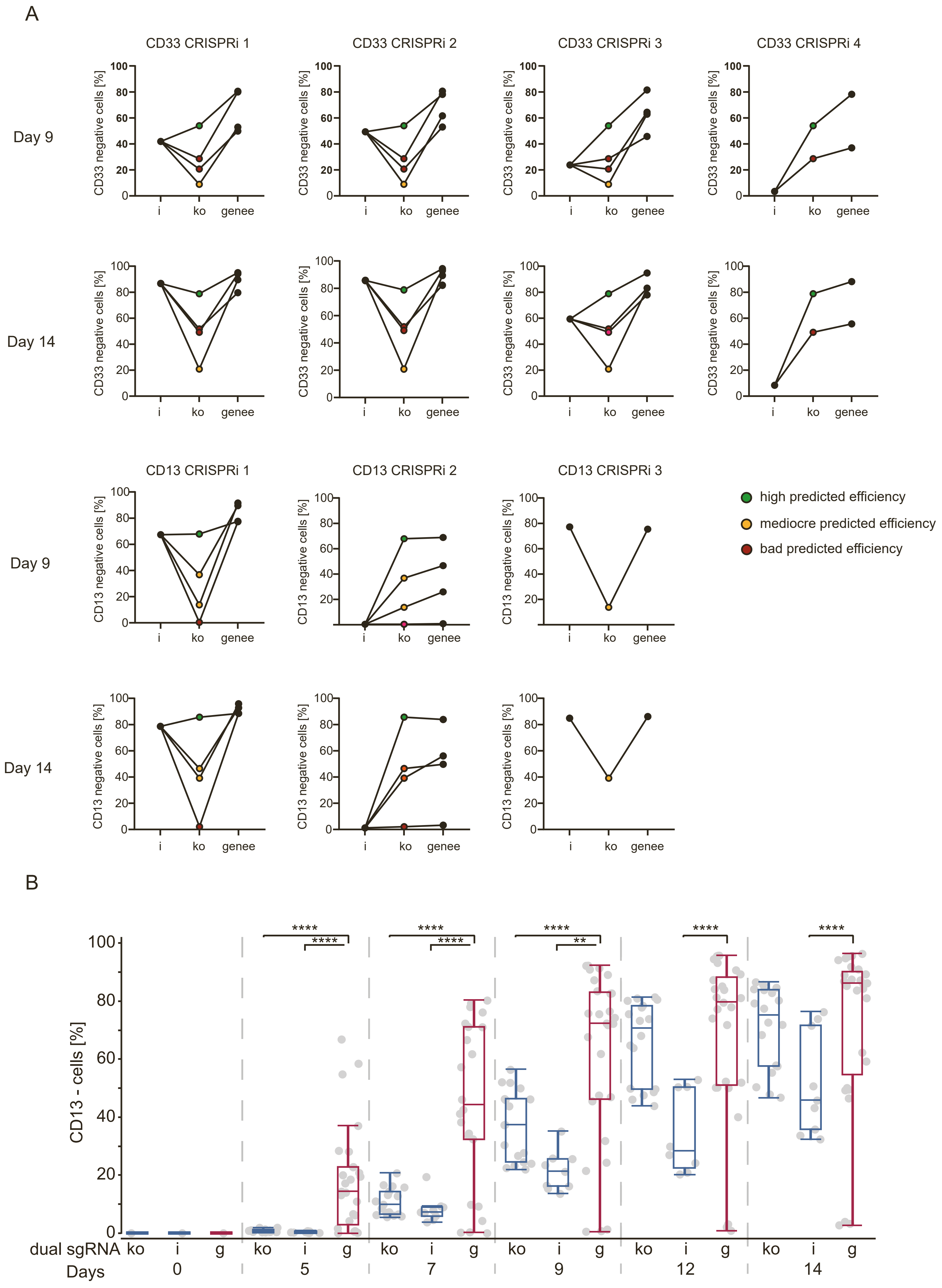
A
CD33 CRISPRi 1
CD33 CRISPRi 2
CD33 CRISPRi 3
CD33 CRISPRi 4
Day 9
Day 14
CD33 negative cells [%]
i
ko
genee
CD13 CRISPRi 1
CD13 CRISPRi 2
CD13 CRISPRi 3
CD13 negative cells [%]
high predicted efficiency
mediocre predicted efficiency
bad predicted efficiency
B
CD13 - cells [%]
****
**
dual sgRNA ko i g ko i g ko i g ko i g ko i g ko i g
Days 0 5 7 9 12 14

Figure S2: CRISPRgenee combination efficiency and comparison to dual sgRNA CRISPRi/CRISPRko. Related to Figure 1

(A) CRISPR technology combination plot, median of the percentage of CD33/CD13 negative cells for the individual sgRNAs at day 9 and day 14 from figure 1E individually split for CRISPRi, CRISPRko, and the resulting indicated CRISPRgenee combinations (n = 3).

(B) Time-resolved quantification of CD13 negative TF-1 cells expressing dual sgRNAs targeting CD13 after induction of ZIM3-Cas9 (g), Cas9 (ko) or dCas9-ZIM3 (i). Data are displayed as a single datapoint for each sgRNA combination and replicate summarized in a boxplot. (n = 3, mean, box, and whiskers min to max.; $^{**}P \leq 0.01$, $^{***}P \leq 0.001$, n.s. = non-significant; two-way ANOVA with a Tukey post-hoc test).

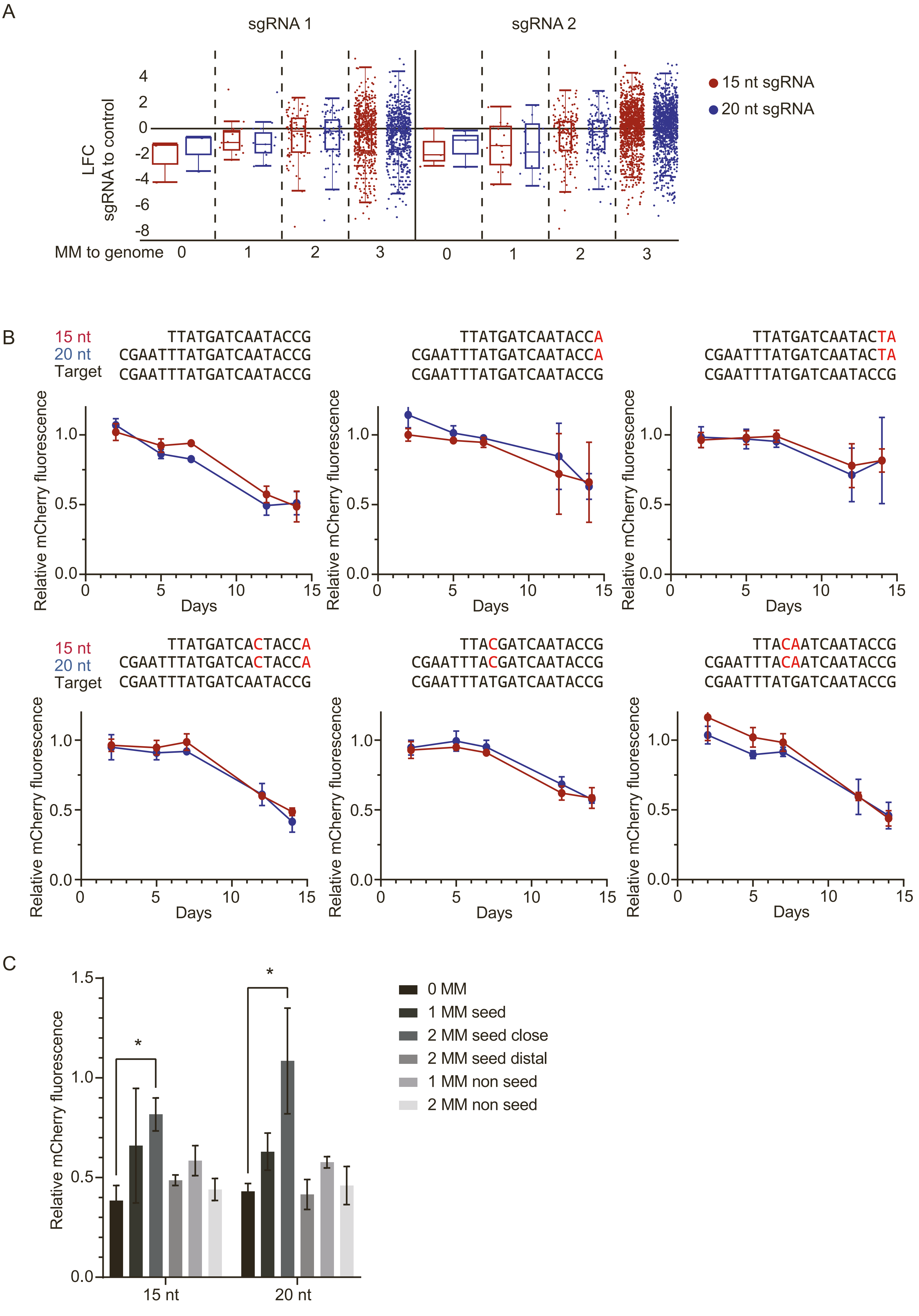
A
sgRNA 1
sgRNA 2
LFC
sgRNA to control
MM to genome
15 nt sgRNA
20 nt sgRNA
B
15 nt
20 nt
Target
TTATGATCAATACCG
CGAATTTATGATCAATACCG
CGAATTTATGATCAATACCG
TTATGATCAATACCA
CGAATTTATGATCAATACCA
CGAATTTATGATCAATACCG
TTATGATCAATACTA
CGAATTTATGATCAATACTA
CGAATTTATGATCAATACCG
TTATGATCACTACCA
CGAATTTATGATCACTACCA
CGAATTTATGATCAATACCG
TTACGATCAATACCG
CGAATTTACGATCAATACCG
CGAATTTATGATCAATACCG
TTACAATCAATACCG
CGAATTTACAATCAATACCG
CGAATTTATGATCAATACCG
Relative mCherry fluorescence
Days
C
Relative mCherry fluorescence
0 MM
1 MM seed
2 MM seed close
2 MM seed distal
1 MM non seed
2 MM non seed
15 nt
20 nt

Figure S3: Off-target analysis of the 15 nt and 20 nt sgRNA targeting a fluorescent reporter. Related to figure 2

(A) Log2 foldchange between the 15 nt or 20 nt CD33 targeting sgRNA from Figure 2 and the control sgRNA for all identified off-targets (up to 3 MM) of the 15 nt sgRNA split into groups by the MM number.

(B) Flow-cytometric analysis of the mCherry expression in TF-1 cells co-expressing the mCherry reporter, the dCas9-ZIM3 as well as the indicated sgRNAs targeting the reporter. In red is indicated the respective off-target base towards the EF1a promoter expressing the mCherry.

(C) Comparison of the relative mCherry fluorescence at day 14 between the sgRNA mismatches for the 15 nt sgRNA and the 20 nt sgRNA (n = 3, mean ± SEM.; *$P \leq 0.05$, **$P \leq 0.01$, ****$P \leq 0.0001$; two-way ANOVA with a Sidak post-hoc test).

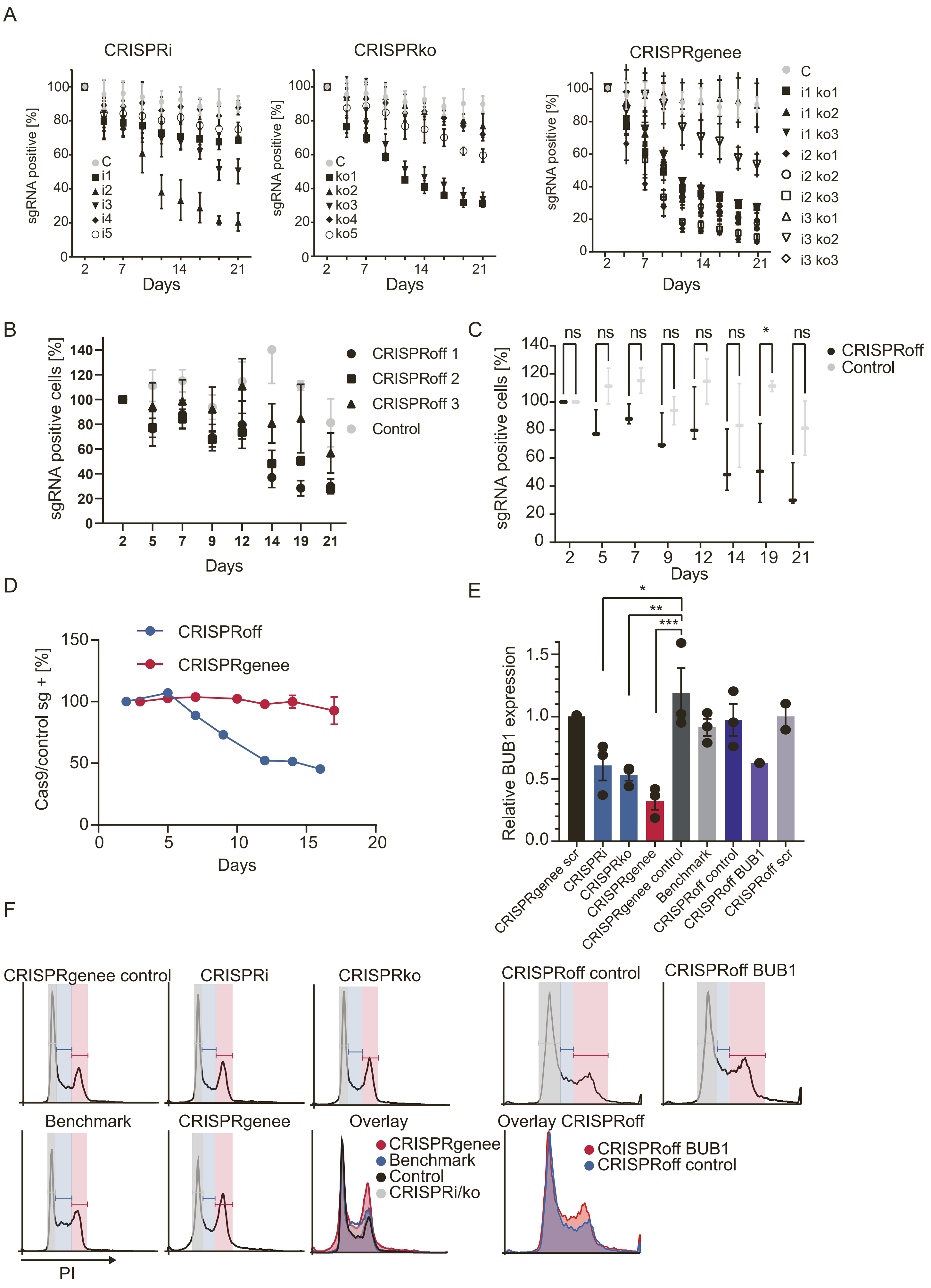

A
CRISPRi
CRISPRko
CRISPRgenee
sgRNA positive [%]
Days
C
i1
i2
i3
i4
i5
ko1
ko2
ko3
ko4
ko5
i1 ko1
i1 ko2
i1 ko3
i2 ko1
i2 ko2
i2 ko3
i3 ko1
i3 ko2
i3 ko3
B
sgRNA positive cells [%]
CRISPRoff 1
CRISPRoff 2
CRISPRoff 3
Control
C
ns
*
CRISPRoff
D
Cas9/control sg + [%]
CRISPRoff
CRISPRgenee
E
Relative BUB1 expression
*
**
***
CRISPRgenee scr
CRISPRi
CRISPRko
CRISPRgenee
CRISPRgenee control
Benchmark
CRISPRoff control
CRISPRoff BUB1
CRISPRoff scr
F
CRISPRgenee control
CRISPRi
CRISPRko
CRISPRoff control
CRISPRoff BUB1
Benchmark
CRISPRgenee
Overlay
Overlay CRISPRoff
Control
CRISPRi/ko
PI

Figure S4: Validation of single *BUB1* targeting sgRNAs and BUB1 depletion efficiency. Related to Figure 3

(A) Raw data from figure 3A showing the individual competitive proliferation assays of TF-1 cells expressing the indicated single sgRNAs as well as the respective CRISPRgenee combinations targeting *BUB1*. Shown is the relative fraction of GFP+/sgRNA+ cells relative to the initial measurement over the course of 21 days. (n = 3, mean ± s.e.m.)

(B) Raw data of the individual dual CRISPRoff sgRNAs targeting *BUB1* and the control targeting a non-essential gene.

(C) Summary of the proliferative effect of *BUB1* targeting sgRNAs for CRISPRoff and the respective control targeting a non-essential gene.

(D) Percentage of cells co-expressing CRISPRoff/CRISPRgenee and a non-targeting sgRNA control.

(E) Relative *BUB1* expression of HEK293 cells expressing sgRNAs targeting *BUB1* normalized to b-actin and a neutral control sgRNA to validate the improved CRISPRgenee effect observed in the proliferation assays. (n = 3, mean ± S.D..; *P ≤ 0.05, **P ≤ 0.01, ***P ≤ 0.001, n.s. = non-significant; two-way ANOVA with a Dunnet post-hoc test).

(F) Exemplary histograms of the P.I. staining of the individual samples and the corresponding distribution in G1- (grey), S- (blue), and G2/M-phase (red). An overlay of all histograms was plotted to further highlight the difference in cell cycle distribution.

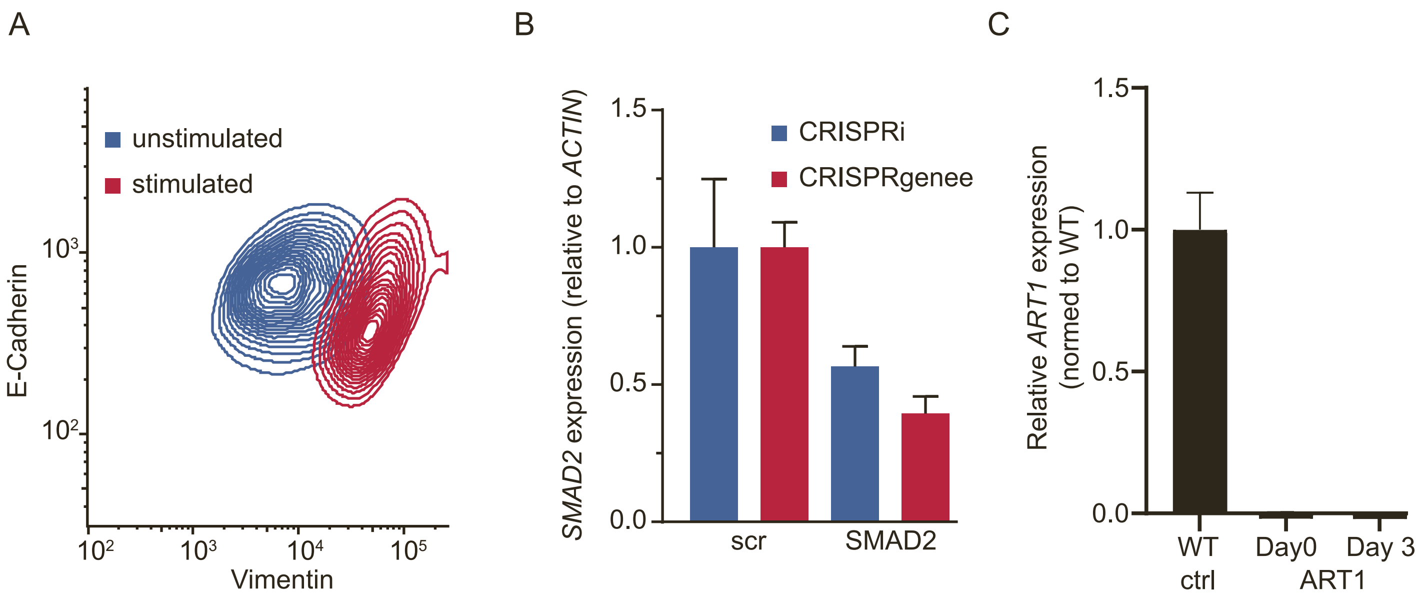
A
unstimulated
stimulated
E-Cadherin
Vimentin
B
CRISPRi
CRISPRgenee
SMAD2 expression (relative to ACTIN)
1.5
1.0
0.5
0.0
scr
SMAD2
C
Relative ART1 expression (normed to WT)
1.5
1.0
0.5
0.0
WT ctrl
Day0
Day 3
ART1

Figure S5: Staining and depletion validation for EMT and iPSCs. Related to Figure 3
(A) MCF10A cells were stained with antibodies binding to the intracellular proteins Vimentin and E-Cadherin used to identify and gate for the epithelial cell population (blue) and mesenchymal cell population (red).
(B) Relative *SMAD2* expression of MCF10A cells expressing sgRNAs targeting a non-essential control or *SMAD2* normalized to b-actin to validate the loss of SMAD2.
(C) Relative ART1 expression of unstimulated and stimulated iPSC cells expressing CRISPRgenee and a sgRNA combination targeting ART1 normalized to *b-Actin* and WT.

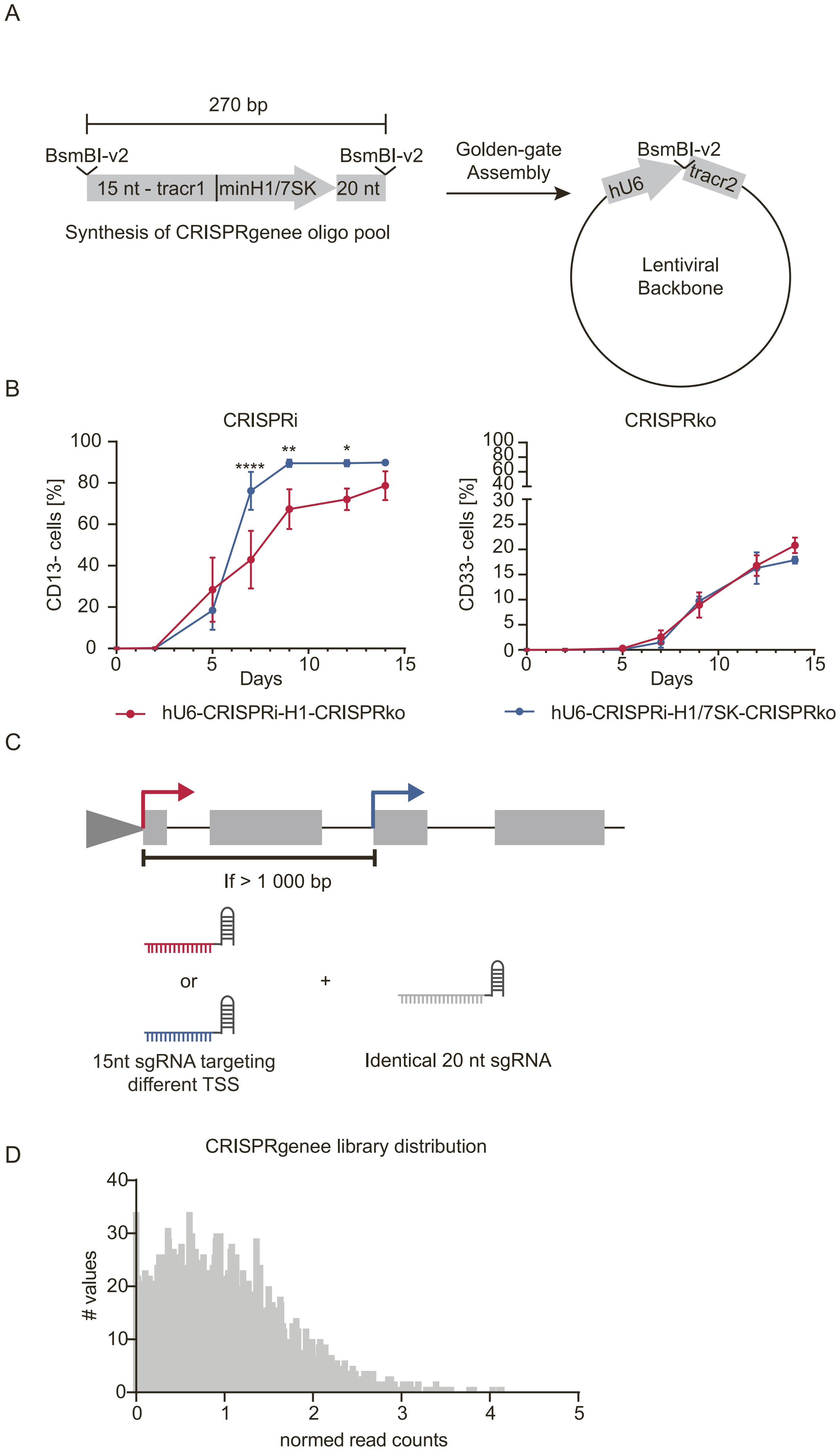
A
270 bp
BsmBI-v2
BsmBI-v2
15 nt - tracr1
minH1/7SK
20 nt
Synthesis of CRISPRgenee oligo pool
Golden-gate Assembly
BsmBI-v2
hU6
tracr2
Lentiviral Backbone
B
CRISPRi
CD13- cells [%]
Days
CRISPRko
CD33- cells [%]
Days
hU6-CRISPRi-H1-CRISPRko
hU6-CRISPRi-H1/7SK-CRISPRko
C
If > 1 000 bp
or
+
15nt sgRNA targeting different TSS
Identical 20 nt sgRNA
D
CRISPRgenee library distribution
values
normed read counts

Figure S6: CRISPRgenee library design. Related to Figure 4

(A) Oligo design of the CRISPRgenee library and the subsequent cloning strategy into the lentiviral backbone.

(B) Time-resolved quantification of dual CD13 and CD33 depletion in TF-1 cells using the hU6 promoter to express identical CD13 sgRNAs as well as the H1 or minimal H1/7SK promoter to express the CD33 targeting sgRNA (n = 3, mean ± S.D.; *P ≤ 0.05, **P ≤ 0.01, ****P ≤ 0.0001; two-way ANOVA with a Sidak post-hoc test).

(C) CRISPRgenee combination strategy for genes harbouring more than one TSS with a distance of > 1000 bp.

(D) CRISPRgenee sgRNA library distribution, measured and calculated by deep-sequencing.

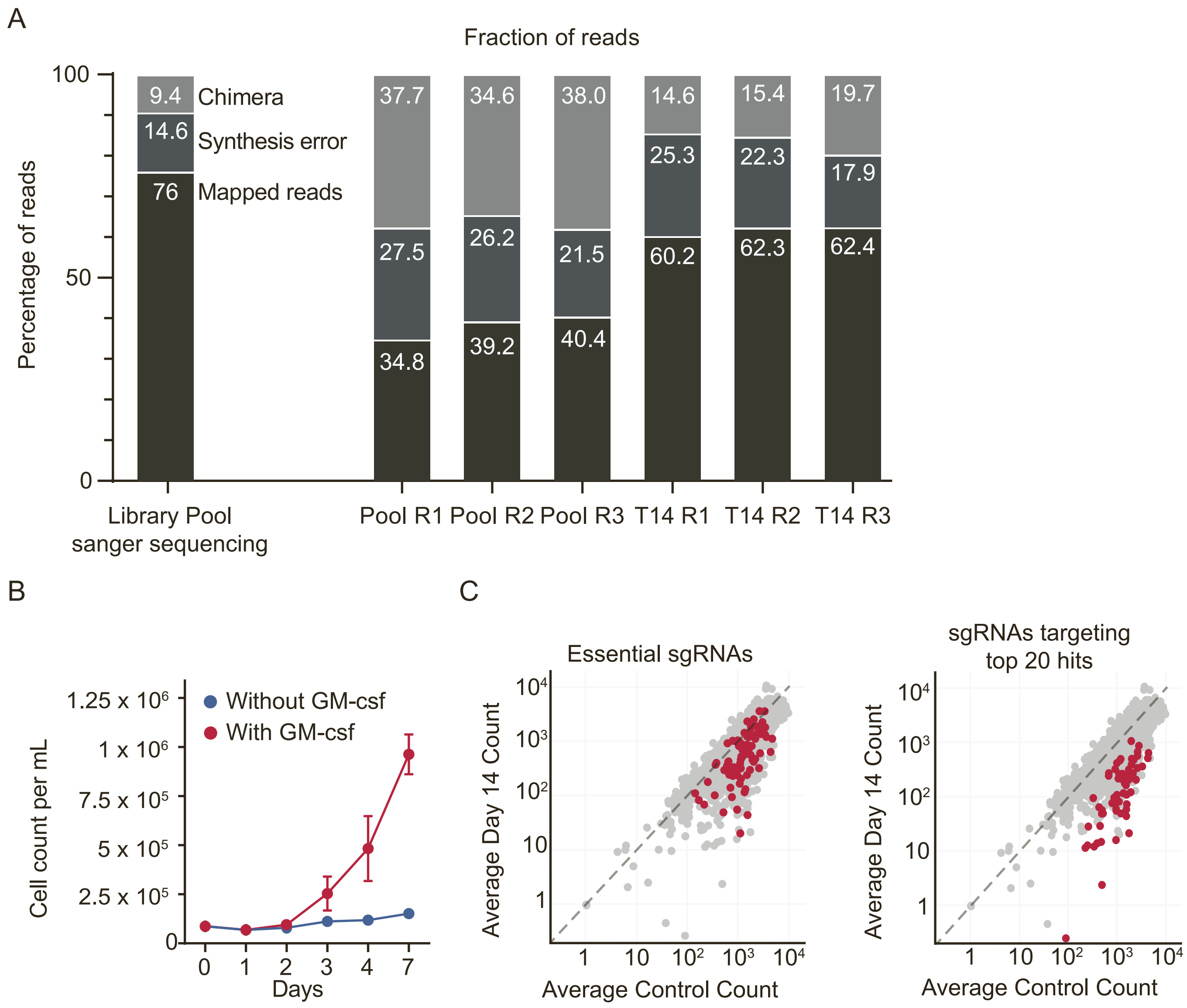
A
Fraction of reads
100
50
0
Percentage of reads
9.4
Chimera
14.6
Synthesis error
76
Mapped reads
37.7
27.5
34.8
34.6
26.2
39.2
38.0
21.5
40.4
14.6
25.3
60.2
15.4
22.3
62.3
19.7
17.9
62.4
Library Pool
sanger sequencing
Pool R1
Pool R2
Pool R3
T14 R1
T14 R2
T14 R3
B
Without GM-csf
With GM-csf
Cell count per mL
1.25 x 10⁶
1 x 10⁶
7.5 x 10⁵
5 x 10⁵
2.5 x 10⁵
0
0
1
2
3
4
7
Days
C
Essential sgRNAs
sgRNAs targeting
top 20 hits
Average Day 14 Count
Average Control Count
1
10
10²
10³
10⁴

Figure S7: Chimera rate and reproducibility of the CRISPRgenee library. Related to Figure 4

(A) Attributed mapped reads, synthesis error reads, and the chimera rate of the oligo pool replicates and the day 14 replicates of the CRISPRgenee screen after library preparation and sequencing.

(B) Proliferation of TF-1 cells grown in RPMI media supplemented with or without the growth factor GM-CSF.

(C) Left: normalized read counts of the sgRNAs targeting essential genes for the library pool after 14 days of screening. Right: normalized read counts of sgRNAs targeting the top 20 gene hits identified in the CRISPRgenee screen of the library pool compared to day 14 after induction of ZIM3-Cas9 expression.

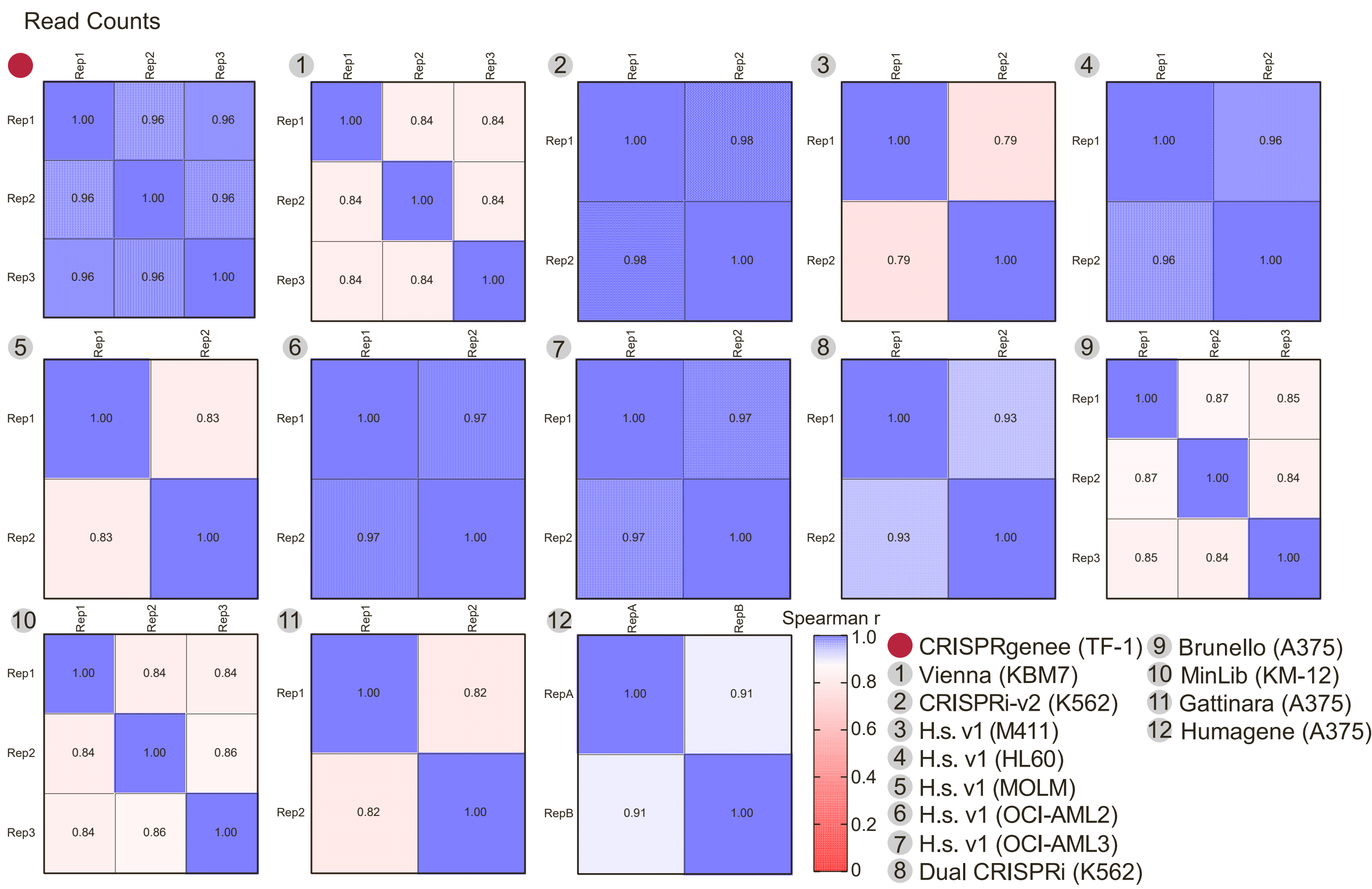

Read Counts
Spearman r
1.0
0.8
0.6
0.4
0.2
0
CRISPRgenee (TF-1)
1 Vienna (KBM7)
2 CRISPRi-v2 (K562)
3 H.s. v1 (M411)
4 H.s. v1 (HL60)
5 H.s. v1 (MOLM)
6 H.s. v1 (OCI-AML2)
7 H.s. v1 (OCI-AML3)
8 Dual CRISPRi (K562)
9 Brunello (A375)
10 MinLib (KM-12)
11 Gattinara (A375)
12 Humagene (A375)

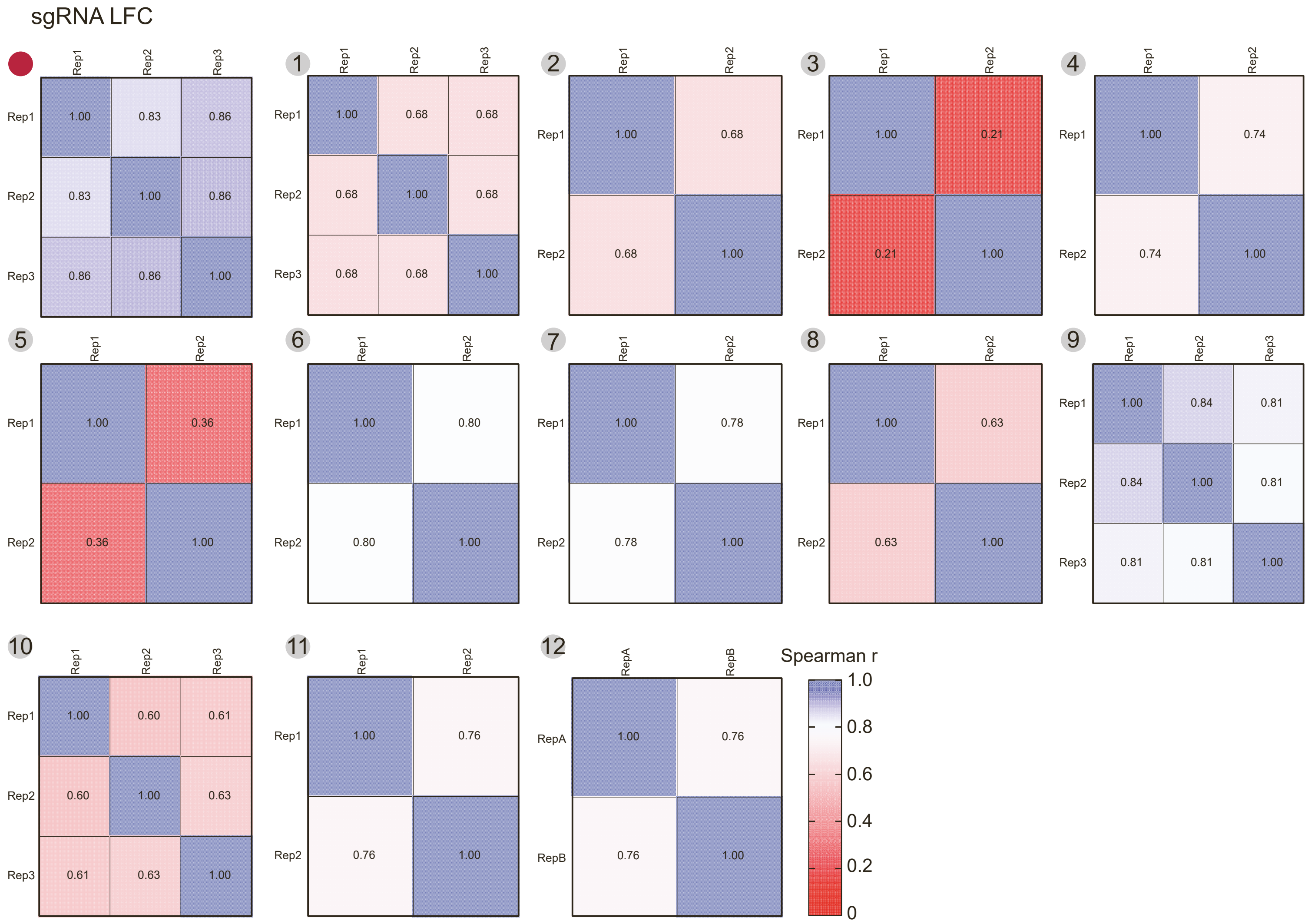

sgRNA LFC
Spearman r
1.0
0.8
0.6
0.4
0.2
0

Figure S8: CRISPRgenee achieves better reproducibility on read count and sgRNA LFC level. Related to Figure 4

Heat maps for each CRISPR screen analyzed in Figure 4D. Top: Spearman correlation of read counts of individual screening replicates for each analyzed screen. Bottom: Spearman correlation of the sgRNA LFC of individual screening replicates for each analyzed screen.

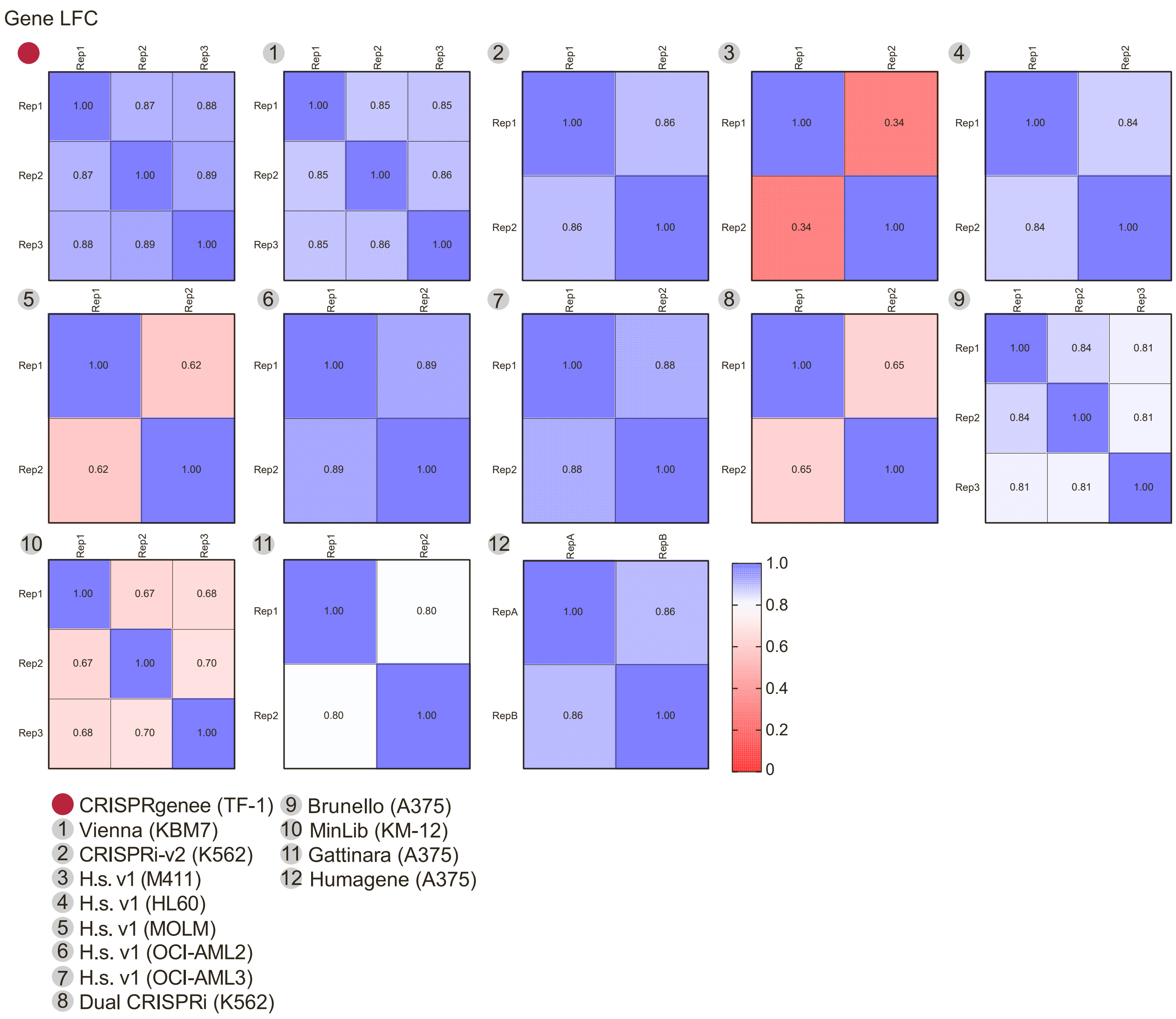
Gene LFC
Rep1 Rep2 Rep3
Rep1 1.00 0.87 0.88
Rep2 0.87 1.00 0.89
Rep3 0.88 0.89 1.00
1
Rep1 1.00 0.85 0.85
Rep2 0.85 1.00 0.86
Rep3 0.85 0.86 1.00
2
Rep1 1.00 0.86
Rep2 0.86 1.00
3
Rep1 1.00 0.34
Rep2 0.34 1.00
4
Rep1 1.00 0.84
Rep2 0.84 1.00
5
Rep1 1.00 0.62
Rep2 0.62 1.00
6
Rep1 1.00 0.89
Rep2 0.89 1.00
7
Rep1 1.00 0.88
Rep2 0.88 1.00
8
Rep1 1.00 0.65
Rep2 0.65 1.00
9
Rep1 1.00 0.84 0.81
Rep2 0.84 1.00 0.81
Rep3 0.81 0.81 1.00
10
Rep1 1.00 0.67 0.68
Rep2 0.67 1.00 0.70
Rep3 0.68 0.70 1.00
11
Rep1 1.00 0.80
Rep2 0.80 1.00
12
RepA 1.00 0.86
RepB 0.86 1.00
1.0
0.8
0.6
0.4
0.2
0
CRISPRgenee (TF-1)
1 Vienna (KBM7)
2 CRISPRi-v2 (K562)
3 H.s. v1 (M411)
4 H.s. v1 (HL60)
5 H.s. v1 (MOLM)
6 H.s. v1 (OCI-AML2)
7 H.s. v1 (OCI-AML3)
8 Dual CRISPRi (K562)
9 Brunello (A375)
10 MinLib (KM-12)
11 Gattinara (A375)
12 Humagene (A375)

Figure S9: CRISPRgenee achieves better reproducibility on gene LFC level.
Related to Figure 4
Heat maps for each CRISPR screen analysed in Figure 4D in which the gene LFC of individual screening replicates per screen were correlated using the spearman correlation analysis.

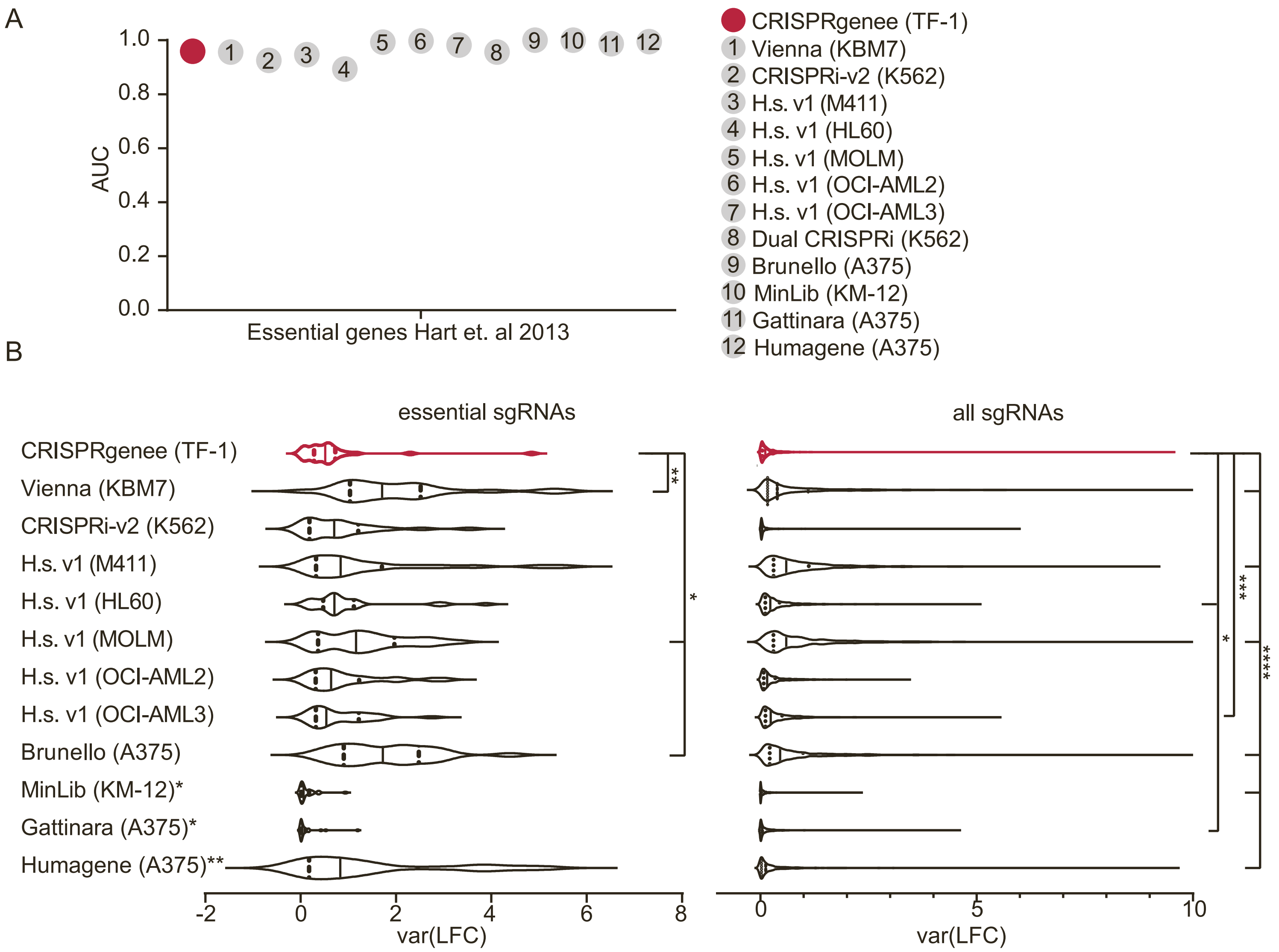

*<3 sgRNAs per gene; **enAsCas12a

Figure S10: CRISPRgenee demonstrates high sensitivity with increased consistency in sgRNA performance. Related to Figure 4

(A) The area under the curve (AUC) of ROC curves based on essential and non-essential genes for the CRISPRgenee screen and other published screens shown in Figure 4C.

(B) Violin plots comparing the variance among sgRNAs targeting the same gene observed with the CRISPRgenee system with a set of published CRISPR screening approaches. Left: Comparison of the variance of sgRNAs targeting the same genes from a subset of essential genes. Right: Comparison of the variance of sgRNAs targeting the same genes for all genes investigated in the CRISPRgenee screen.

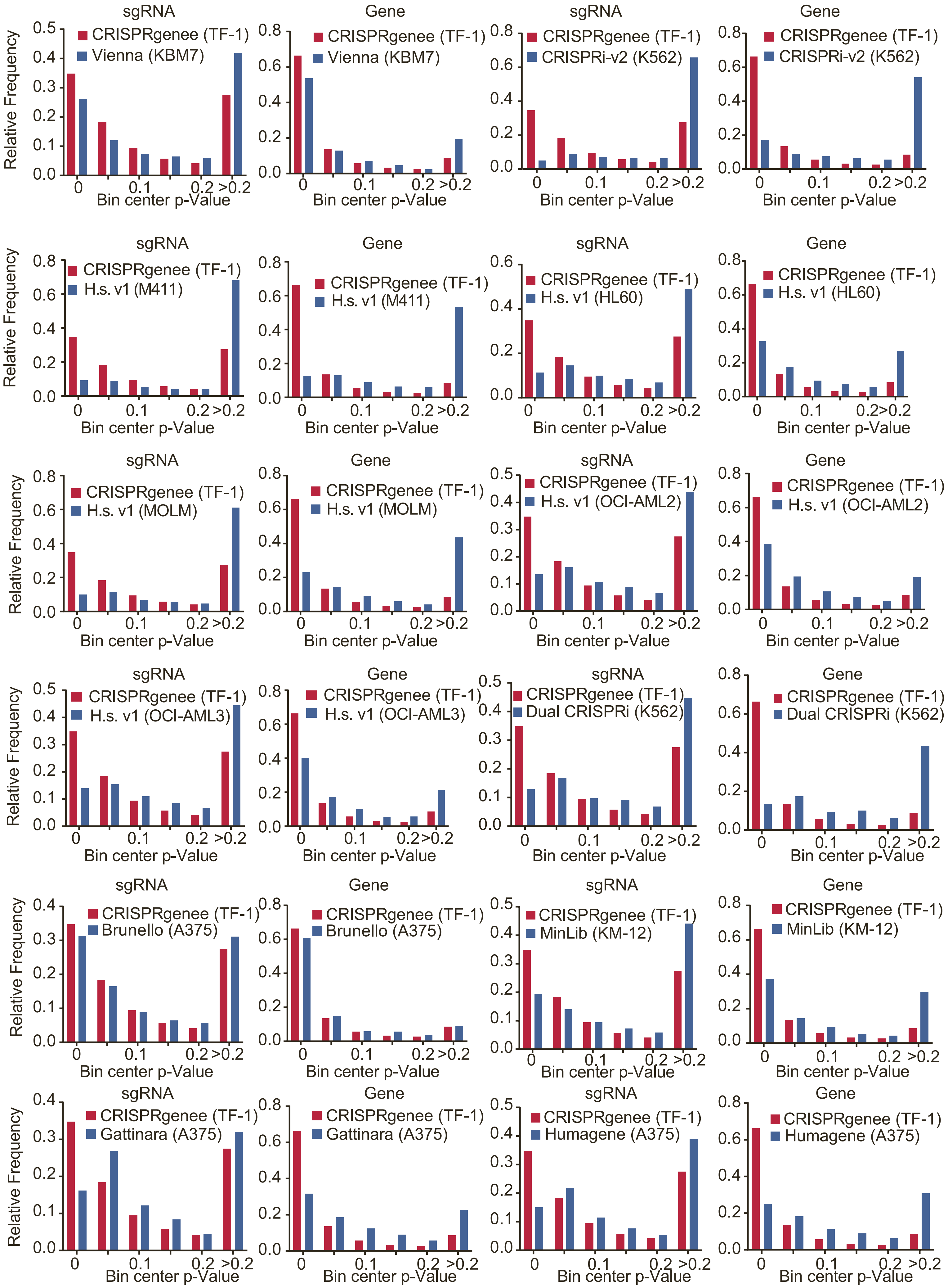
sgRNA
CRISPRgenee (TF-1)
Vienna (KBM7)
Gene
CRISPRgenee (TF-1)
Vienna (KBM7)
sgRNA
CRISPRgenee (TF-1)
CRISPRi-v2 (K562)
Gene
CRISPRgenee (TF-1)
CRISPRi-v2 (K562)
sgRNA
CRISPRgenee (TF-1)
H.s. v1 (M411)
Gene
CRISPRgenee (TF-1)
H.s. v1 (M411)
sgRNA
CRISPRgenee (TF-1)
H.s. v1 (HL60)
Gene
CRISPRgenee (TF-1)
H.s. v1 (HL60)
sgRNA
CRISPRgenee (TF-1)
H.s. v1 (MOLM)
Gene
CRISPRgenee (TF-1)
H.s. v1 (MOLM)
sgRNA
CRISPRgenee (TF-1)
H.s. v1 (OCI-AML2)
Gene
CRISPRgenee (TF-1)
H.s. v1 (OCI-AML2)
sgRNA
CRISPRgenee (TF-1)
H.s. v1 (OCI-AML3)
Gene
CRISPRgenee (TF-1)
H.s. v1 (OCI-AML3)
sgRNA
CRISPRgenee (TF-1)
Dual CRISPRi (K562)
Gene
CRISPRgenee (TF-1)
Dual CRISPRi (K562)
sgRNA
CRISPRgenee (TF-1)
Brunello (A375)
Gene
CRISPRgenee (TF-1)
Brunello (A375)
sgRNA
CRISPRgenee (TF-1)
MinLib (KM-12)
Gene
CRISPRgenee (TF-1)
MinLib (KM-12)
sgRNA
CRISPRgenee (TF-1)
Gattinara (A375)
Gene
CRISPRgenee (TF-1)
Gattinara (A375)
sgRNA
CRISPRgenee (TF-1)
Humagene (A375)
Gene
CRISPRgenee (TF-1)
Humagene (A375)
Relative Frequency
Bin center p-Value
0
0.1
0.2
>0.2

Figure S11: CRISPRgenee achieves more significant hits on sgRNA and gene level. Related to Figure 4
Histograms comparing the relative p-value frequencies of sgRNAs as well as the p-value for each gene in the CRISPRgenee screen with a set of published CRISPR screening approaches.

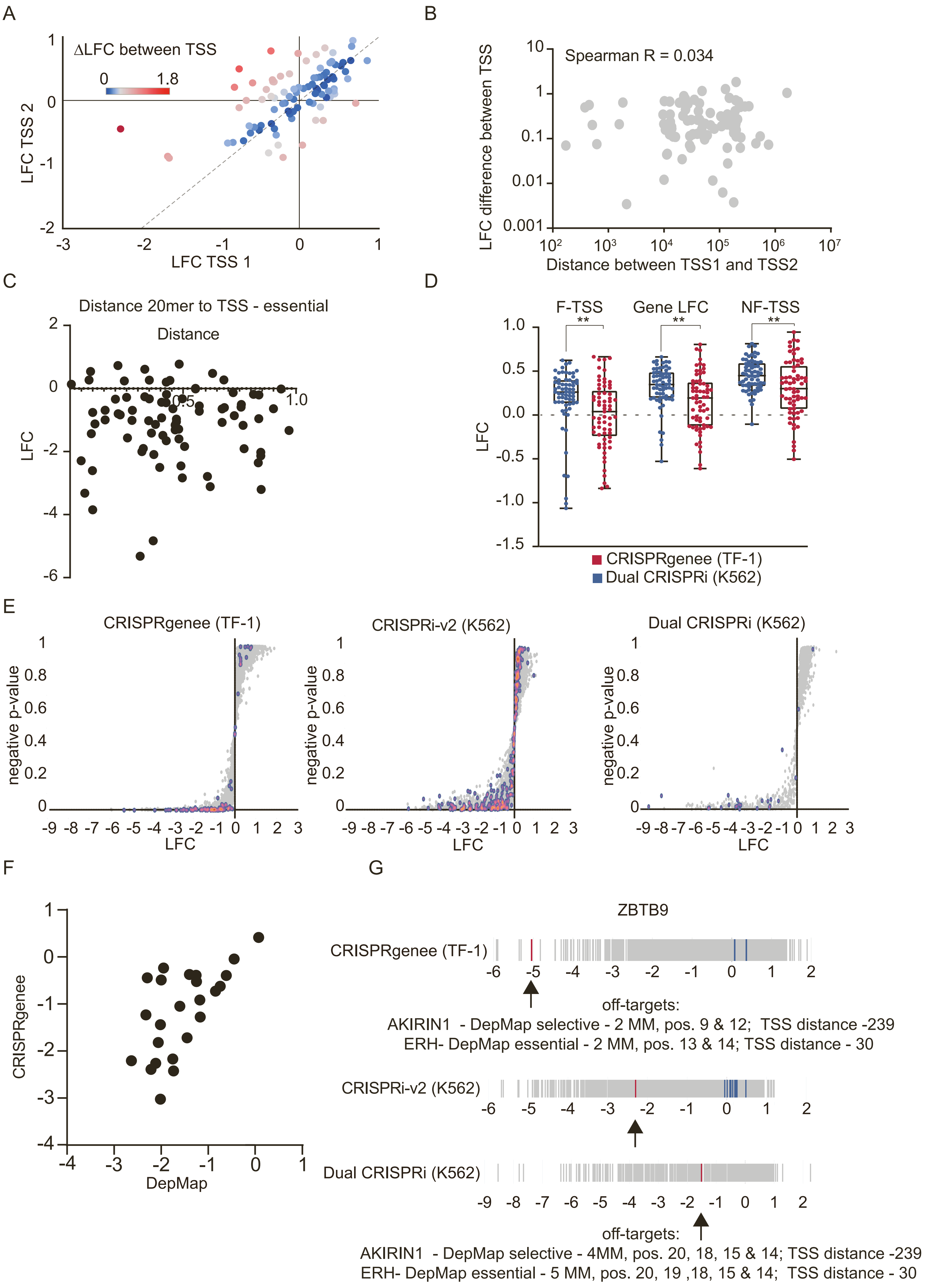
A
ΔLFC between TSS
0
1.8
LFC TSS 2
LFC TSS 1
B
Spearman R = 0.034
LFC difference between TSS
Distance between TSS1 and TSS2
C
Distance 20mer to TSS - essential
Distance
LFC
D
F-TSS
Gene LFC
NF-TSS
**
LFC
CRISPRgenee (TF-1)
Dual CRISPRi (K562)
E
CRISPRgenee (TF-1)
CRISPRi-v2 (K562)
Dual CRISPRi (K562)
negative p-value
LFC
F
CRISPRgenee
DepMap
G
ZBTB9
CRISPRgenee (TF-1)
off-targets:
AKIRIN1 - DepMap selective - 2 MM, pos. 9 & 12; TSS distance -239
ERH- DepMap essential - 2 MM, pos. 13 & 14; TSS distance - 30
CRISPRi-v2 (K562)
Dual CRISPRi (K562)
off-targets:
AKIRIN1 - DepMap selective - 4MM, pos. 20, 18, 15 & 14; TSS distance -239
ERH- DepMap essential - 5 MM, pos. 20, 19 ,18, 15 & 14; TSS distance - 30

Figure S12: CRISPRi efficiency depends on the TSS selection. Related to Figure 5 and Figure 6

(A) Scatter plot depicting the LFC of genes which have more than one TSS and are 1 000 bp distant from each other split into the cumulative LFC for sgRNAs targeting one TSS and sgRNAs targeting the other. The ΔLFC was calculated between the two TSSs and is displayed with a blue-to-red color gradient depending on the ΔLFC.

(B) Relationship between the ΔLFC of two different TSS of the same gene and the distance between both TSSs.

(C) Distance of the CRISPRko sgRNA to the TSS plotted against the LFC to identify if the CRISPRko sgRNA is also affecting gene expression through the ZIM3 domain during Cas9 binding and cleavage.

(D) The difference in LFC between the CRISPRgenee screen and the dual CRISPRi screen when targeting only the functional (F) TSS, the non-functional (NF) TSS, or the cumulative LFC for each gene.

(E) Plot of the p-value for each sgRNA over the LFC of the sgRNA for CRISPRgenee, a CRISPRi screen (16) and a dual CRISPRi screen (28). The sgRNAs targeting essential genes are displayed as density plot in red.

(F) Plot of the CRISPRgenee LFC over the DepMap score for all essential genes targeted in the CRISPRgenee screen.

(G) LFC for the CRISPRgenee sgRNA combinations targeting *ZBTB9*.

Table S1: List of all oligonucleotides used in this study for cloning, qPCR, mismatch cleavage assay, amplicon and CRISPR library sequencing. Related to Figure 1, 3 and 4 and Star Methods.

Name	Sequence (5' to 3')	Experiment
ZIM3-KRAB_Gibson_for	GGATCCACCAGCATGAACAATTCCCAGGGAAGAGTGACCTTCG	Cloning
ZIM3-KRAB_Gibson_rev	TTCTTGTCCATGGTGGCAGCGCTGATCCCGGGCCCGCGGTA	Cloning
Dual_Filler_Gibson_for	CACCTGCGTGGCACCAGA	Cloning
Dual_Filler_Gibson_rev	CACCTGCTCTCAAACGGAGA	Cloning
CRISPRoff_Gibson_for	CTTGCGTTGGATCCACCAGCCTGCAGTCGACGGTACCG	Cloning
CRISPRoff_Gibson_rev	TACCGATAAGCTTGATATCGCTAGGGCTCTTCTCCCTTC	Cloning
CD33_forward1_CTCA	CTCTTTCCCTACACGACGCTCTTCCGATCTNNNNNNNCTCAGTGAGCAGGGAACACCCCC	Amplicon Sequencing CD33 Control PCR1
CD33_forward1_TGAG	CTCTTTCCCTACACGACGCTCTTCCGATCTNNNNNNNTGAGGTGAGCAGGGAACACCCCC	Amplicon Sequencing CD33 15mer PCR1
CD33_forward1_TCGA	CTCTTTCCCTACACGACGCTCTTCCGATCTNNNNNNNTCGAGTGAGCAGGGAACACCCCC	Amplicon Sequencing CD33 20mer PCR1
CD33_reverse1	GTGACTGGAGTTCAGACGTGTGCTCTTCCGATCTCTGGGGCCGTGGGGTGATTA	Amplicon Sequencing CD33 PCR1
CD33_CD13_forward2	AATGATACGGCGACCACCGAGATCTACACTAGATCGCACACTCTTTCCCTACACGACGCTC	Amplicon Sequencing CD33 or CD13 PCR2
CD33_CD13_reverse2	CAAGCAGAAGACGGCATACGAGATATCACGGTGACTGGAGTTCAGACGTGTG	Amplicon Sequencing CD33 or CD13 PCR2
CD13_forward1_TAGG	CTCTTTCCCTACACGACGCTCTTCCGATCTNNNNNNNTAGGACCTGGGTGCTGACTATG	Amplicon Sequencing CD13 Control PCR1
CD13_forward1_GTTC	CTCTTTCCCTACACGACGCTCTTCCGATCTNNNNNNNGTTCACCTGGGTGCTGACTATG	Amplicon Sequencing CD13 15mer PCR1
CD13_forward1_GGAT	CTCTTTCCCTACACGACGCTCTTCCGATCTNNNNNNNGGATACCTGGGTGCTGACTATG	Amplicon Sequencing CD13 20mer PCR1
CD13_reverse1	GTGACTGGAGTTCAGACGTGTGCTCTTCCGATCTAGGAGATGGCGTCAAACAG	Amplicon Sequencing CD13 PCR1
qPCR_B-actin_for	AGAAAATCTGGCACCACACC	qPCR
qPCR_B-actin_rev	AGAGGCGTACAGGGATAGCA	qPCR
BUB1 Exon 23-24_for	CGATTACTTTGGGGTTGCTG	qPCR
BUB1 Exon 23-24_rev	GCTTTTGCCTTAACAAATCCA	qPCR
SMAD2_for	GGGTTTTGAAGCCGTCTATCAGC	qPCR
SMAD2_rev	CCAACCACTGTAGAGGTCCATTC	qPCR
ART1_for	GATGCCTGCTATGATGTCTCTG	qPCR
ART1_rev	AGAAGAGGTCTCGTCGTGTGA	qPCR
Syn1_for	AGCTCAACAAATCCCAGTCTCT	qPCR

Syn1_rev	CGGATGGTCTCAGCTTTCAC	qPCR
CD33_for	ACTTTCTTCCATCCCATACCCT	Missmatch cleavage assay
CD33_rev	GGATGGTTCTCTCCGTAGTCAC	Missmatch cleavage assay
CD13_for	TAGAGTGGTGGAATGACCTGTG	Missmatch cleavage assay
CD13_rev	GTAGGCAAAGGTGTGGAGGTAG	Missmatch cleavage assay
sggeneeSeq5_rev_GGAT	CTCTTTCCCTACACGACGCTCTTCCGATCTNNNNNNATCCTGTTTCCAGCATAGCTCTT	Library Aplification PCR1
sggeneeSeq7_rev_GAAG	CTCTTTCCCTACACGACGCTCTTCCGATCTNNNNNNCTTCTGTTTCCAGCATAGCTCTT	Library Aplification PCR1
sggeneeSeq8_rev_CTTG	CTCTTTCCCTACACGACGCTCTTCCGATCTNNNNNNCAAGTGTTTCCAGCATAGCTCTT	Library Aplification PCR1
sggeneeSeq12_rev_CAGA	CTCTTTCCCTACACGACGCTCTTCCGATCTNNNNNNTCTGTGTTTCCAGCATAGCTCTT	Library Aplification PCR1
sggeneeSeq13_rev_CAAC	CTCTTTCCCTACACGACGCTCTTCCGATCTNNNNNNGTTGTGTTTCCAGCATAGCTCTT	Library Aplification PCR1
sggeneeSeq14_rev_ATCC	CTCTTTCCCTACACGACGCTCTTCCGATCTNNNNNNGGATTGTTTCCAGCATAGCTCTT	Library Aplification PCR1
sggeneeSeq9_rev_CTCA	CTCTTTCCCTACACGACGCTCTTCCGATCTNNNNNNTGAGTGTTTCCAGCATAGCTCTT	Library Aplification PCR1
sggeneeSeq10_rev_CGAA	CTCTTTCCCTACACGACGCTCTTCCGATCTNNNNNNTTCGTGTTTCCAGCATAGCTCTT	Library Aplification PCR1
sggeneeSeq11_rev_CCTA	CTCTTTCCCTACACGACGCTCTTCCGATCTNNNNNNTAGGTGTTTCCAGCATAGCTCTT	Library Aplification PCR1
Fwd1_P7_read	GTGACTGGAGTTCTAGACGTGTGCTCTTCCGATCTTTGTGGAAAGGACGAAACACCG	Library Aplification PCR1
Fwd2_p7_sgDeepSeq	CAAGCAGAAGACGGCATACGAGATGTGACTGGAGTTCAGACGTG	Library Aplification PCR2
Rev2_p5_sgDeepSeq	AATGATACGGCGACCACCGAGATCTACACTCTTTCCCTACACGACGCT	Library Aplification PCR2

Table S2: List of all non-CRISPR library sgRNAs used in this study. Related to Figure 1, 2,3,6 and Supplementary figure 1,2,3,4,5,6 and 12

Target	Method	sgRNA	Related to
CD13	CRISPRko	GTGCATCCACTGCCATCACG	Figure 1
CD33	CRISPRko	GGAAGGAGCCATTATATCCA	Figure 1
CD13	CRISPRko	GCTCATGTTTGACCGCTCCG	Figure 1
CD33	CRISPRko	GAACCAGTAACCATGAACTG	Figure 1
CD13	CRISPRko	GGGGGAGTTGGCAGATGACC	Figure 1
CD33	CRISPRko	GACGCCAGGAGGAGGGATAA	Figure 1
CD33	CRISPRko	GTCCTGGGGCCCAGGGAGGT	Figure 1
CD13	CRISPRko	GTCTTCTGGAAGTGGGGTGC	Figure 1
CD33	CRISPRi	GCACCTGCCCACAGCA	Figure 1
CD33	CRISPRi	GCAGCGGCATGTCTG	Figure 1
CD13	CRISPRi	GCTCGGAACCCGCCA	Figure 1

CD13	CRISPRi	GACCCGCCAGGGTCCA	Figure 1
CD13	CRISPRi	GGCGGCGGCGCAGCT	Figure 1
CD33	CRISPRi	GCCCCTGCTGTGGGC	Figure 1
CD33	CRISPRi	GGGGCCCAGGGAGGT	Figure 1
CD33	CD33 offtarget RNA-seq sgRNA 1 20mer	CTGCTGCCCCTGCTGTGGGC	Figure 2
CD33	CD33 offtarget RNA-seq sgRNA2 20mer	GTCCTGGGGCCCAGGGAGGT	Figure 2
CD33	CD33 offtarget RNA-seq sgRNA1 15mer	GCCCCTGCTGTGGGC	Figure 2
CD33	CD33 offtarget RNA-seq sgRNA2 15mer	GGGGCCCAGGGAGGT	Figure 2
BUB1	CRISPRi	GCGGCTTCTAGTTTG	Figure 3
BUB1	CRISPRi	TGGCCATGGACACCC	Figure 3
BUB1	CRISPRko	GGTAGCAAAACAGTGTACCC	Figure 3
BUB1	CRISPRko	TGATGAATCTTGGGTCATTG	Figure 3
BUB1	CRISPRi	GGGTATTCGAATCGG	Figure 3
BUB1	CRISPRi	CTCGCCGAACGTTTC	Figure 3
BUB1	CRISPRi	AACGGCCCCGGTGAT	Figure 3
BUB1	CRISPRko	AAGGTTAATCCAGCACGTAT	Figure 3
BUB1 (First exon BM)	CRISPRko	TCCTTCAGTAAGTGTCCGTC	Figure 3
BUB1	CRISPRko	GAAACTCAAAAAATTGATGG	Figure 3
BUB1 (Last Exon BM)	CRISPRko	AGATTAGGGCCCTACGTAAT	Figure 3
ART1	CRISPRi	TTCTGAGCAGCTGTG	Figure 3
ART1	CRISPRko	GGGCCACCCCATGCTCATCG	Figure 3
Ngn2	CRISPRi	GTAGGCGTGTACGGT	Figure 3
Ngn2	CRISPRko	CGCTCACGGGCCGTCTCCCG	Figure 3
SMAD2	CRISPRi	GCACCCACCTCCCGG	Figure 3
SMAD2	CRISPRko	GCGCTTATACTACATAGGTG	Figure 3
TDRD12	CRISPRi	GCCTCCCGCACCCAC	Figure 6
TDRD12	CRISPRko	TGTGGAAGAAGATACATTTG	Figure 6
TDRD12	CRISPRko AVANA	CTATTTAGCAACTACCCAGG	Figure 6
KDM1A	CRISPRi	GGGCAGCGTGAAGCG	Figure 6
KDM1A	CRISPRko	GAATAGCAGAGACTCCGGAG	Figure 6
KDM1A	CRISPRko AVANA	GGAATAGCAGAGACTCCGGA	Figure 6
GFI1B	CRISPRi	GGCCAGTCCCGAGAG	Figure 6
GFI1B	CRISPRko	GCTGACGGAGTGCTCCAGGA	Figure 6
GFI1B	CRISPRko AVANA	CTACAAGCCTAGCTTCTCCT	Figure 6
ZBTB9	CRISPRi	CGTGTGTAACGGCGG	Figure 6

ZBTB9	CRISPRko	AGAAGCAGAGGACTGTACTG	Figure 6
ZBTB9	CRISPRko AVANA	GCATCGGCTTCAATGACACT	Figure 6
DPF1	CRISPRi	GATTTCATTCATTCT	Figure 6
DPF1	CRISPRko	GAGGCACAAAGGAAACACAC	Figure 6
DPF1	CRISPRko AVANA	GTACGTGTAAATCTGTCCCG	Figure 6
TETO-EF1as-mCherry	sgRNA shortening 20mer	TGCGGCCTGTGCAAGCGATG	Supplemental figure 1
TETO-EF1as-mCherry	sgRNA shortening 18mer	CGGCCTGTGCAAGCGATG	Supplemental figure 1
TETO-EF1as-mCherry	sgRNA shortening 16mer	GCCTGTGCAAGCGATG	Supplemental figure 1
TETO-EF1as-mCherry	sgRNA shortening 14mer	CTGTGCAAGCGATG	Supplemental figure 1
Ef1as-mCherry	mCherry offtarget analysis	CGAATTTATGATCAATACCG	Supplemental figure 3
Ef1as-mCherry	mCherry offtarget analysis	CGAATTTATGATCAATACCA	Supplemental figure 3
Ef1as-mCherry	mCherry offtarget analysis	CGAATTTATGATCAATACtA	Supplemental figure 3
Ef1as-mCherry	mCherry offtarget analysis	CGAATTTATGATCACTACCA	Supplemental figure 3
Ef1as-mCherry	mCherry offtarget analysis	CGAATTTACGATCAATACCG	Supplemental figure 3
Ef1as-mCherry	mCherry offtarget analysis	CGAATTTACAATCAATACCG	Supplemental figure 3
Ef1as-mCherry	mCherry offtarget analysis	TTATGATCAATACCG	Supplemental figure 3
Ef1as-mCherry	mCherry offtarget analysis	TTATGATCAATACCa	Supplemental figure 3
Ef1as-mCherry	mCherry offtarget analysis	TTATGATCAATACta	Supplemental figure 3
Ef1as-mCherry	mCherry offtarget analysis	TTATGATCACTACCa	Supplemental figure 3
Ef1as-mCherry	mCherry offtarget analysis	TTACGATCAATACCG	Supplemental figure 3

Ef1as-mCherry	mCherry offtarget analysis	TTACAATCAATACCG	Supplemental figure 3
ZBTB9	CRISPRi	CGTGTGTAACGGCGG	Supplemental figure 12
ZBTB9	CRISPRko	AGAAGCAGAGGACTGTACTG	Supplemental figure 12
ZBTB9	CRISPRko AVANA	GCATCGGCTTCAATGACACT	Supplemental figure 12

Table S3: List of all plasmids generated in this study. Related to star methods

EF1a-ZIM3-Cas9-P2A-GFP-PGK-Blasti	Addgene #239610
EF1a-ZIM3-Cas9-P2A-GFP	Addgene #239603
TRE3G-ZIM3-Cas9-P2A-GFP	Addgene #239605
TRE3G-ZIM3-Cas9-P2A-GFP-PGK-Blasti	Addgene #239604
sgRNA-Dual_filler(hU6_H1)-EF1a-Thy1.1-P2A-Neo	Addgene #239608
sgRNA-Dual_filler(hU6_H1/7SK hybrid)-EF1as_Thy1.1_P2A_Neo	Addgene #239609
